# Steps to build a DIY low-cost fixed-wing drone for biodiversity conservation

**DOI:** 10.1371/journal.pone.0255559

**Published:** 2021-08-13

**Authors:** Geison P. Mesquita, José Domingo Rodríguez-Teijeiro, Rodrigo Rocha de Oliveira, Margarita Mulero-Pázmány

**Affiliations:** 1 Department of Animal Biology, Plant Biology and Ecology, Autonomous University of Barcelona, Barcelona, Spain; 2 Institute Baguaçu of Biodiversity Research, São Luís, Brazil; 3 Department of Evolutionary Biology, Ecology and Environmental Sciences, University of Barcelona, Barcelona, Spain; 4 Department of Chemical Engineering and Analytical Chemistry, University of Barcelona, Barcelona, Spain; 5 School of Biological and Environmental Sciences, Liverpool John Moores University, Liverpool, United Kingdom; Institute of Botany of the Czech Academy of Sciences, CZECH REPUBLIC

## Abstract

Despite the proved usefulness of drones in biodiversity studies, acquisition costs and difficulties in operating, maintaining and repairing these systems constrain their integration in conservation projects, particularly for low-income countries. Here we present the steps necessary to build a low-cost fixed-wing drone for environmental applications in large areas, along with instructions to increase the reliability of the system and testing its performance. Inspired by DIY (Do It Yourself) and open source models, this work prioritizes simplicity and accounts for cost-benefit for the researcher. The DIY fixed-wing drone developed has electric propulsion, can perform pre-programmed flight, can carry up to 500 g payload capacity with 65 minutes flight duration and flies at a maximum distance of 20 km. It is equipped with a RGB (Red, Green and Blue) sensor capable of obtaining 2.8 cm per pixel Ground Sample Distance (GSD) resolution at a constant altitude of 100 m above ground level (AGL). The total cost was $995 which is substantially less than the average value of similar commercial drones used in biodiversity studies. We performed 12 flight tests in auto mode using the developed model in protected areas in Brazil, obtaining RGB images that allowed us to identify deforestation spots smaller than 5 m^2^ and medium-sized animals. Building DIY drones requires some technical knowledge and demands more time than buying a commercial ready-to-fly system, but as proved here, it can be less expensive, which is often crucial in conservation projects.

## Introduction

In the last decade, drones (known as Unoccupied Aircraft Systems–UAS, or Remotely Piloted Aircraft Systems–RPAS) have been adopted as a new tool for the monitoring and conservation of protected areas [1]. These systems are used for identifying deforestation and fragmentation processes [2, 3], searching for illegal hunters [4] and conducting forest inventory and biodiversity assessments [5, 6] as well as wildlife surveys [7, 8]. The success of drones for biodiversity monitoring is primarily due to the high spatial and temporal resolution of the data obtained as well as to a reduction in time, cost and logistical challenges as compared to other means of obtaining aerial imagery, such as satellite or manned aircrafts [9, 10]. The majority of biodiversity studies conducted with drones use Small Unoccupied Aircraft Systems (sUAS) weighting 2–5 kg with a wingspan smaller than 3 m and payloads below 1 kg, they are generally electrically powered and operate at low altitudes [7, 8].

Despite the growing popularity of drones, their acquisition cost along with high maintenance and training costs are the main factors constraining their use in research. In the current scenario, where the greatest loss of biodiversity is concentrated in low-income tropical countries [11], low-cost prototyping is a new way of helping local agents to preserve biodiversity [12]. Low-cost drone development initiatives (conservationdrones.org; diydrones.com) and open-source software (ardupilot.com; opendronemap.org) are gaining popularity in the drone and scientific community. DIY (Do It Yourself) models offer unlimited opportunities for researchers who need tailor-made solutions while optimizing cost-benefits [13]. ArduPilot, an open-source project combining software and hardware-plus-sensors for drones (copter, plane, rover and sub), is a positive example where sharing knowledge through DIY concepts can generate significant technologies in the scientific environment.

**Fig 1:**
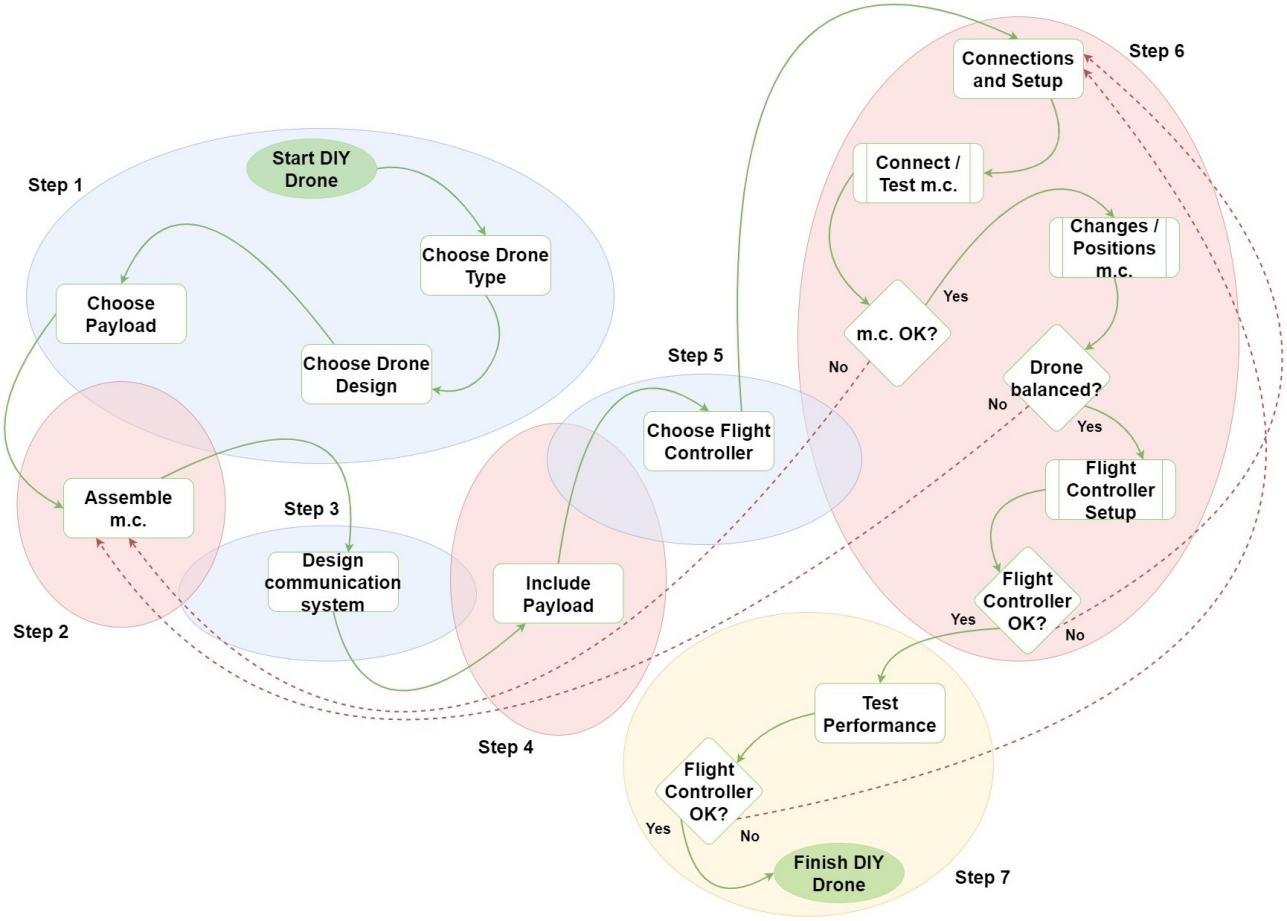
General DIY workflow. m.c. means main components. Blue balloons are pre-development phase, Red balloons are development phase and the yellow balloon is post-development phase.

Along with the significant growth in the use of drones in biodiversity conservation in the past 10 years [1, 14] some studies on building DIY fixed-wings drones for conservation purposes have been published [9, 15–19]. While these contain descriptions of the systems, they do not provide detailed information of the building process, which precludes their replication by other potential users. In the next sections we describe step-by-step the development of a low-cost, fixed-wing drone specifically designed for conservation purposes in large protected areas. It is inspired by the conservationdrones.org and diydrones.com websites, prioritizing simplicity, a positive cost-benefit balance and an open source model in the manufacturing process (Fig 1). We provide the basic construction and parameterization details in order to allow replication by individuals without prior experience in electronic works. The budget is kept to a maximum of $ 1000. We describe how we tested the performance of the model, in terms of flight autonomy, coverage and data collection with high spatial resolution. In addition, we discuss the potential uses of this model in applications aimed at monitoring protected areas and deforestation activities.

## Materials and methods

### Step 0: Choosing drone type

The first important step to integrate drones in conservation research is deciding the drone type. The drone type has to be aligned with the flight mission profile and, in order to define the mission, the scientific objectives need to be previously defined, at least regarding operation range, terrain characteristics, mission duration and payload needs.

Drones can be mainly classified into two types according to the principles of flight and aerodynamics: fixed-wing (planes) and rotary-wing (helicopters and multicopters). Fixed-wing models depend on forward motion for lift; they need to be constantly moving forward at a certain speed that can support them in the air, so that they tend to have more efficient aerodynamics. This allows longer flight durations, which makes them appropriate for working at large scales such as intended in this study. However, they require open terrain to take off and land, which may limit their use in dense vegetation scenarios. In the Rotary-wing models the engine propellers are responsible for both lift and thrust, hence the vertical component of the engine force is lift, and the horizontal component is the thrust [20]. They can support themselves in the air without a need for constant movement, which allows them to take-off and land vertically from a small patch of open terrain, and to hover in stable ways above a fixed spot in the air, generally facilitating stable data acquisition. These features make them the most popular choice for small scale (<1km^2^) biodiversity studies [21] that track specific targets or obtain data at fixed points. A few hybrid models exist, although they are generally expensive.

**Fig 2:**
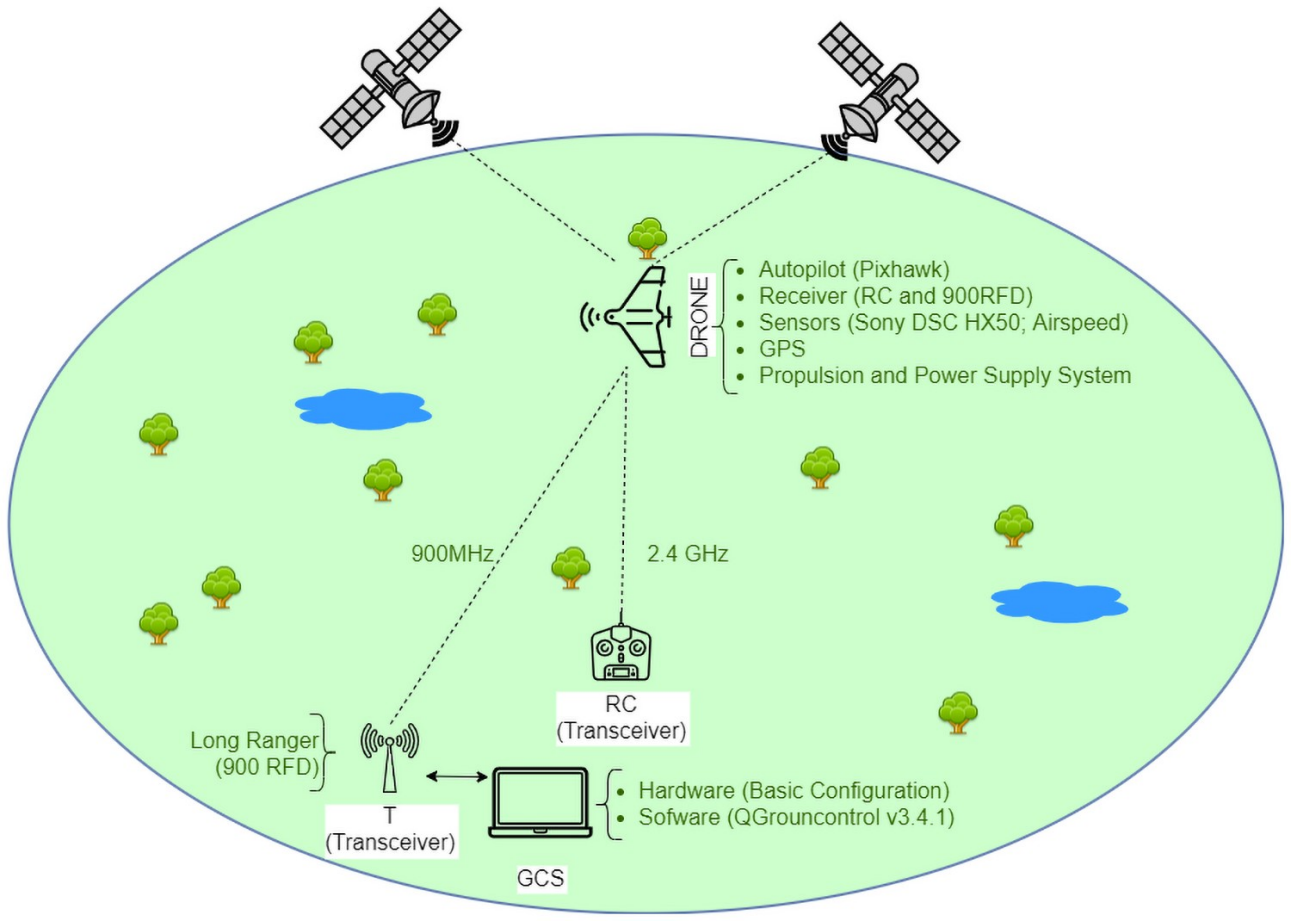
Schematic of the system’s four main components.

Regardless of the drone type, previous studies suggest that the minimal requirements for drones in conservation works are: 1) ability to fly in manual and Global Navigation Satellite System (GNSS)-aided modes as well as in pre-programmed mode; 2) easy transportation and pre-flight assembly; 3) payload capacity of up to 500 g; 4) 30 minutes minimum flight duration; 5) and at least 5 km telemetry range [7, 9, 21, 22]. Most drones used for conservation purposes that need to perform pre-programmed flights have four main components: Ground Control Station (GCS), Telemetry (T), Radio Controller (RC) and the drone platform (Fig 2).

### Step 1: Choosing fixed-wing airframe design

Within the fixed-wing drone there is a variety of models, sizes and shapes with different maneuverability, performance and advantages [23]. Among the commercial, most used fixed-wing airframes in conservation studies available on the market, we highlight the tailless or delta-wing (eBee, SenseFly; UX5, Trimble; UX11, Delair; Batmap II, CloudUAV; Maptor, Horus) and the typical gliders (Maja, Bormatec; HBS Skywalker, HornbillSurveys; RQ-11, AeroVironment; E384, EVENT38 Unammaned Systems). While glider-type models generally possess more control surface configuration options thanks to a tail (aileron, flaps, elevator, rudder), delta-wing or tailless models have less control surface options as these features are absent (elevon, rudder or equivalent). These different airframe configurations, along with other factors (size, payload weight, propulsion system) directly influence the velocity needed to maintain flight, flight duration and maneuverability.

In fixed-wing models, the wing aspect ratio is one of the factors that increases the aerodynamic efficiency. High wing aspect ratio confers a smaller induced drag component that results in an enhanced gliding, leading to energy saving. In general, the lower the lift-to-drag ratio is proportional to the size of the airframe. Therefore, it requires a higher thrust needed to overcome aerodynamic drag at a given lift and this associated drag and power penalty causes a reduction in total energy efficiency [20]. On the other hand, a smaller airframe can bring some benefits for maneuverability, which can be important to facilitate eventual pilot interventions. Portability is another important aspect when choosing airframes for conservation works. While smaller airframes are easy to take off and transport, larger airframes generally require more space and logistics to take off, landing and transportation.

Taking into account the wing aspect ratio, size, portability and also the price, we chose the airframe of a typical glider, characterized by a high wing aspect ratio, a slender fuselage and a fully-faired narrow cockpit. This model is one of the most efficient aerodynamic designs and its features minimize induced drag for any given amount of lift [24]. The airframe model used was the fixed-wing Ranger 2000 (Volantex-RC, CO., International) with the following features: 2000 mm wingspan, 1100 mm length, 1083 g empty weight (See Item 1.1 in S1 Text). The fuselage is made of hard, flexible plastic, the wings are composed of expanded polyolefin (EPO) and the control set includes four servos (ailerons, flaps, rudder and elevator). Due to the limited internal space of the fuselage, and in order to reduce RGB (Red, Green and Blue) sensor stability problems, we modified the internal fuselage structures in order to fit the electronic components and sensors, as well as to fit the lens of the RGB imaging sensor at the bottom of the fuselage (See Item 2 in S2 Text). This allowed us to carry any payload fitting in a volume of 12 x 5 x 7 cm^3^. This model is easily launched by hand and recovered by “belly landing”, avoiding the need for complex systems such as catapults or skyhooks. Another aspect considered important in the choice of this model was its portability: it is modular and can be disassembled into three smaller parts (fuselage, wings and elevator, and rudder) that allow transportation inside one compact case (110 x 30 x 30 cm^3^).

### Step 2: Assembling primary electronic components

There are three possible options for purchasing an airframe: 1) Almost Ready-to-Fly (ARF), where the airframe is purchased without the primary electronic components (motor, servos, ESC, battery, etc.); 2) Plug-N-Play (PNP), where the airframe comes with all the primary components installed, except for the battery, receiver and transmit; and 3), Ready-to-Fly (RTF), where all the primary components are already installed on the airframe. The choice of the airframe version, in addition to being directly related to the intended purpose of the drone, must take into account the knowledge level of those involved in the process of assembling the drone, the degree of customization that is intended to be performed on the model and the time available for the process. In our case, we opted for the airframe model PNP version, since we intended to use differentiated batteries and communication system. It included a brushless electric engine, six servos, a brushless Electronic Speed Control (ESC) and 8 x 4 propellers (See Item 1 in S1 Text). For simplicity, we used the default configuration of the engine, ESC, servos and propeller. The recommended battery for the pre-installed motor was a 3S 2200 mAh / 25C / 11.1 V LiPo battery, but we replaced it for a higher capacity 4S 5000 mAh / 25C / 14.8 V LiPo battery in order to increase flight time (See Item 1.6 in S1 Text). There is no standard formula defining the balance between battery capacity, weight and flight time, but it is necessary to consider several factors (type of flight, airframe model, wing load, engine and propeller) to find an optimal compromise. Currently, the majority of drones used in conservation-related works use an electric propulsion system [23].

### Step 3: Designing the communication system

There are several types of drone communication systems, from short-range, unidirectional communication through a simple RC, to more complex long-range communication systems with robust GCS. The DIY system we designed includes three different communication links: one unidirectional (GNSS) and two bidirectional ones (RC and telemetry). The Ublox M8N GNSS module (See Item 1.9 in S1 Text) is indispensable to autopilot flight and geo-referencing because it determines the drone’s real time location in 3D by means of triangulation, the RC FlySky model (See Item 1.12 in S1 Text) with an approximate range of 1 km, features a 2.4 GHz transmitter and server to perform manual control of the drone when necessary, and the telemetry with the RFD 900 long-range radio modem model (See Item 1.10 in S1 Text) at 915 MHz has an approximate range of 40 km. Considering the minimum configurations above, we chose the link models on the market with the best cost-benefit ratio.

### Step 4: Selecting the payload

The usefulness of research drones is determined by their payload [25]. The payload of the model described here is formed by one sensor, a compact RGB camera that we used to acquire high resolution images. We opted for a Sony model DSC-HX50 that can gather images in the visible spectrum with high resolution and records Full HD 1080p video (See Item 1.13 in S1 Text). We used the Seagull #MAP2 UAV camera trigger to connect the Sony camera to the flight controller and the RC receiver (See Item 1.14 in S1 Text).

### Step 5: Selecting the flight controller

The Flight Controller or Autopilot is considered the “drone brain”. There are two types of commercial autopilot solutions available: closed-hardware and open-hardware. Following the open-source and low-cost solution in this study, we chose an open hardware autopilot (See Item 11 in S5 Text). Aiming at the most favourable cost-benefit ratio and possibilities of updating the core code in the future, we chose the mRo Pixhawk 2.4.6 (mRobotics.io) open hardware flight controller board (See Item 1.8 in S1 Text). This model is an enhanced version of the discontinued Pixhawk 1 (3DR Robotics Inc) that uses the firmware (FMUv3) with twice the flash memory of the Pixhawk 1. The mRo Pixhawk is a microcontroller with several internal sensors (gyroscope, accelerometer/compass; magnetometer and barometer) that serves as a communication center and connection of sensors (speed sensor, cameras, lasers, among others). In order to increase the efficiency of pre-programmed flight we incorporated an airspeed sensor. The airspeed sensor we used was the pitot tube airspeedometer model, that measures differences in air pressure and helps the autopilot to control the drone under different flight conditions as well as for autonomous landings (See Item 1.11 in S1 Text). The flight controller board can be used on different platforms (fixed-wing, rotary-wing, rover, boats, submarines and others). We chose an open source flight control, the PX4 software that enables the programming and execution of fully autonomous drone flights and is fully compatible with the mRo Pixhawk 2.4.6 model. The entire system is divided into two parts: 1) the hardware and on-board firmware installed on the drone; and 2) the software installed on the GCS. Different flight controllers can be controlled by different GCS software packages that have different interfaces.

There are about 10 different GCS software that can be installed on desktops or tablet / smartphones (See Item 12 in S5 Text). Among these, we limited our-selves to 4 GCS open source licenses (Mission Planner, APM Planner 2.0, MAV Proxy, QGroundControl) that have more configuration and analysis tools, important features for DIY users. Although the Mission Planner is the GCS recommended by Ardupilot, as it was the first to be created and has more features, we opted for QGroundControl because it is the only one with the possibility to run on all platforms (Windows, Mac OS, Linux, Android and IOS), it has an intuitive interface, allows automatic download of the correct firmware for a connected autopilot (based on its firmware) and provides a full flight control and vehicle setup for Pixhawk and ArduPilot. The QGroundControl interface Pixhawk allows creating flight missions with waypoints and performing other flight commands via radio and telemetry. However, the pilots’ choice of GCS software is a matter of preference since the features of GCS software are similar.

### Step 6: Connections and setup

Once all hardware and software has been decided in the previous steps, it is necessary to start the configuration process while considering the premise of simplicity, that is, the fewer modifications, the better. At this step we suggest configuration sequences that should follow the order presented here. For each of these sequences, we provide detailed information in the (S2 and S3 Texts), according to the DIY concept.

#### I. Configuring and testing the main components

Considering that the PNP airframe version was purchased, the motor, servos and ESC components are already pre-installed on the airframe, so there is no need for any modifications. However, it is necessary to check if all these components are working correctly as well as to eliminate possible problems during parameterizations of the flight controller or even during the flight (See Item 1.1 in S2 Text).

#### II. Component positioning and modifications

The position of the internal components will directly influence the drone balance and, consequently, the flight performance. Therefore, it is necessary to define the positioning of all components and possible modifications of the airframe considering the drone balance from the center of gravity. The modifications we made to the airframe (hole at the bottom for passing the camera lens, hole at the top for passing the GNSS cable and elimination of some internal structures for positioning the battery and flight controller) were carried out taking into account the balance of the drone (See Item 2 in S2 Text). Some fixed-wing airframe models have markings that indicate the center of gravity where the drone can be balanced and are usually found below the wings. We recommend that the center of gravity of each airframe is found and the modifications and positioning of the components are carried out from there (See Item 13 in S5 Text).

#### III. Setup flight controller

The mRo Pixhawk 2.4.6 flight controller is the command center of the drone that makes the link between the main components, the sensors and the GCS, so its configuration is one of the most important parts in the drone development. Initially we must make the connections on the Pixhawk of all the components necessary for its operation (See Item 1.2 in S2 Text). Then we must perform the installation of the GCS software to start the update, calibration and setup of the internal and external sensors of the flight controller (See Item 2 in S3 Text).

**Fig 3:**
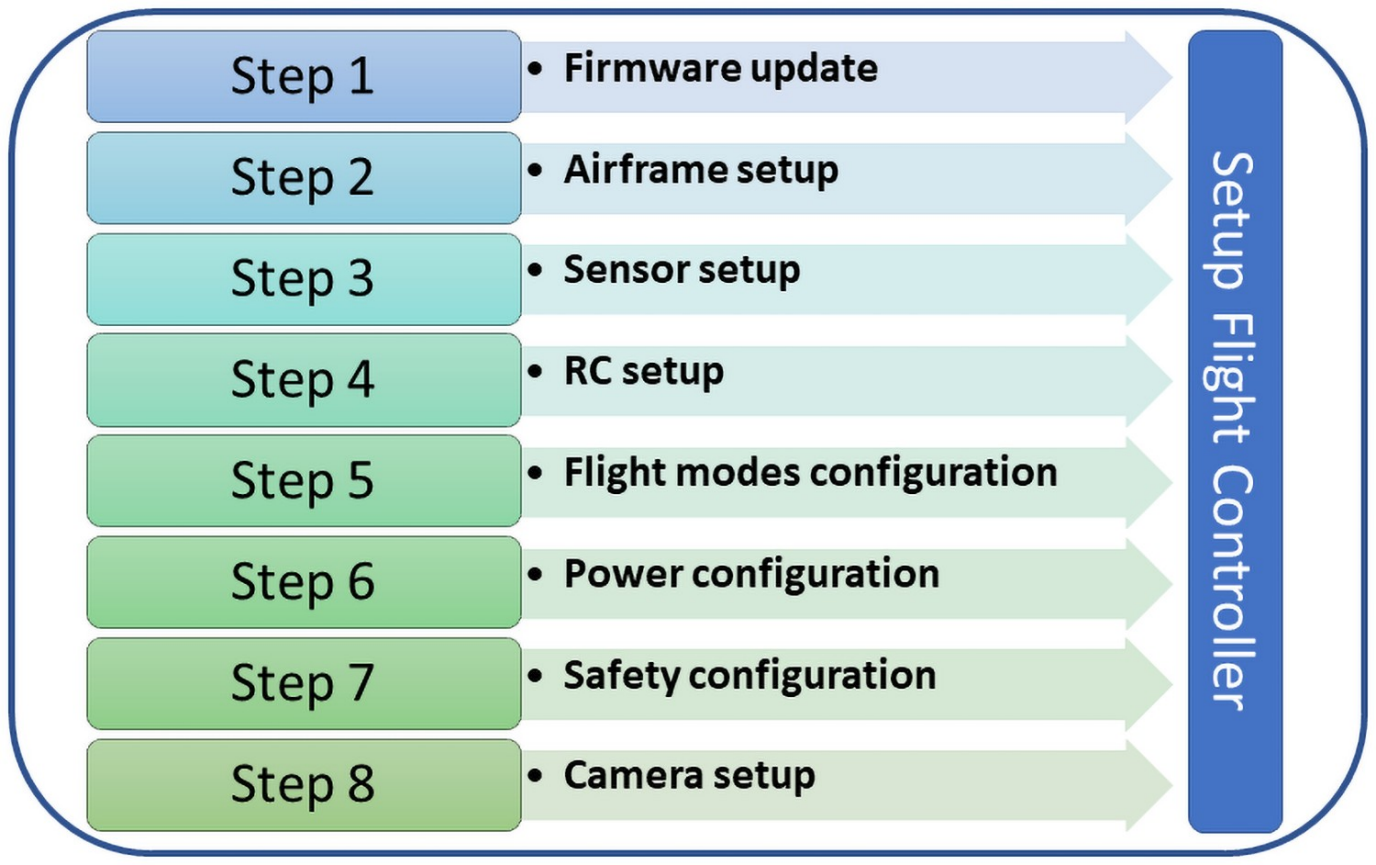
Steps for setup flight controller.

Once the components are connected and the GCS software is installed, we start the following steps (Fig 3): Firmware update (See Item 3 in S3 Text); Airframe setup (See Item 4 in S3 Text); Sensor setup (See Item 5 in S3 Text); RC setup (See Item 6 in S3 Text); Flight modes configuration (See Item 7 in S3 Text); Power calibration (See Item 8 in S3 Text); Safety configuration (See Item 9 in S3 Text); and Camera setup (See Item 10 in S3 Text) within the QGroundcontrol software. We recommend that the processes mentioned above are performed in the same order in which they appear, since in some test configurations some problems occurred when performed differently.

During the execution of each of these processes, we may encounter some unusual situations concerning the configuration of the flight controller (See S4 Text).

### Step 7: Performance tests

Performance tests were carried out for proving the drone’s ability to cover an area of up to 1 km^2^ with a minimum spatial resolution of 3 cm / px with only one battery. The drone flight tests were performed in July 2018, 15:00–18:00 h local time in different areas within the northern region of Maranhão state, Brazil. We performed 16 test flights (four conducted in manual mode and 12 in auto mode) to assess flight autonomy, communication range and resolution of aerial images. Before flight missions we followed safety procedures regarding the operator, drone stability and the protection of others involved (See S1 Checklist).

During the four flights in manual mode, we tested the aerodynamics, control surfaces (ailerons, flap, rudder and elevator) and engine. All manual flight manoeuvres were performed in VLOS (Visual Line of Sight), <500 m from GCS, and the take-off and landing were performed manually. The 12 flights in auto mode aimed to check the autopilot, pre-programmed flights, the telemetry RFD 900, GNSS and the other external sensors (compact RGB camera and 3DR Digital Airspeed). All auto mode flights were performed in VLOS or EVLOS (Extended Visual Line of Sight).

For EVLOS flights to test RFD 900 long-range telemetry, it was necessary to have a second pilot with a second RC connected (binding) to the drone’s transmitter. In these flights, this pilot followed all trajectories of the drone beyond the visual line of sight of the main pilot, by moving parallel to the drone’s trajectory by car. For this, we strategically choose an open field adjacent to the road outside an urban area that allowed the second pilot to travel by car as well as to watch the drone during the entire flight path. In direct communication with the main pilot, the second pilot was able to manoeuvre the drone with the second RC in case of any eventual problem. This type of logistics on EVLOS flight tests was necessary both to avoid the loss of the drone due to possible connection and failsafe failures and to comply with local civil aviation legislation. Although the second RC signal was always within the range of 500 m throughout the trajectory of the EVLOS test flights, we disabled the Failsafe action in QGroundControl in case of RC loss signal, to avoid the automatic return to home of the drone in long-range flights and we enabled the Failsafe action in case of long-range telemetry signal loss. We opted for the execution of “Return mode” action in situations of telemetry RFD 900 loss for more than 10s (See Item 9 in S3 Text).

For comparison with other DIY drones, we performed a simple transect flight and lawnmower flight pattern simulating methodologies employed in studies using DIY drones [9, 17]. For transect flights, within the QGroundControl, we programmed a "Corridor Scan" flight pattern consisting of a straight-line flight with a maximum length and telemetry distance of 20 km. The drone was programmed to fly at a constant altitude of 100 m (AGL) and at a speed of 15 m/s. The "Corridor Scan" flight pattern was performed twice within the same area and with the same parameters (See flight-plan-corridor-scan in S1 Plan). In these flights, we mainly tested the flight range and the maximum telemetry range (Table 1).

**Table 1:** Main pre-programmed flights features.

Pre-programmed Flights Mode							
Flight	Pattern	Flight Type	Altitude (m)	Wind speed (m/s)	Range (km)	Flight time (min)	GSD (cm/px)
1	Corridor Scan	EVLOS	100	10	15	65	2,8
2	Corridor Scan	EVLOS	100	9	17	50	2,8
3	Circular Grid	VLOS	25	11	0,1	13	0,7
4	Circular Grid	VLOS	25	8	0,1	13	0,7
5	Circular Grid	VLOS	50	10	0,1	8	1,4
6	Circular Grid	VLOS	50	9	0,1	7	1,4
7	Circular Grid	VLOS	75	12	0,1	6	2,1
8	Circular Grid	VLOS	75	10	0,1	6	2,1
9	Circular Grid	VLOS	100	9	0,1	5	2,8
10	Circular Grid	VLOS	100	9	0,1	4	2,8
11	Specific Grid	EVLOS	120	8	2	25	3,3
12	Specific Grid	EVLOS	120	9	2	27	3,3

All flights were performed with a 70% overlap (front and side) and 15m/s drone speed. Wind speed was obtained using the UAV Forecast app.

For the lawnmower flight we programmed a "Circular Survey" flight pattern covering 10 ha (lat: -2.524484° / long: -44.208837°) at 100, 75, 50 and 25 m AGL (See flight-plan-lawnmower in S1 Plan) which was performed in VLOS in order to identify the best flight altitude for distinct objectives (monitoring of anthropic activities, vegetation analysis, fauna and flora identification). The camera was triggered automatically using the “Survey Mode” (See Item 10 in S3 Text) and based on a predefined flight plan to produce at least 70% overlap and side lap among each image. We performed two test flights for each altitude in the same area applying the same parameters, totalling eight flights (Table 1).

For the last two test flights, we programmed a lawnmower flight with a grid pattern covering around 1 km^2^ in a specific area inside the “Area de Proteção Ambiental do Itapiracó” (lat: -2.523079°, long: -44.202738°). The flights were carried out at 120m AGL, maximum altitude permitted by the local civil aviation legislation, and at 15 m/s (Table 1). The camera was also triggered automatically using the “Survey Mode” and with 70% overlap and side lap among each image. These flights were planned to support the “Secretaria de Meio Ambiente do Maranhão–SEMA” in identifying degraded areas, opening of trails and unauthorized access within part of the “Area de Proteção Ambiental do Itapiracó”. In addition to identifying trails in these flights, we tested the drone ability to generate useful data for creating orthoimages, georeferenced maps and other products. For these flights, two observers with direct communication with the pilot were positioned at opposite extreme points within the flight plan grid for constant observation of the drone (EVLOS flights).

### Ethical statements

The flight tests followed Brazilian Civil Aviation Special Regulations (RBAC-E No. 94). The local civil aviation legislation does not allow BVLOS flights (Beyond Visual Line of Sight) without prior special registration and authorization of the drone, the flight and the pilot. Therefore, all flight tests were performed in VLOS or EVLOS mode as reported above.

**Fig 4:**
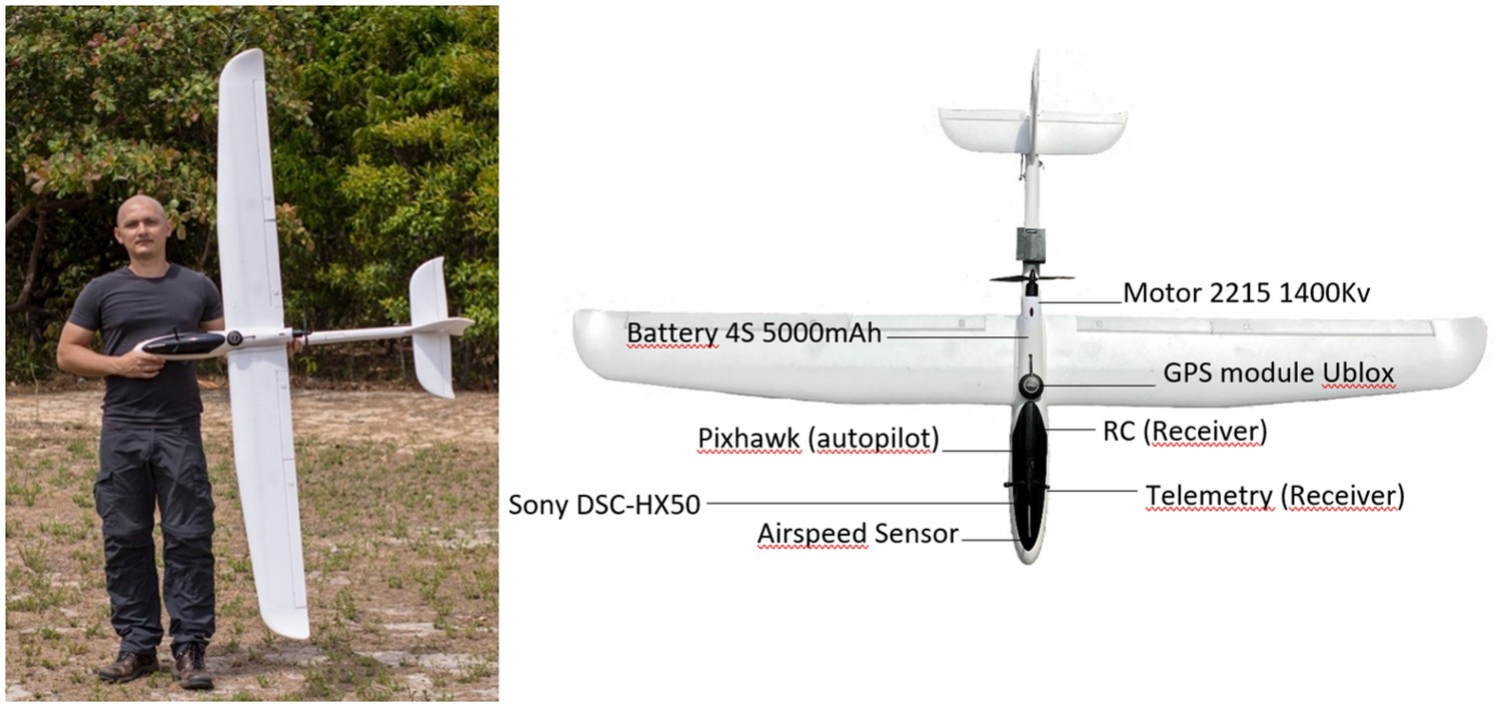
Asa-Branca-I model and main components.

The individual shown in Fig 4 has given written informed consent (as outlined in PLOS consent form) to publish his picture in this study.

## Results

We completed the development of the DIY model named “Asa-Branca-I” (Fig 4) in five months, with 30 hours of weekly dedication for development, plus another month for all performance tests and final adjustments. The purchase process and delivery time of the components corresponded to 20% of the development time, considering that the majority of the components were shipped from China and delivered to Spain.

The model developed in this project had a material cost of $995 (details in Table 2). The average price of small fixed-wing commercial drones used in conservation studies that could perform similar functions to the model developed is around $15797 and, for equivalent DIY drone models where cost information is available, this figure is around $1440 (S1 Table).

**Table 2:** Asa-Branca-I costs (USD) based on prices available on the internet in November 2019.

Specifications model UAS “Asa-Branca-I”			
Component	Model/Brand	Quantity	Cost ($)
Airframe	Volantex Ranger 2000 (PNP version)	1	135
Motor	Motor 2215 1400 Kv	1	*
Servos	Servos 9 g	6	*
Propeller	8 x 4	1	*
Electronic Speed Control	ESC 30 A 2-4S XT60 Volantex	1	*
Battery	Turnigy 5000 mAh 4S 14.8 V	1	25
Charger	SkyRC IMAX B6 Digital	1	36
Autopilot	Pixhawk PX4 2.4.6	1	130
GNSS	Ublox NEO-M8N GPS Module	1	16
Telemetry	900 RFD 915 MHz	1	176
Sensor	Pitot Tube Airspeedomoter	1	38
RC	Flysky FS-i6X 2.4 GHz 10CH RC Transmitter	1	51
Camera	Sony DSC-HX50	1	310
Camera trigger	Seagull #MAP2 + cable Sony	1	38
Accessories	Connectors and cables	-	50
TOTAL			995

Ground station laptop cost not included.

* Included in the airframe cost.

The manual and pre-programmed flight tests allowed us to adjust manoeuvrability, payload capacity, flight duration and range so that we could confirm they were suitable for being used for biodiversity studies in large areas. We accomplished pre-programmed flights with a maximum flight time of 65 minutes, including take-off and autonomous landing. With simple transect pre-programmed flights as made in other conservation studies [9, 17], the model was able to fly for 50 minutes, covering a total distance of 42.4 km round trip, at a speed of 15 m/s, at a constant altitude of 100 m AGL and a maximum telemetry range of around 20 km, covering an area of 1.7 km^2^ with 2.8 cm px^-1^ GSD. As a reference, the model in circular survey pre-programmed flights was able to survey 10 ha in four minutes flying at 100 m AGL (2.8 cm px^-1^ images); six minutes flying at 75 m AGL (2.1 cm px^-1^ images); seven minutes flying at 50 m AGL (1.4 cm px^-1^ images); and 13 minutes, flying at 25 m AGL (0.7 cm px^-1^ images) always including take-off and landing (Table 1).

**Fig 5:**
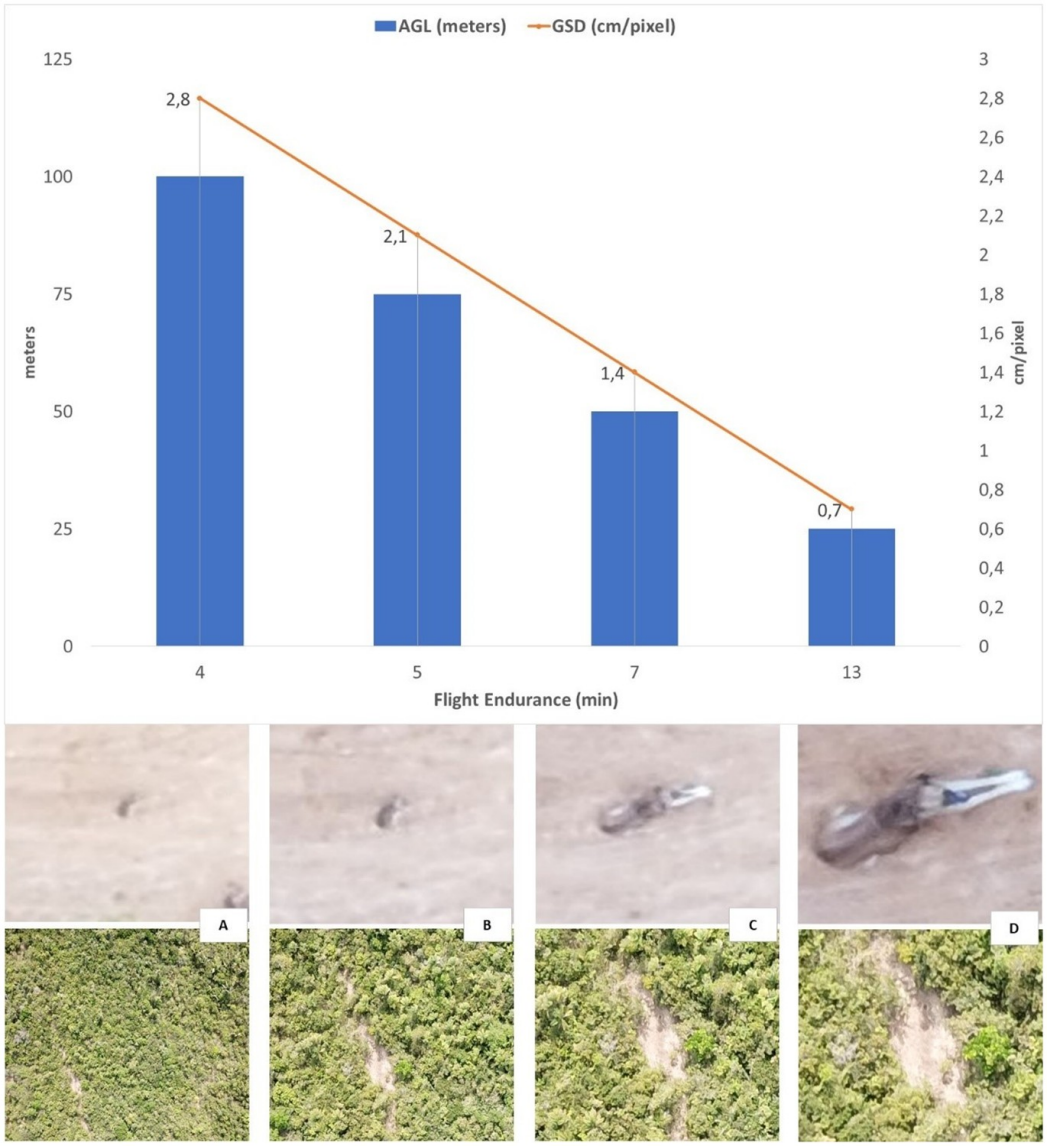
Comparison between Above Ground Level (AGL), Ground Sample Distance (GSD) and flight duration. Images with a 10x zoom of a dog and a deforestation spot at (a) 100 m; (b) 75 m; (c) 50 m; and (d) 25 m AGL.

For both specific and circular grid for the lawnmower flight at 100 and 120 m AGL it was possible to identify deforestation spots smaller than 5 m^2^, opening of small trails and vegetation clearings that are difficult to detect by satellite images (Fig 5). We could also easily detect and identify medium size animals (such as a domestic dog, 1 m size) on the images obtained in flights with the embarked camera at 25 m AGL, which were less easily detectable although still noticeable at around 50 m.

**Fig 6:**
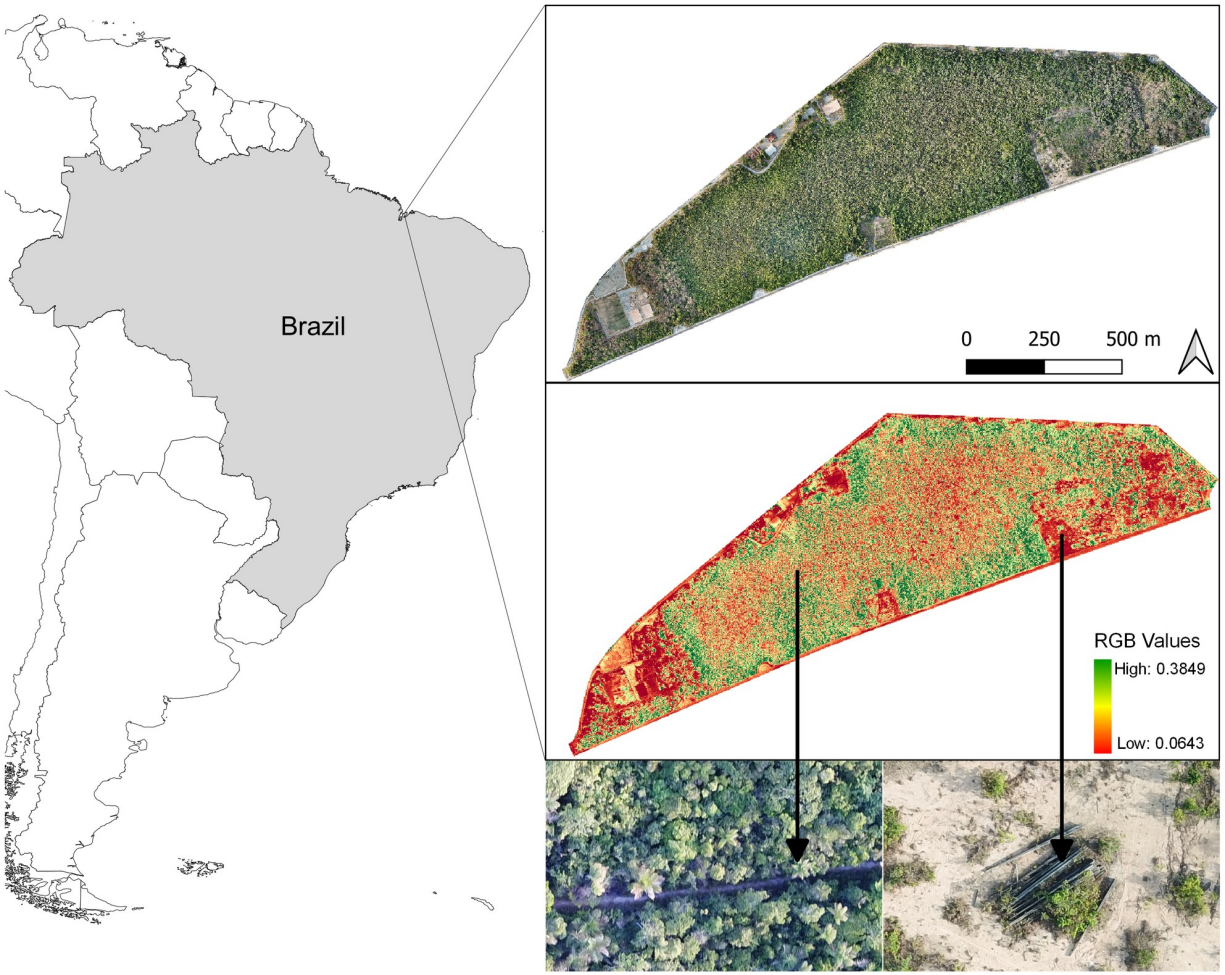
Georeferenced orthomosaic and health vegetation map. Images with a 20x zoom of an open trail in the vegetation and a deforestation area with some irregular structures.

For the last two flights with a specific grid pattern and at 120 m AGL it was possible to obtain images with a resolution of 3.3 cm px^-1^ in which we could identify trails in the vegetation and degraded areas inside an area of 1 km^2^. The drone images were processed in this case to create an orthomosaic map using the Agisoft Metashape (version 1.5.5; www.agisoft.com). In addition to an orthomosaic map, we also created maps to analyze the quality of vegetation, known as healthy green vegetation or plant health maps. These maps show how green the images are through the Visible Atmospherically Resistant Index (VARI), since this index is used for images obtained from an RGB sensor (Fig 6). The vegetation health map was created from images of the visible spectrum (Red, Green, Blue) obtained by the RGB sensor using the formula: VARI_green_ = (R_green_−R_red_) / (R_green_ + R_red_−R_blue_) [26]. Knowing that the images obtained are from the visual spectrum, we considered the standard CIR Calibration of Agisoft Metashape and filtered the values obtained between 0.06 and 0.39 of each raster.

## Discussion

In this study we describe the step-by-step process for the development of a DIY low-cost drone that allows performing basic biodiversity-related studies in large areas. We present the steps in a simple and flexible way, aiming to help researchers with basic electronics knowledge and with limited financial resources to develop their own drone system. We describe the drone developed in this project as low-cost since the summed amount of the components was $995, substantially less than the average value of commercial drones, even in relation to other DIY drones used in biodiversity studies [See S1 Table]. The reduction of drone acquisition costs in conservation projects produces a significant saving in the total budget, but there are additional factors to consider when evaluating the DIY option against commercial products. While the material cost of the drone developed here is $14802 less than commercial drones’ average, it took us five months to develop it and an additional month to perform the tests presented in this study. Among the few studies that describe the development of a low-cost fixed-wing drone in the last decade [9, 18, 19, 27, 28] there is no information on the average development time, which makes it impossible to compare our results with other studies. This fact makes the development time informed here a useful parameter for the development of future projects. Therefore, when choosing between developing your own drone system and purchasing a commercial RTF system, the time required for development must be considered in addition to the final cost.

As in other studies that use low-cost fixed-wing drones to carry out conservation works [4, 15, 16, 19], we also chose for this project the development of the drone system of the glider-type platform due to its better aerodynamic efficiency, portability and competitive cost. Despite the choice of this platform, the development process is not limited to the specific platform model presented here. Once the main components and the way to make their connections and configurations are defined, these can be easily mounted into other fixed-wing platforms without many adjustments or even translated to rotary-wing platforms, although with some more modifications. While the development of the first DIY model requires a substantial initial time investment, thereafter only occasional updates are necessary for building new DIY platforms, even for different goals.

Through simple transect and lawnmower flights tests, we verified that our model served for monitoring large areas within a 20 km radius, covering more than 1 km^2^ in a single flight at high spatial resolution, which is sufficient to perform standard vegetation analysis [29, 30], fauna identification [10, 31] and deforestation monitoring [2]. Particularly for deforestation monitoring, the ability of this model to flight long distances, enabling large coverage with high spatial resolution, makes it a low-cost technology tool with a great potential for combating illegal activities in protected areas, especially in the Cerrado biome, one of the world’s biodiversity hotspots [32] which is suffering a drastic loss of native vegetation during the last years [33].

The maximum flight time and coverage capacity of this model was similar to that of commercial RTF systems such as the standard version of eBee model (SenseFly), one of the most well-known fixed-wing systems in the drone market, and which presents an average acquisition cost 15 times higher than the model developed in this project. Beyond the economic factor, other important factors, still little considered in the comparisons between the use of drones and manned airplanes, are the environmental impacts and the social costs concerning greenhouse gas emissions. One of the first attempts to analyse these costs showed that monitoring areas of up to 30 km^2^ using photometry with a resolution of 5 cm / px from fixed-wing drones is more economically and socially advantageous than the use of manned aircrafts [34]. Considering that the comparisons in that study were made between the costs of manned aircraft and the eBee model, representing the fixed-wing drones, the economic advantage of DIY drone models as the one developed in this project is emphasised.

Although nowadays there are satellite systems such as DETER [35] used in Amazon and Cerrado biomes monitoring with a greater potential to identify changes in forest vegetation cover in areas measuring between 25 and 100 ha, with a spatial resolution of between 56 and 64 meters, the system developed here can identify changes in vegetation cover at a scale of meters and with spatial resolution at a scale of centimetres. By conducting only two lawnmower flights, it is possible to monitor the entire protected area (3.2 km^2^) of the “Área de Proteção Ambiental do Itapiracó” where we conducted part of our tests. In addition to this fine resolution scale, the possibility of systematic replication (temporal resolution) and the non-interference of cloud cover are other advantages over monitoring via satellite images that make the use of these types of drones an efficient tool in the inspection and fight against deforestation of large protected areas. The combination of payload and flight procedures developed here also allowed us to identify medium-sized fauna species from 50 m AGL, which suggests that it can also be useful for conducting medium-sized terrestrial wildlife studies [e.g. 4, 31].

The data obtained with the developed drone served to generate products such as orthomosaic maps and vegetation health maps that allowed monitoring the degradation of vegetation in protected areas with a higher resolution than satellite derived ones. The advantages of implementing drones, instead of satellites or manned aircrafts for generating orthomosaic maps with centimetre resolutions and all other subsequent products [27] makes photometry with drones one of the current main resources in the activities of conservation and combating environmental degradation, such as identification [36, 37], mapping [38, 39] and monitoring [6, 40]. In addition to the performance tests, we also validated the functionality of the Asa-Branca-I model in two environmental inspection and monitoring actions carried out by public environmental organizations in Maranhão, Brazil. These actions allowed to identify illegal opening of trails used by hunters within protected areas in the Cerrado biome that were not identified in previous terrestrial inspections due to the difficulty of ground access to the site.

Although the performance tests demonstrated here the suitability of the low-cost drone developed to cover large areas, we note that the local legislation, which generally follows the international legislation, ended up being a limiting factor regarding the use of this model and all other commercial models with similar functions, for the monitoring of large areas beyond the visual line of sight of the pilot, on flights known as BVLOS. Thus, seeking the certifications for the developed model as well as for the pilot, are future steps that should be considered for those who intend to use the full potential of drones capable of long-range flights as the model produced in this work.

## Conclusions

Finding solutions that can make environmental monitoring more efficient is a constant challenge for researchers and conservationists. The balance between costs and benefits is one of the key factors for choosing between buying a commercial drone or developing a DIY solution. In this paper, we described a path for the development of a low-cost drone and performed tests to prove its usability. With a material cost considerably lower than the least expensive model on the market, the knowledge gained from the development of this drone could be an alternative for researchers with limited financial resources. We are aware that the model developed here is just a first version with many possibilities for improvement. In addition, further tests in different situations and with different objectives are necessary to validate large-scale drone capacity. Transforming this model into a vertical take-off and land (VTOL) model, in order to make take-off and landing operations easier, increasing the stability of the camera with gimbal insertion and attaching safety features such as a parachute, are improvements that we intend to incorporate in future versions. Therefore, we believe this DIY model approach can be a valuable alternative for conservation projects.

## Supporting information

S1 Table Small fixed-wing commercial drones’ features used in conservations studies. (DOCX)

S1 File DIY drone Asa-Branca-I documentation. (TXT)

S1 Text Components list. (DOCX)

S2 Text Building DIY drone—Assembly instructions and connections. (DOCX)

S3 Text Setting up—Guidelines on settings. (DOCX)

S4 Text Tips and troubleshooting. (DOCX)

S5 Text Help and learning links. (DOCX)

S1 Checklist Pre-flight checklist. (DOCX)

S1 Plan Flight plans. (RAR)
