# Supplementary material for: Steps to build a DIY low-cost fixed-wing drone for biodiversity conservation
Source: PLoS One. 2021 Aug 13;16(8):e0255559. doi: 10.1371/journal.pone.0255559 (PMC8363011; doi:10.1371/journal.pone.0255559)
Supplement: S1 Table — (DOCX) [file pone.0255559.s001.docx]

**S1 Table.** **Small fixed-wing commercial drones’ features used in conservations studies**. * Ground station laptop cost not included. **International Standard Atmosphere (ISA) conditions at sea level.

|  | **Manufacturer** | **Drone Model *** | **Endurance (min)**** | **Range (km)** | **MTOW (kg)** | **Sensor Resolution (mpx)** | **Sensor Size (mm)** | **RGB Sensor brand** | **Cheapest configuration** | **References** |
| --- | --- | --- | --- | --- | --- | --- | --- | --- | --- | --- |
| **COMERCIAL** | AeroVironment | RQ-11 | 90 | 10 | n/d | n/d | n/d | Advanced EO/IR | $ 35.000,00 | [1,2] |
|  | Trimble | UX5 | 35 | 5 | 2,9 | 36 | 35.9 x 24.0 | Sony a7R | $ 24.895,00 | [3,4] |
|  | Falcon Unmanned | Falcon | 60 | 10 | n/d | 24 | 23.5 x 15.6 | Sony Next 7 | $ 20.000,00 | [5,6,7] |
|  | senseFly | eBee | 59 | 5 | 1,4 | 20 | 12.75 x 8.5 | S.O.D.A | $ 14.990,00 | [8,9,10] |
|  | Delair | X100 | 45 | 5 | 2 | 10,4 | 7.44 x 5.58 | Ricoh GR Digital III | $ 8.000,00 | [11,12] |
|  | HornbillSurveys | HBS SkyWalker | 45 | 20 | 1,5 | 12,1 | 7.44 x 5.58 | Canon S100 | $ 4.000,00 | [13,14] |
|  | Bormatec | Maja | 60 | 5 | 3 | 12,1 | 7.44 x 5.58 | Canon S100 | $ 3.700,00 | [15,16,17] |
| **DIY** | DIY I | SkyWalker Airframe | 60 | n/d | 0,8 | 12,1 |  | Canon SX230 HS | $ 1.380,00 | [18] |
|  | DIY II | EasyFly Airframe | 50 | 10 | 2 | 11 |  | Panasonic Lumix LX3 | $ 1.500,00 | [19] |

**References**

1. Dulava S, Bean WT, Richmond OMW. Environmental Reviews and Case Studies: Applications of Unmanned Aircraft Systems (UAS) for Waterbird Surveys. Environmental Practice. 2015;17(3):201–210.
2. Hanson L, Holmquist-Johnson CL, Cowardin ML. Evaluation of the Raven sUAS to detect and monitor Greater Sage-Grouse leks within the Middle Park population: U.S. Geological Survey Open-File Report. 2014;20p.
3. Barnas A, Newman R, Felege CJ, Corcoran MP, Hervey SD, Stechamann TJ, et al. Evaluating behavioral responses of nesting lesser snow geese to unmanned aircraft surveys. Ecology and Evolution. 2018(2):1328-1338.
4. Barnas AF, Felege CJ, Rockwell RF, Ellis-Felege SN. A pilot(less) study on the use of an unmanned aircraft system for studying polar bears (Ursus maritimus). Polar Biology. 2018;41(5):1055–1062.
5. Linchant J, Lhoest S, Quevauvillers S, Semeki J, Lejeune P, Vermeulen C. WIMUAS: Developing a tool to review wildlife data from various UAS flight plans. ﻿International Archives of the Photogrammetry, Remote Sensing and Spatial Information Sciences - ISPRS Archives. 2015;40:379–384.
6. Linchant J, Lhoest S, Quevauvillers S, Lejeune P, Vermeulen C, Ngabinzeke JS, et al. UAS imagery reveals new survey opportunities for counting hippos. PLOS ONE. 2018;13(11).
7. Lhoest S, Linchant J, Quevauvillers S, Vermeulen C, Lejeune P. How many hippos (Homhip): algorithm for automatic counts of animals with infra-red thermal imagery from UAV. in ISPRS - International Archives of the Photogrammetry, Remote Sensing and Spatial Information Sciences. 2015;355–362.
8. Scobie CA, Hugenholtz CH. Wildlife monitoring with unmanned aerial vehicles: Quantifying distance to auditory detection. Wildlife Society Bulletin. 2016;40(4):781–785.
9. Puliti S, Ørka H, Gobakken T, Næsset E. Inventory of Small Forest Areas Using an Unmanned Aerial System. Remote Sensing. 2015;7(8):9632–9654.
10. Olsoy PJ, Shipley LA, Rachlow JL, Forbey JS, Glenn NF, Burgess MA, Thornton DH. Unmanned aerial systems measure structural habitat features for wildlife across multiple scales. Methods in Ecology and Evolution. 2018;9(3):594–604.
11. Vermeulen C, Lejeune P, Lisein J, Sawadogo P, Bouché P. Unmanned Aerial Survey of Elephants. PLoS ONE. 2013;8(2).
12. Lisein J, Linchant J, Lejeune P, Bouché P, Vermeulen C. Aerial surveys using an Unmanned Aerial System (UAS): comparison of different methods for estimating the surface area of sampling strips Tropical Conservation Science. 2013;6(4):506-520.
13. Szantoi Z, Smith SE, Strona G, Koh LP, Wich SA. Mapping orangutan habitat and agricultural areas using Landsat OLI imagery augmented with unmanned aircraft system aerial photography. International Journal of Remote Sensing. 2017;38(8-10):1–15.
14. Bonnin N, Van Andel A, Kerby J, Piel A, Pintea L, Wich S. Assessment of Chimpanzee Nest Detectability in Drone-Acquired Images. Drones. 2018;2(2):1-17.
15. Van Andel AC, Wich SA, Boesch C, Koh LP, Robbins 506 MM, Kelly J, et al. Locating chimpanzee nests and identifying fruiting trees with an unmanned aerial vehicle. American Journal of Primatology. 2015;77(10):1122–1134.
16. Evans L, Jones T, Pang K, Saimin S, Goossens B. Spatial Ecology of Estuarine Crocodile (Crocodylus porosus) Nesting in a Fragmented Landscape. Sensors. 2016;16(9):1-10.
17. Stark DJ, Vaughan IP, Evans LJ, Kler H, Goossens B. Combining drones and satellite tracking as an effective tool for informing policy change in riparian habitats: a proboscis monkey case study. Remote Sensing in Ecology and Conservation. 2018;4(1):44–52.
18. Lehmann JRK, Prinz T, Ziller SR, Thiele J, Heringer G, Meira-Neto JAA, et al. Open-Source Processing and Analysis of Aerial Imagery Acquired with a Low-Cost Unmanned Aerial System to Support Invasive Plant Management. Frontiers in Environmental Science. 2017;5:1–16.
19. Mulero-Pázmány M, Stolper R, Van Essen LD, Negro JJ, Sassen T. Remotely piloted aircraft systems as a rhinoceros anti-poaching tool in Africa. PLoS ONE. 2014;9(1).
