# Supplementary material for: Steps to build a DIY low-cost fixed-wing drone for biodiversity conservation
Source: PLoS One. 2021 Aug 13;16(8):e0255559. doi: 10.1371/journal.pone.0255559 (PMC8363011; doi:10.1371/journal.pone.0255559)
Supplement: S1 Text — (DOCX) [file pone.0255559.s003.docx]

**# Components List #**

The prices informed in the product links below may vary depending on supply (shipping costs are not included). User registration on some of these websites may be required to access some of the products. Most of the parts required for the drone assemblage were purchased from Aliexpress (<https://best.aliexpress.com/?lan=en>) and Hobbyking (<https://hobbyking.com/pt_pt>) e-commerce.

The prices informed in the following links correspond to 2020 and may have changed since the acquisition date of the components in 2018.

1. **Required DIY Drone parts**
   1. **Airframe - Volantex Ranger 2000** (<https://www.aliexpress.com/item/32914632095.html?spm=a2g0o.productlist.0.0.17366893FBSNPw&algo_pvid=c5ed4bd3-ca5f-4c56-9039-49f9bf6d18a0&algo_expid=c5ed4bd3-ca5f-4c56-9039-49f9bf6d18a0-10&btsid=0ab6f83915827337056162269e05ac&ws_ab_test=searchweb0_0,searchweb201602_,searchweb201603>)

**
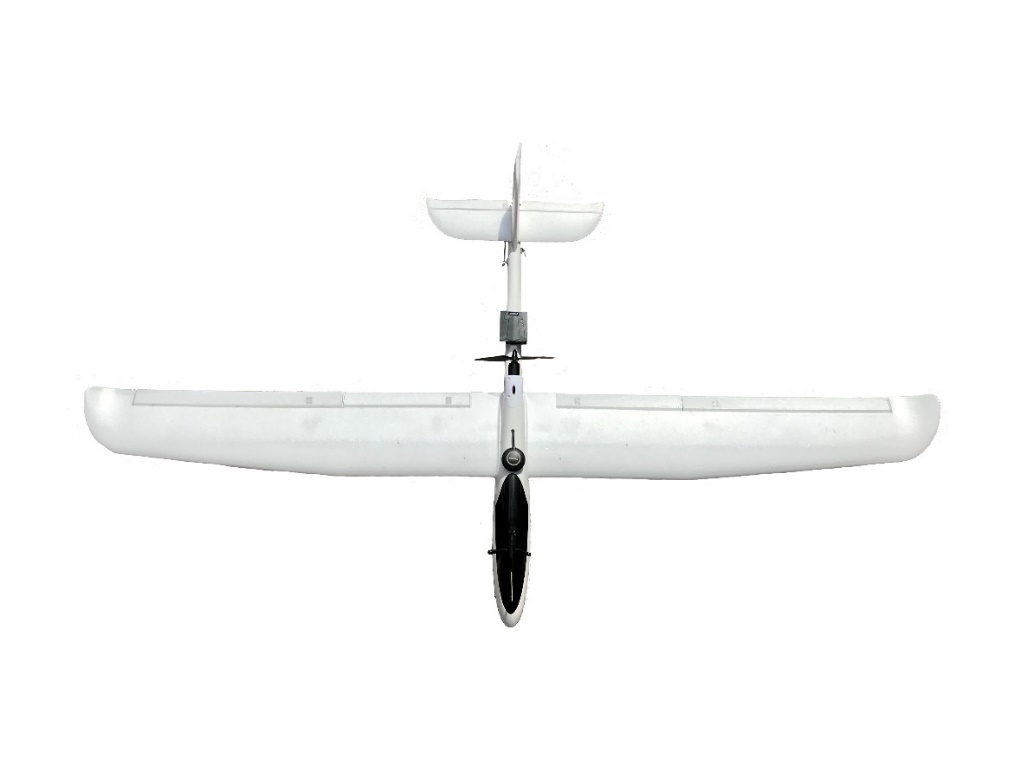
**

The airframe model used was the fixed-wing Ranger 2000 (Volantex-RC, CO., International) with the following features: 2000 mm wingspan; 1100 mm length; 1083 g empty weight. The fuselage is made of hard, flexible plastic, the wings are composed of Expanded polyolefin (EPO) and the control set includes four servos (ailerons, flaps, rudder and elevator). This model is easily launched by hand and recovered by “belly landing”, avoiding the need for complex systems such as catapults or skyhooks. Another aspect considered crucial in the choice of this model was portability: it is modular and can be disassembled into three smaller parts (fuselage; wings and elevator; and rudder) for transportation inside one compact case (110 x 30 x 30 cm3). The airframe model was purchased as the PNP (Plug and Play) version, with all propulsion components pre-installed.

- 1. **Motor - brushless electric engine model 2215 1400 Kv - $0 (included in the airframe price)**

**
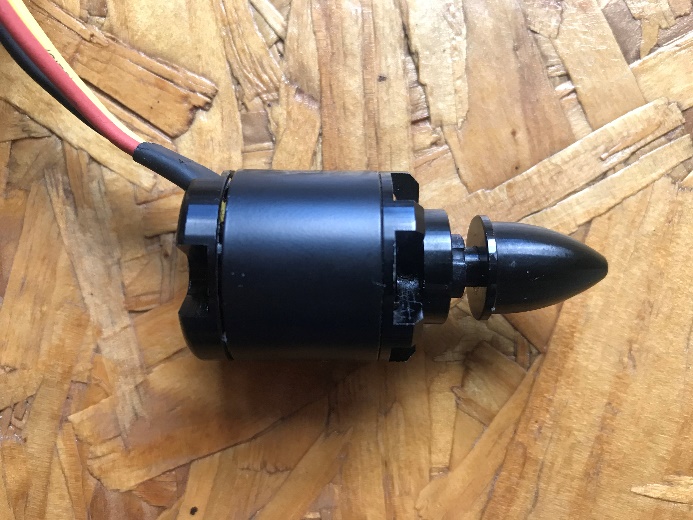
**

- 1. **Servos - 6 X 9g - $0 (included in the airframe price)**

**
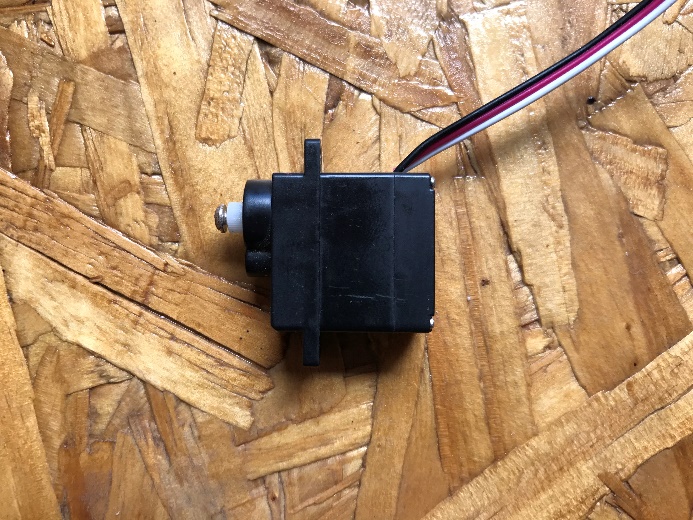
**

- 1. **Propeller - 8 x 4 - $0 (included in the airframe price)**

**
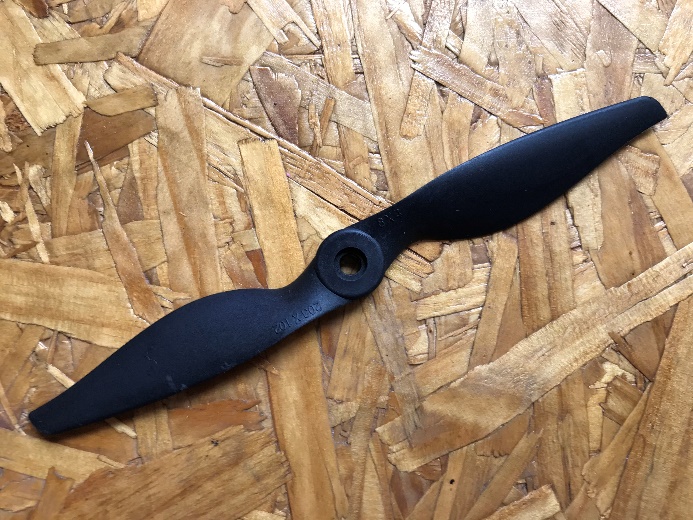
**

- 1. **Electronic Speed Control (ESC) - ESC 30 A 2-4S XT60 Volantex - $0 (included in the airframe price)**

**
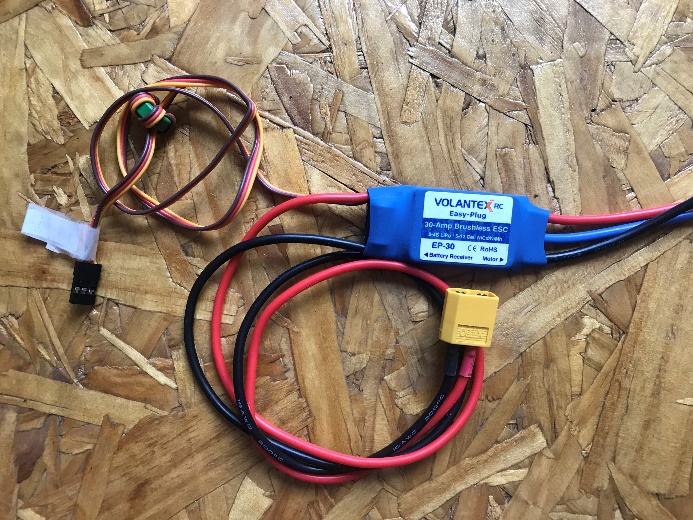
**

To facilitate the DIY development process, we bought the PNP version airframe model. Therefore, the items 2, 3, 4 and 5 come pre-installed on the Volantex Ranger 2000 airframe. The above components were uninstalled from airframe just for didactic purposes in this tutorial, therefore, there is no need to remove them from the airframe.

- 1. **Battery - Turnigy 5000 mAh 4S 14.8 V** (<https://hobbyking.com/pt_pt/turnigy-5000mah-3s-20c-lipo-pack-xt-90.html>)

**
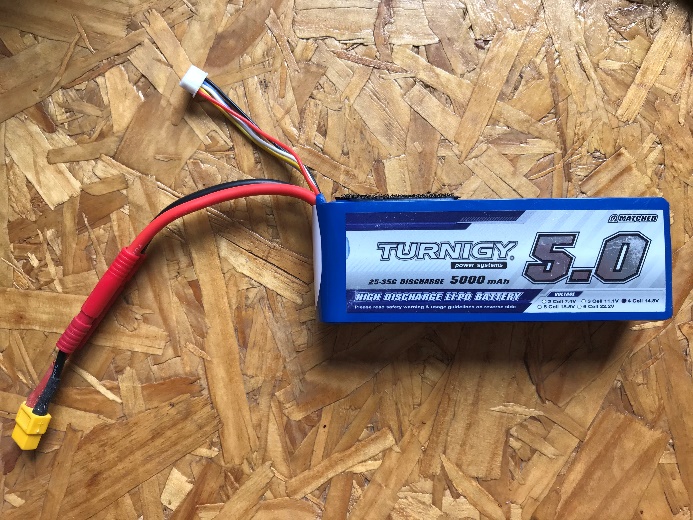
**

According to the airframe manufacturer's recommendations, the battery indicated for the pre-installed motor was a 3S 2200 mAh / 25C / 11.1 V LiPo battery. However, as we aimed to increase flight time, we used a higher capacity 4S 5000 mAh / 25C / 14.8 V LiPo battery. This 4-cell battery provides a voltage operation up to 14.8 V.

- 1. **Charger - SkyRC IMAX B6 Digital** (<https://pt.aliexpress.com/item/32969663849.html?spm=a2g0o.productlist.0.0.6e1e138aH6GZtk&algo_pvid=c8b1fe89-a189-4c48-8252-69dcd4990b59&algo_expid=c8b1fe89-a189-4c48-8252-69dcd4990b59-2&btsid=a7c72df9-d2fb-4175-9674-fab86381c9df&ws_ab_test=searchweb0_0,searchweb201602_5,searchweb201603_55>)

**
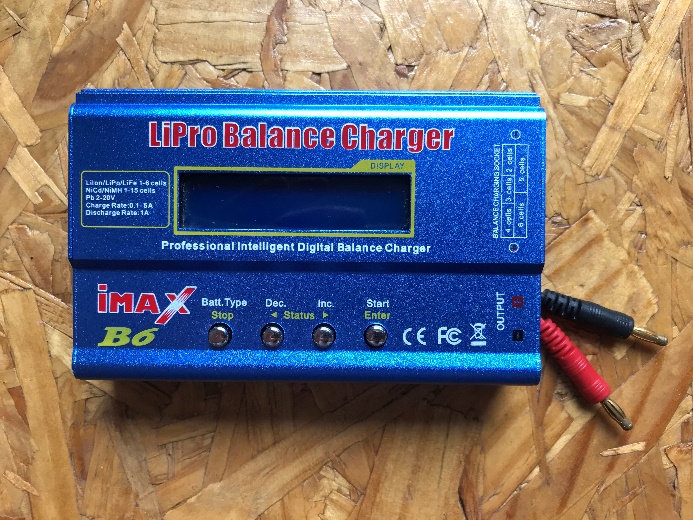
**

This item, although not part of the drone itself, is critical to the operation of the system as it is the power source for charging the drone batteries.

- 1. **Autopilot - mRo Pixhawk 2.4.6** (<https://store.mrobotics.io/Genuine-PixHawk-Flight-Controller-p/mro-pixhawk1-minkit-mr.htm>)

**
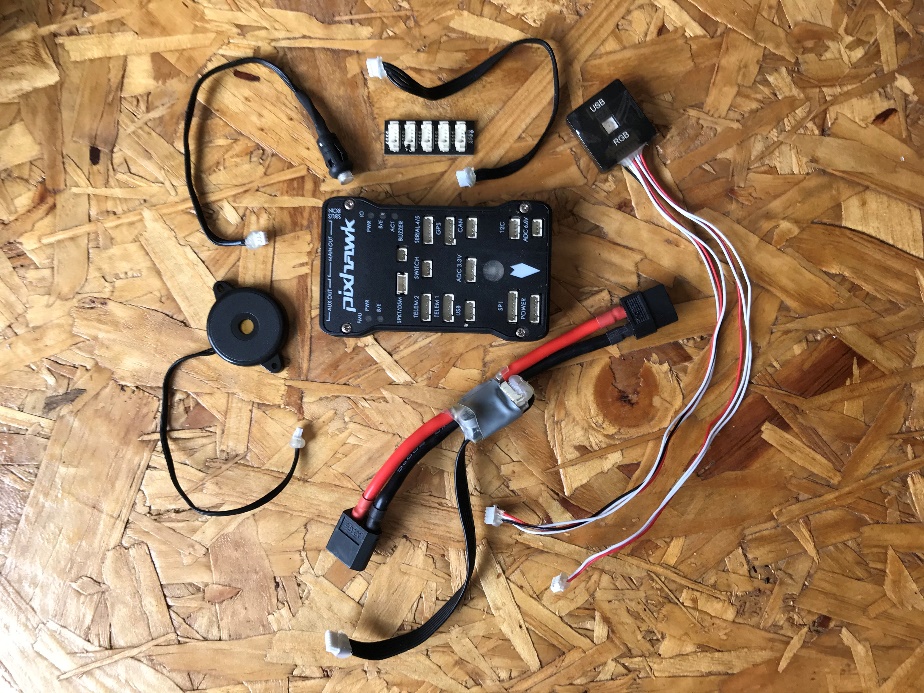
**

mRo Pixhawk 2.4.6 is an advanced autopilot system designed by the PX4 open-hardware project. It features advanced processor and sensor technology from ST Microelectronics® and a NuttX real-time operating system, delivering suitable performance, flexibility, and reliability for controlling any autonomous vehicle.

- 1. **GPS - Ublox NEO-M8N GPS Module** (<https://pt.aliexpress.com/item/32808231236.html?spm=a2g0o.productlist.0.0.7fd330eaMB9C4C&algo_pvid=475af109-a6ae-480b-a70d-b0a9adb0fff5&algo_expid=475af109-a6ae-480b-a70d-b0a9adb0fff5-24&btsid=ee6339e3-d888-417f-ab6d-fd92bf9af1a9&ws_ab_test=searchweb0_0,searchweb201602_5,searchweb201603_55>)

**
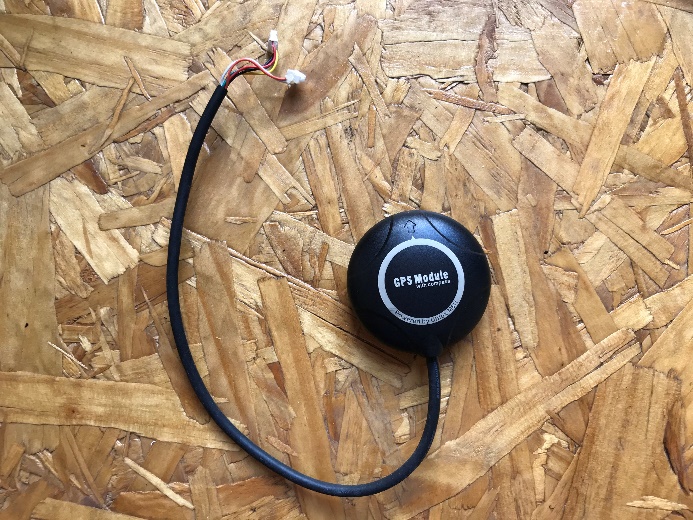
**

Built-in compass, refresh rate up to 10GHz and support GPS+BD+SBAS, or GPS+GLONASS+SBAS

- 1. **Telemetry – 900 RFD 915 MHz** (<https://www.aliexpress.com/item/32810218558.html>)

**
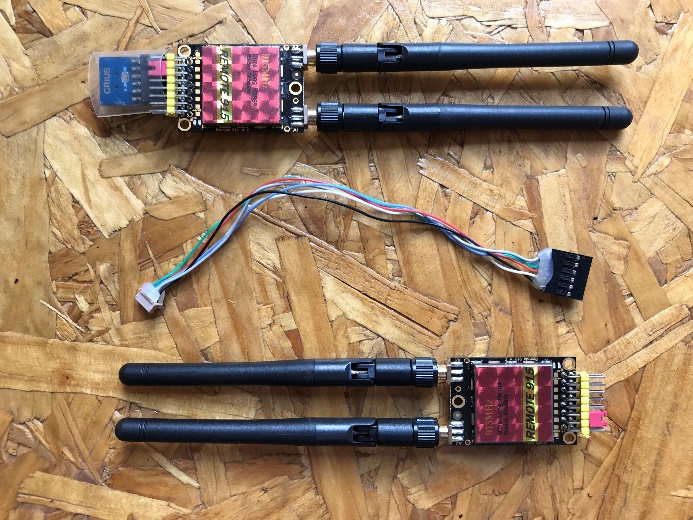
**

Telemetry RFD 900 long-range radio modem at 915 MHz, with an approximate stable range of communication of 40 km. This radio model can receive real-time information from the drone in addition to sending commands from the GCS. Full compatibility with normal "Hope RF" based radio modems as used by all 3DRobotics and compatible Pixhawks and APM's.

- 1. **Sensor - Pitot Tube Airspeedomoter** (<https://www.aliexpress.com/item/32757020070.html?spm=a2g0o.productlist.0.0.143c2d1axwCnWJ&algo_pvid=831c1070-dc64-4d45-9020-2592299acf83&algo_expid=831c1070-dc64-4d45-9020-2592299acf83-17&btsid=d1bc379d-40d6-411b-bf67-6c99bc6cd7e7&ws_ab_test=searchweb0_0,searchweb201602_5,searchweb201603_55>)

**
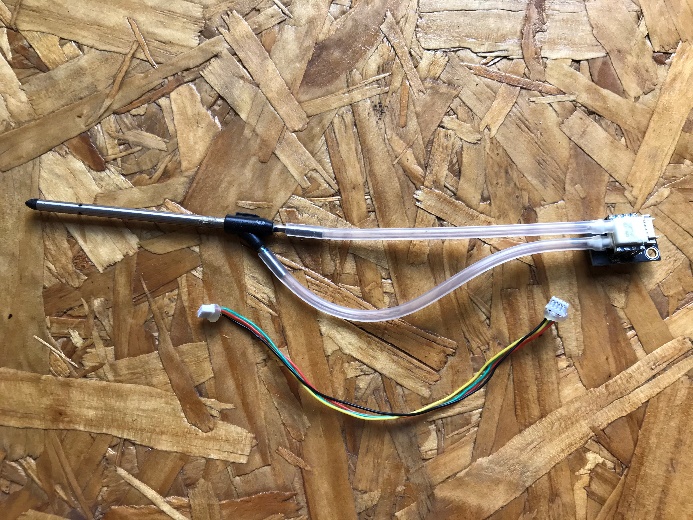
**

This sensor measures differences in air pressure located in a pitot tube in the head of the drone’s airframe and increases the efficiency of autonomous flight. This modified version adopts digital differential pressure sensor, I2C communication, suitable for PIXHAWK and PX4 original firmware.

- 1. **RC - Flysky FS-i6X 2.4 GHz 10CH RC Transmitter** (<https://www.aliexpress.com/item/32885553734.html?spm=a2g0o.productlist.0.0.65f016a7VfiQVA&s=p&algo_pvid=0b63b204-c556-4997-9fc7-91edcdc3c281&algo_expid=0b63b204-c556-4997-9fc7-91edcdc3c281-0&btsid=1c4c7035-8a4a-44ed-84d6-c8bd172b61a4&ws_ab_test=searchweb0_0,searchweb201602_5,searchweb201603_55>)

**
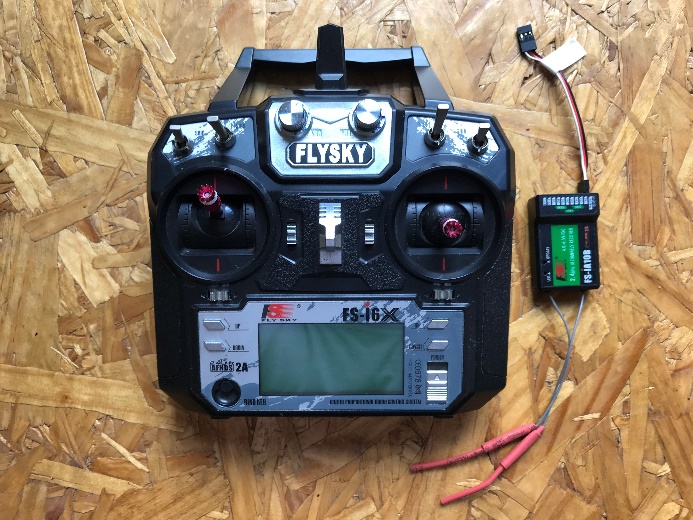
**

The RC model we selected for cost-benefit reasons, was the FlySky 2.4 GHz AFHDS, with an FS-IA10B receiver (10 channels). This bidirectional communication has a multi-channel hopping frequency and omni-directional gain antenna that allow an approximate range of 1 km.

- 1. **Camera - Sony DSC-HX50 (purchased second hand in physical store)**

The RGB compact camera chosen was a Sony model DSC-HX50 with 5184 x 2920 maximum resolution, 20.400.000 effective pixels (px), 1 / 2.3" (~6.16 x 4.62 mm) sensor size and 30x optical zoom. This camera gathers images in the visible spectrum with 1 cm px-^1^ ground sample distance (GSD) resolution, and records Full HD 1080p video.


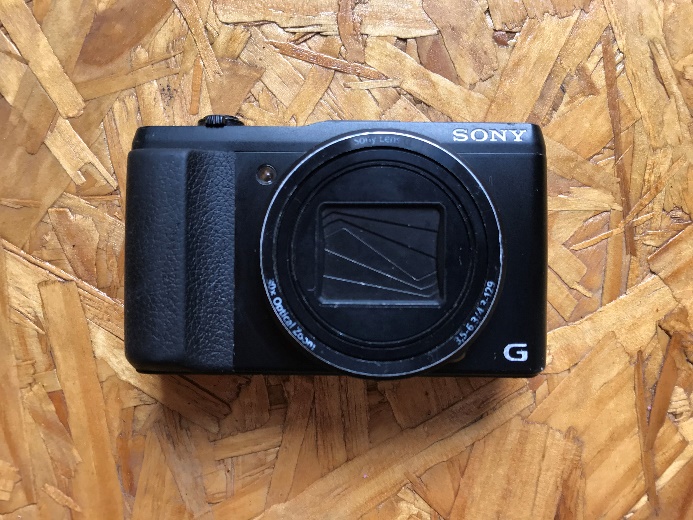


- 1. **Camera trigger – Seagull #MAP2 + cable Sony** (<https://www.seagulluav.com/product/seagull-map2/>)

**
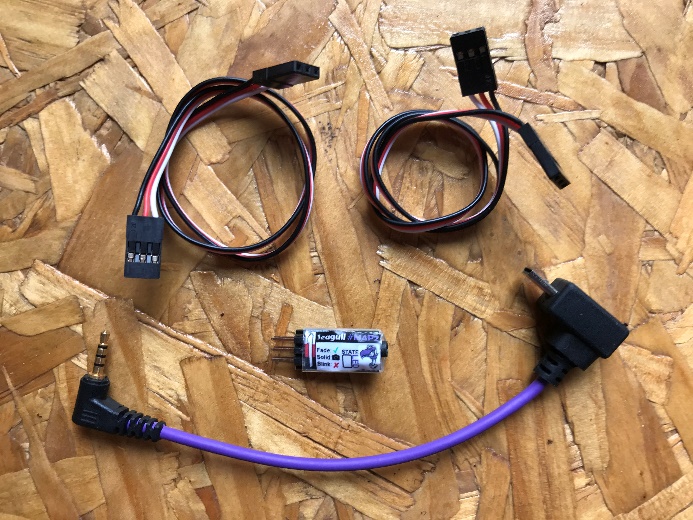
**

Seagull #MAP2 UAV camera trigger to connect the Sony camera with Pixhawk. The #MAP2 allows the on-board camera to be connected with the RC receiver or flight controller and triggers the camera either from a dedicated switch on the RC transmitter or automatically as configured in the flight controller. The price of the kit includes the UAV camera trigger and the Sony S2 (multi) connection cable, also available on the company's website.

- 1. **Accessories – Connectors, cables and tools (purchased different physical store)** for assembly of the DIY drone
