# Supplementary material for: Steps to build a DIY low-cost fixed-wing drone for biodiversity conservation
Source: PLoS One. 2021 Aug 13;16(8):e0255559. doi: 10.1371/journal.pone.0255559 (PMC8363011; doi:10.1371/journal.pone.0255559)

**# Building DIY Drone - assembly instructions and connections #**

With all components (S1. Components List) ready we start the assembly and connections process.

1. Wiring Instructions
   1. Basic Components

As the airframe was purchased in the PNP (Plug-and-Play) version and the components: motor, servos and ESC are already pre-installed on the airframe, there is no need for modifications. The electric motor is connected to the ESC using the three motor wires. The cables can be connected in any order. Once everything is setted-up, the propeller rotation must be checked. Swap the position of the two cables to change its rotation direction. To know how to make the primary connections and perform the check we recommend following the instructions provided in:

<https://www.youtube.com/watch?v=j61Q3e8AFR4&feature=em-share_video_user>

- 1. Pixhawk

Pixhawk Overview: <https://ardupilot.org/plane/docs/common-pixhawk-overview.html>

1. 12C Splitter (required)

The Pixhawk autopilot kit comes with an **12C Splitter** that serves to expand the Pixhawk 12C port capability to allow up to four additional peripherals. This component will serve to connect the compass (integrated to GPS) modules and digital airspeed sensor. We recommend pasting the 12C Splitter on the Pixhawk as shown below and then connect the 12C Splitter to the Pixhawk 12C port using the 4-wire cable.


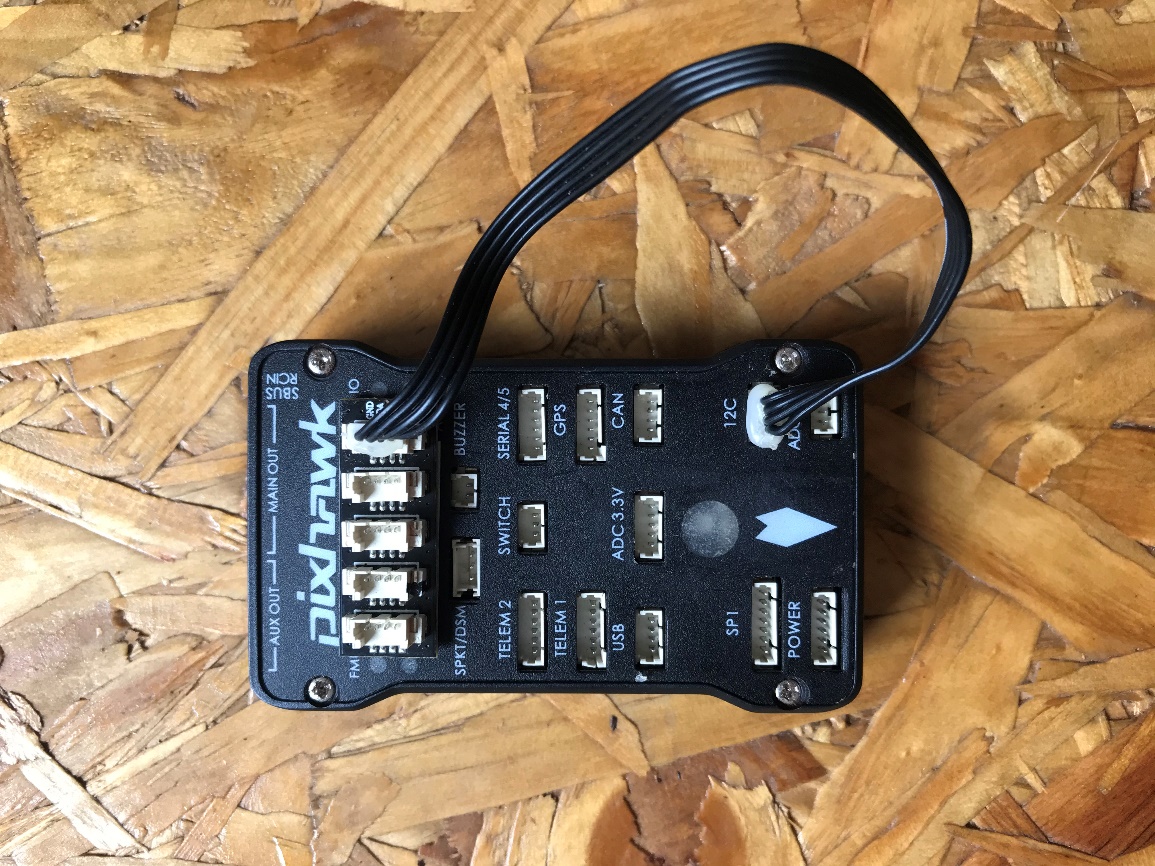


**12C Splitter**

1. Buzzer and safety switch (required)

Next, we connect the **Buzzer** and **Safety Switch** to the Pixhawk. These are mandatory components for Pixhawk functioning. The Buzzer serves to emit audible signals that can indicate different situations and the Safety Switch serves to arm (when armed, motors are powered and propellers can turn) or to disarm the vehicle.


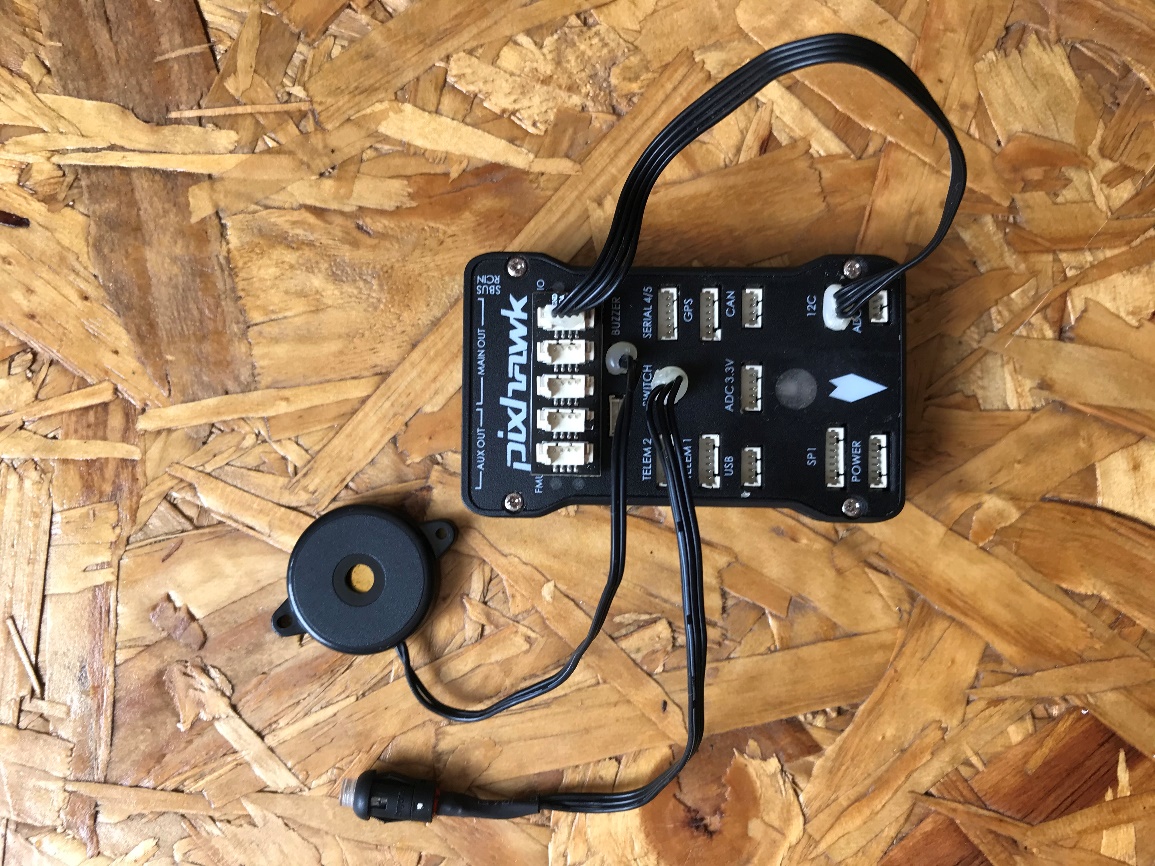


**Safety Switch**

**Buzzer**

1. Power module (required)

Connect the **Power module** to Pixhawk Power port using the 6-wire cable. One part of the power module will be connected to the battery and the other to the ESC (instructions in the next section).


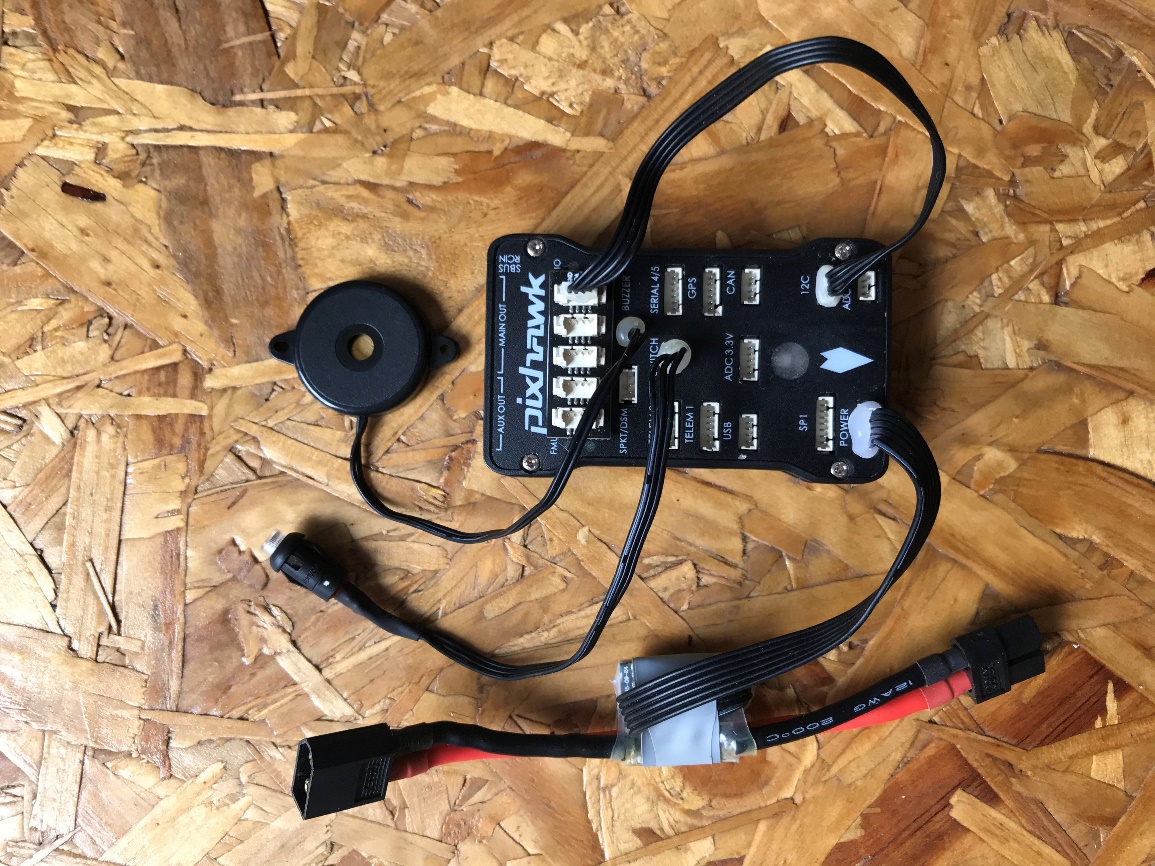


**Power module**

1. USB and LED extension

This extension is used for easily USB connection to the Pixhawk board without disassembling from the airframe. The **USB and LED extension** are connected to the Pixhawk USB port using the 3-wire cable and to the one of the 12C Splitter ports using the 4-wire cable. Check labeling on the extension module.


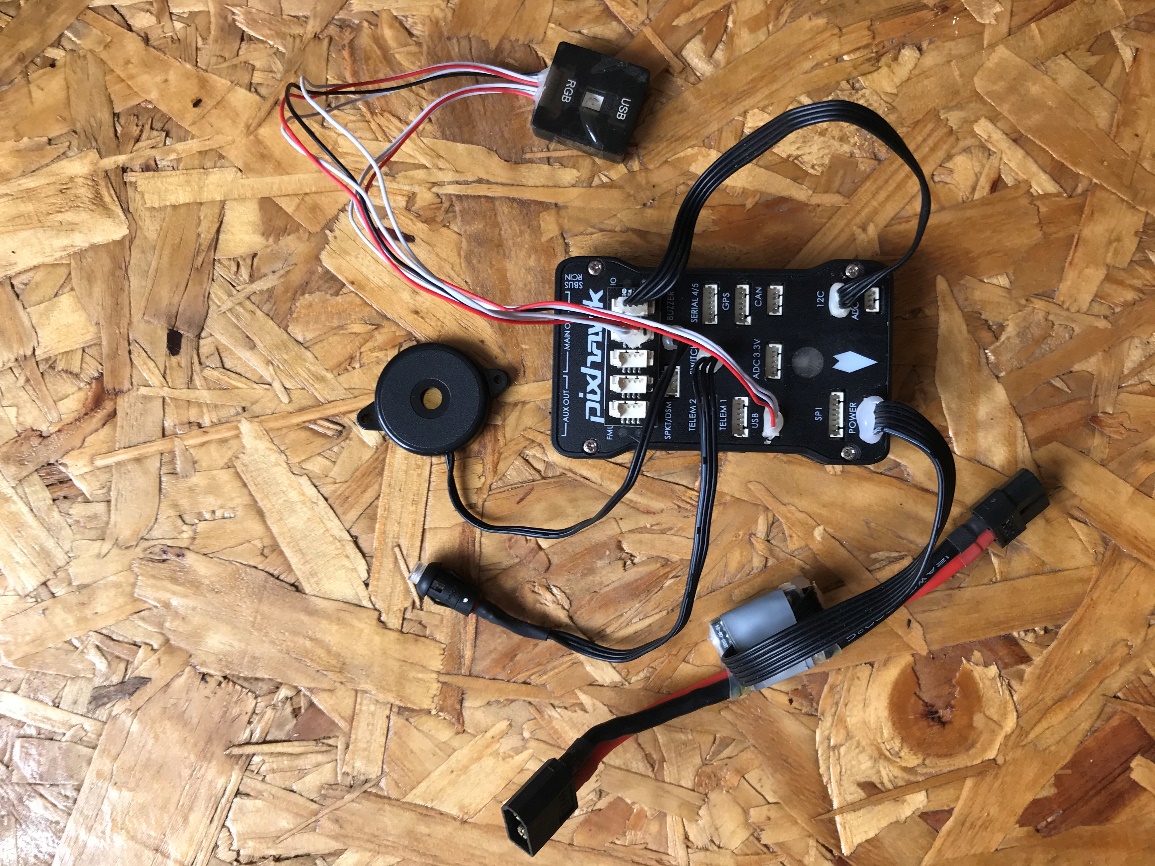


**USB-LED extension**

1. GPS and Compass module

The **GPS module** is connected to the GPS port in the Pixhawk using the 4-wire cable, and the compass to the one of the 12C Splitter ports using the 2-wire cable.


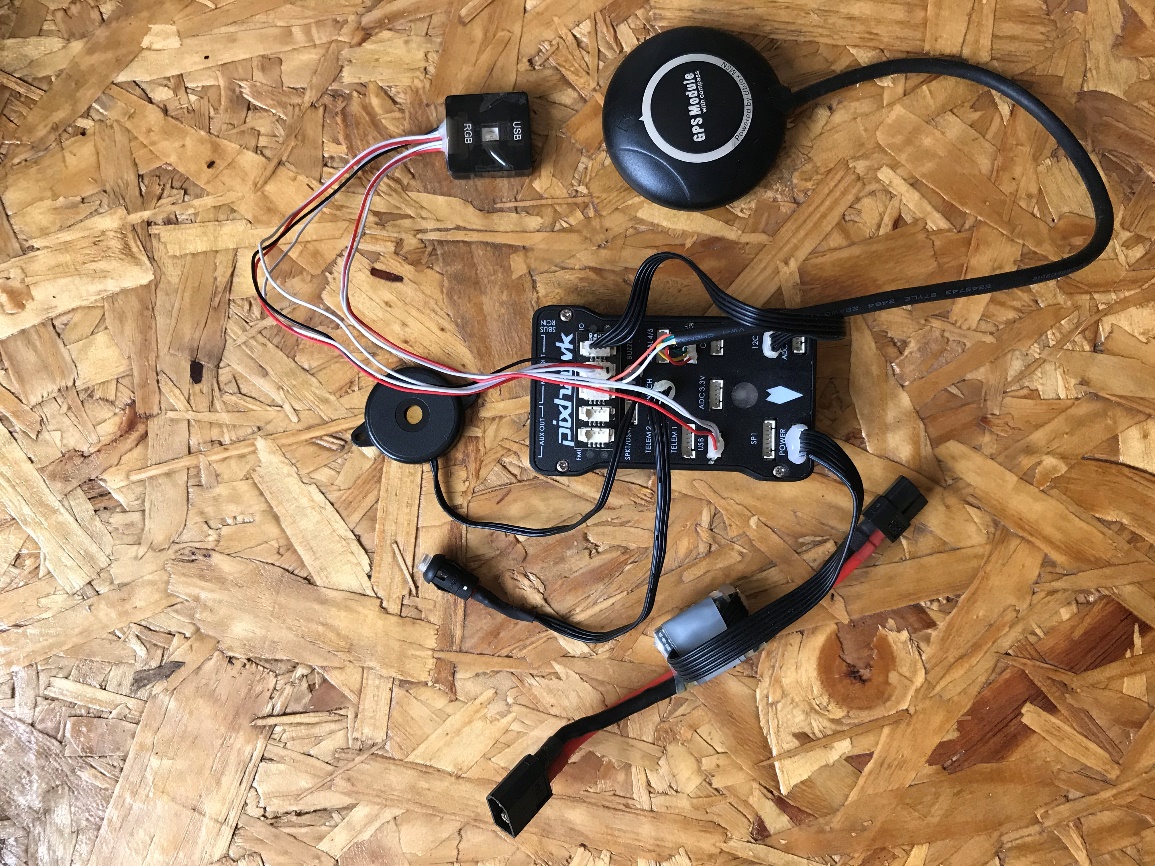


**GPS module**

1. Telemetry (900 RFD 915 MHz)

Connect the long-range radio modem **Telemetry 900 RFD 915 MHz module** to the TELEM 1 using the 6-wire cable. The other Telemetry RFD 915 MHz module is connected to the ground station computer using the USB cable.


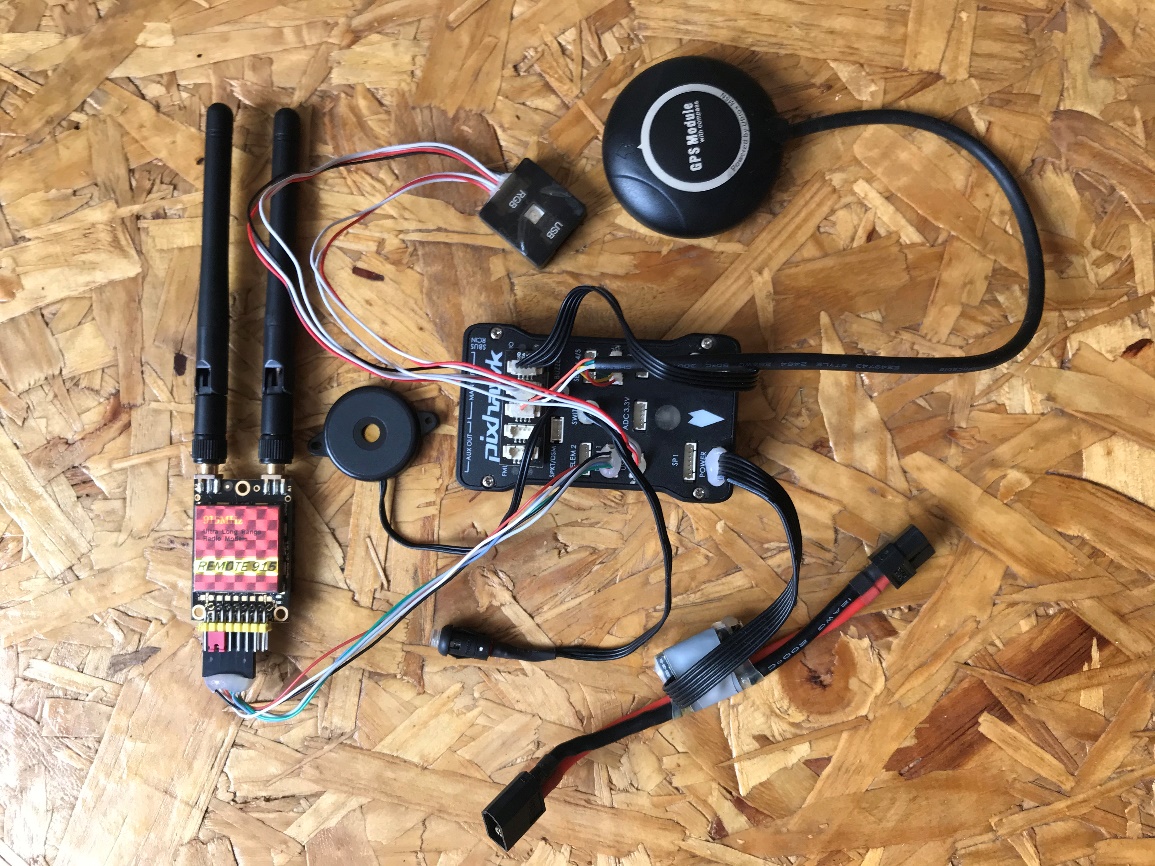


**Telemetry 900 RFD**

1. Airspeedometer

Connect the **Airspeed sensor** to the one of the 12C Splitter ports using the 4-wire cable. The other cable connection is connected to the airspeed sensor board. Using the rubber tubing, connect the longer extension on the pitot tube to the cone that protrudes from the top of the airspeed sensor board (off the off-white, square section protruding off the top of the board), and connect the shorter extension on the pitot tube to the cone protruding from the base of the board.


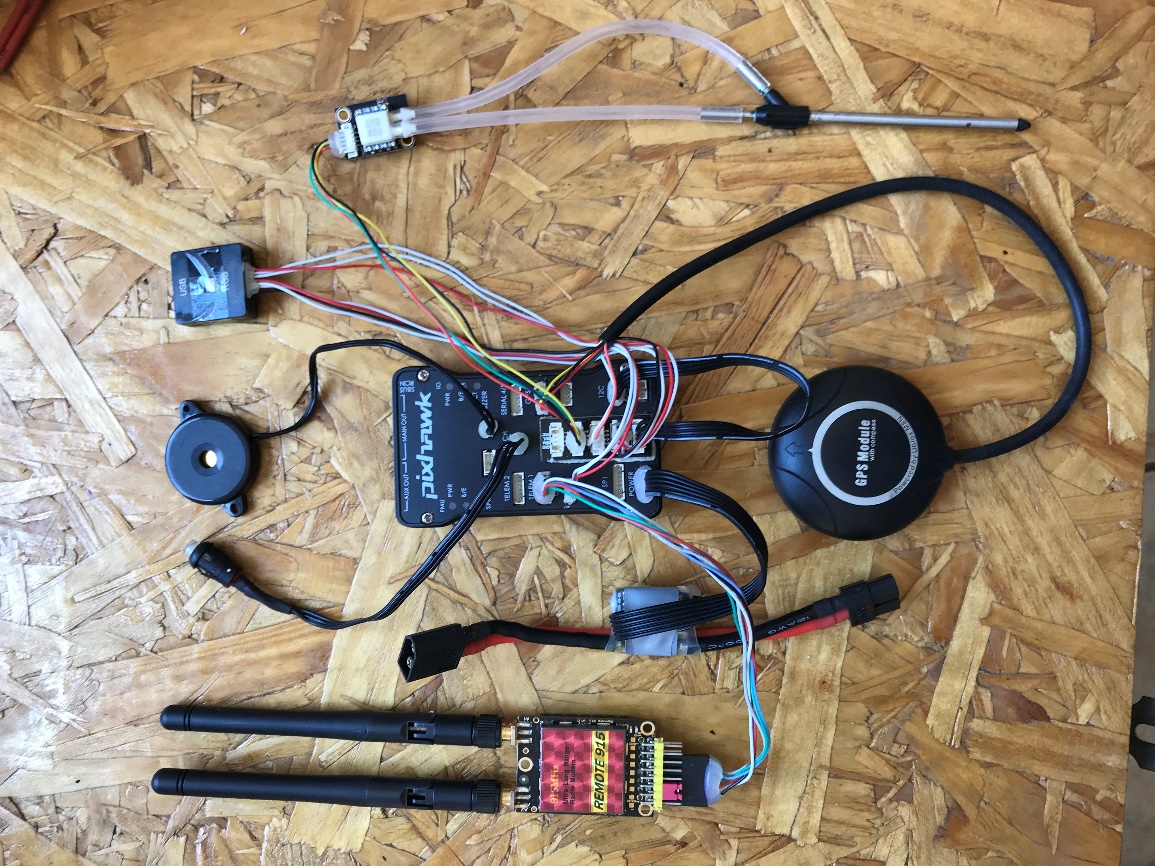


**Airspeed sensor**

1. RC Receiver 2.4 GHz

Connect the **RC receiver** from the PPM/CH1 port on the receiver to the RC port in the Pixhawk board using the 3-wire cable (servo cable). Check cable polarity (- black, + red, signal white). RC transmitter setting must be changed to allow PPM communication (see RC manual).


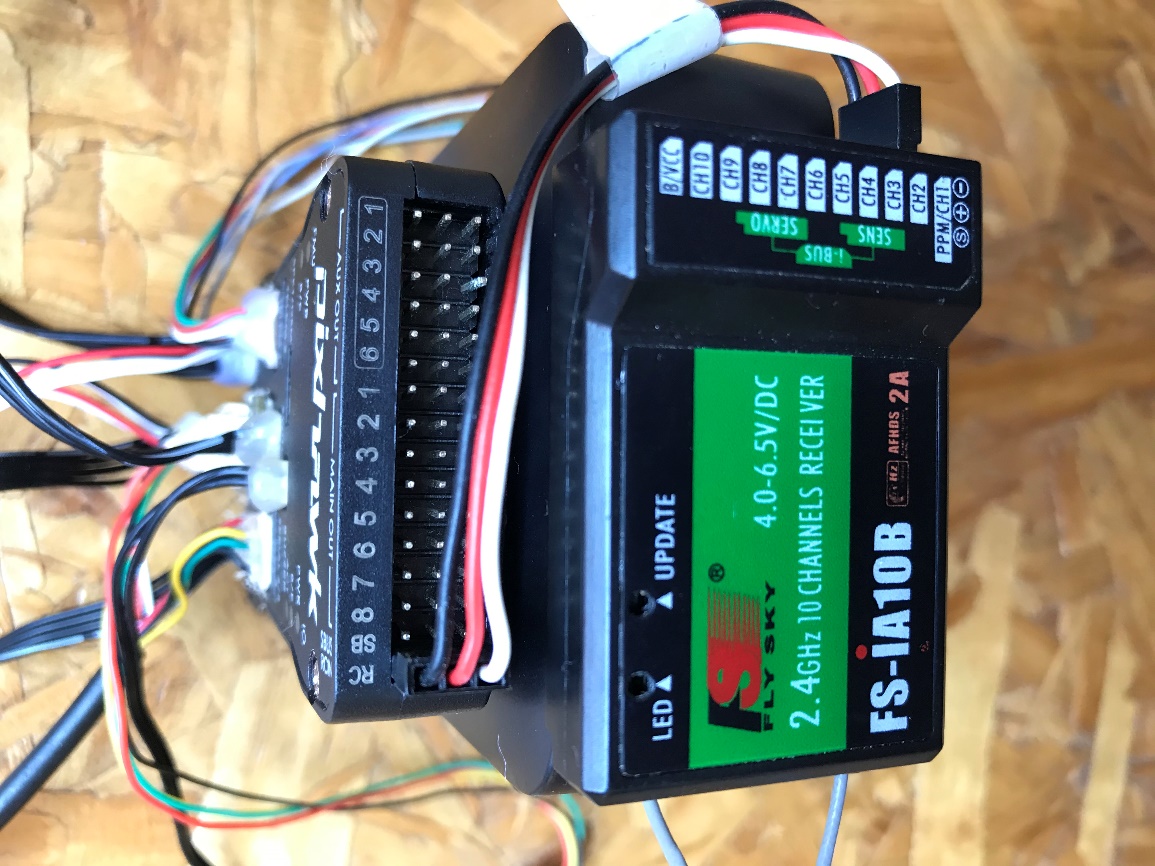


**RC receiver**

1. Camera Trigger

The **Camera trigger** is designed to connect the drone mounted camera to the Pixhawk Flight Controller board and automatically trigger the camera according to the flight mission plan for image mapping or to turn on and off the camera. Two 3-wire cables (servo cable) come out from the camera trigger board and are connected to the AUX OUT ports 5 and 6 Pixhawk board, in this case, following the polarity markings on Seagull #MAP2. The connection in the Pixhawk board ports can be changed according to the settings in the flight configuration software. For more information on Seagul # MAP2 connections and configurations check the online documentation (See Item 5 in S5 Text)


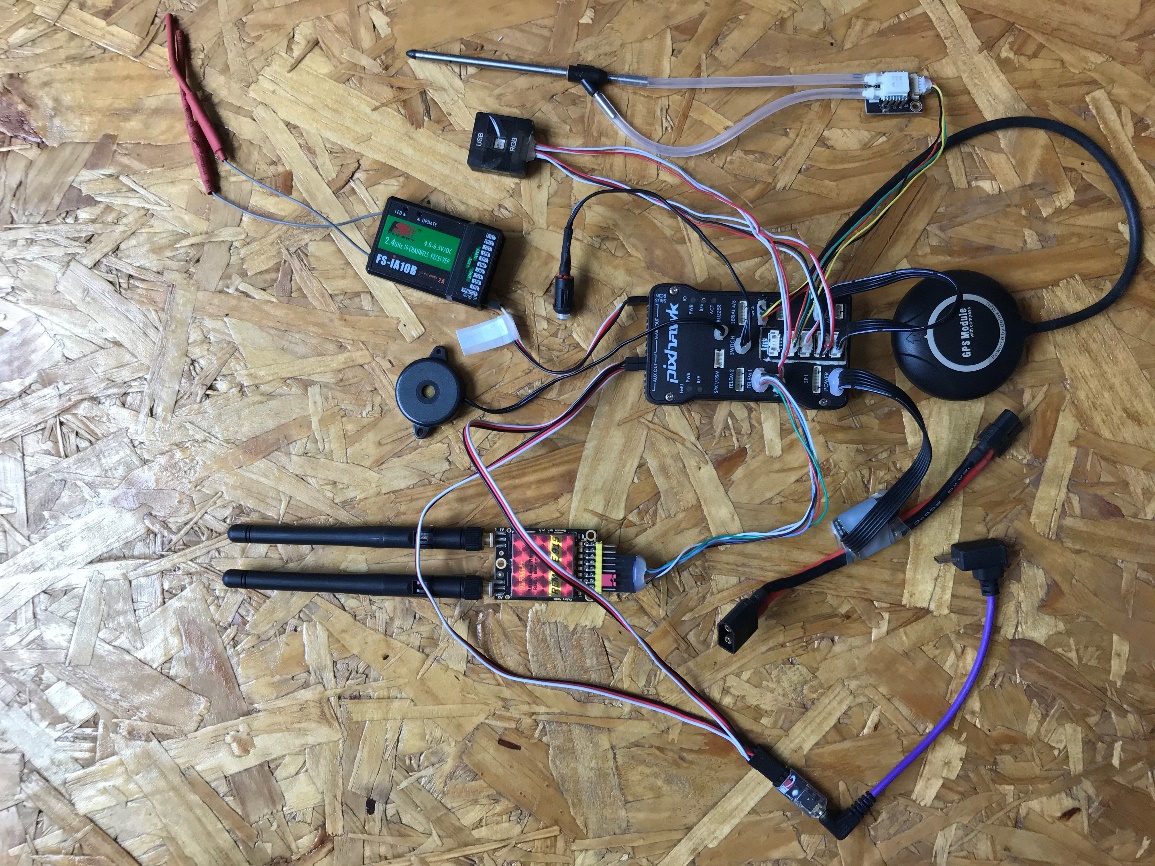


**Camera trigger**

1. Camera

The **Camera** is connected to the Camera trigger by the **micro-USB cable Sony S2**. Different cameras require different connection cables.


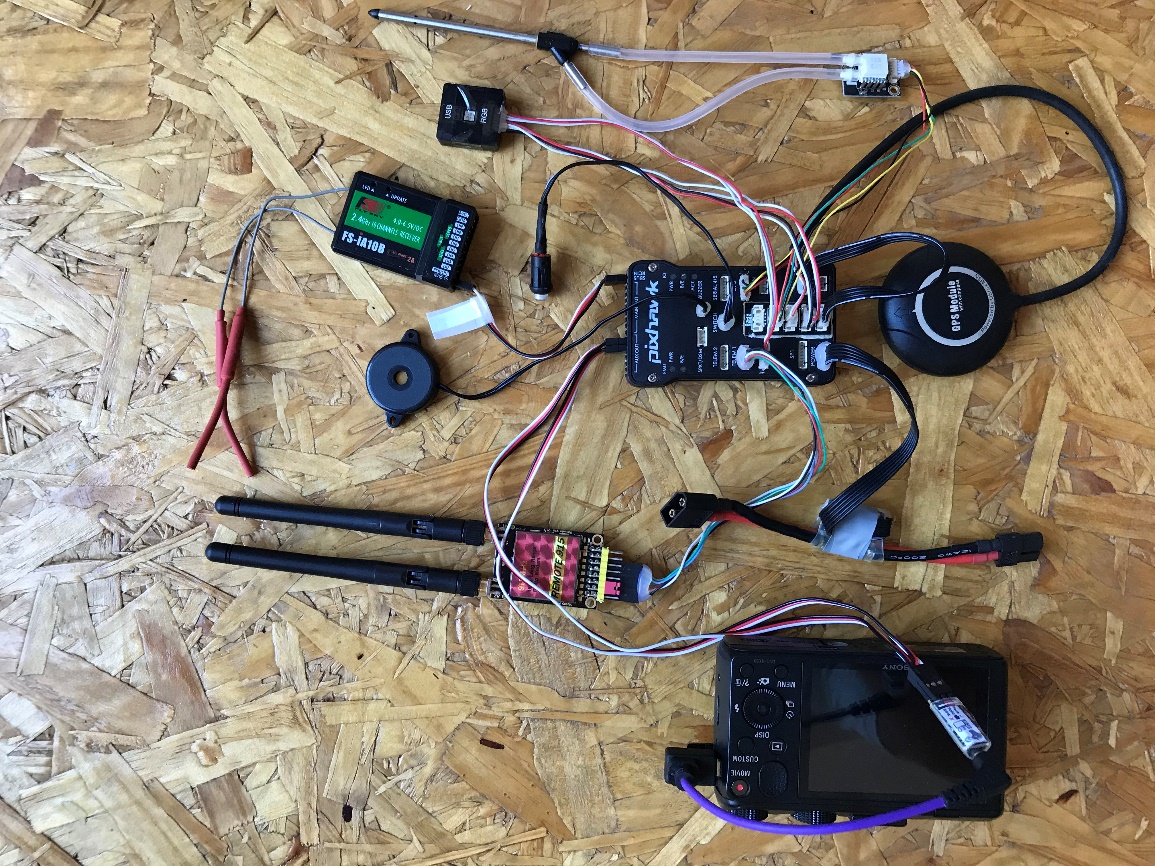


**Camera and cable trigger**

1. MAIN OUT Connections

Motor, Servos and ESC are preinstalled in the Volantex Ranger 2000 airframe. In this case, we only need to connect the servos cables on the Pixhawk board MAIN OUT ports according to the table below:

| **Cables** | **Pixhawk MAIN OUT ports** |
| --- | --- |
| Ailerons* | 1 |
| Elevator | 2 |
| Throttle | 3 |
| Rudder | 4 |
| Flaps* | 5 |

* Aileron/Flaps use two servos, they should be connect together using the “Y” cable provided. The airframe usually comes with this already assembled, but check before connection.


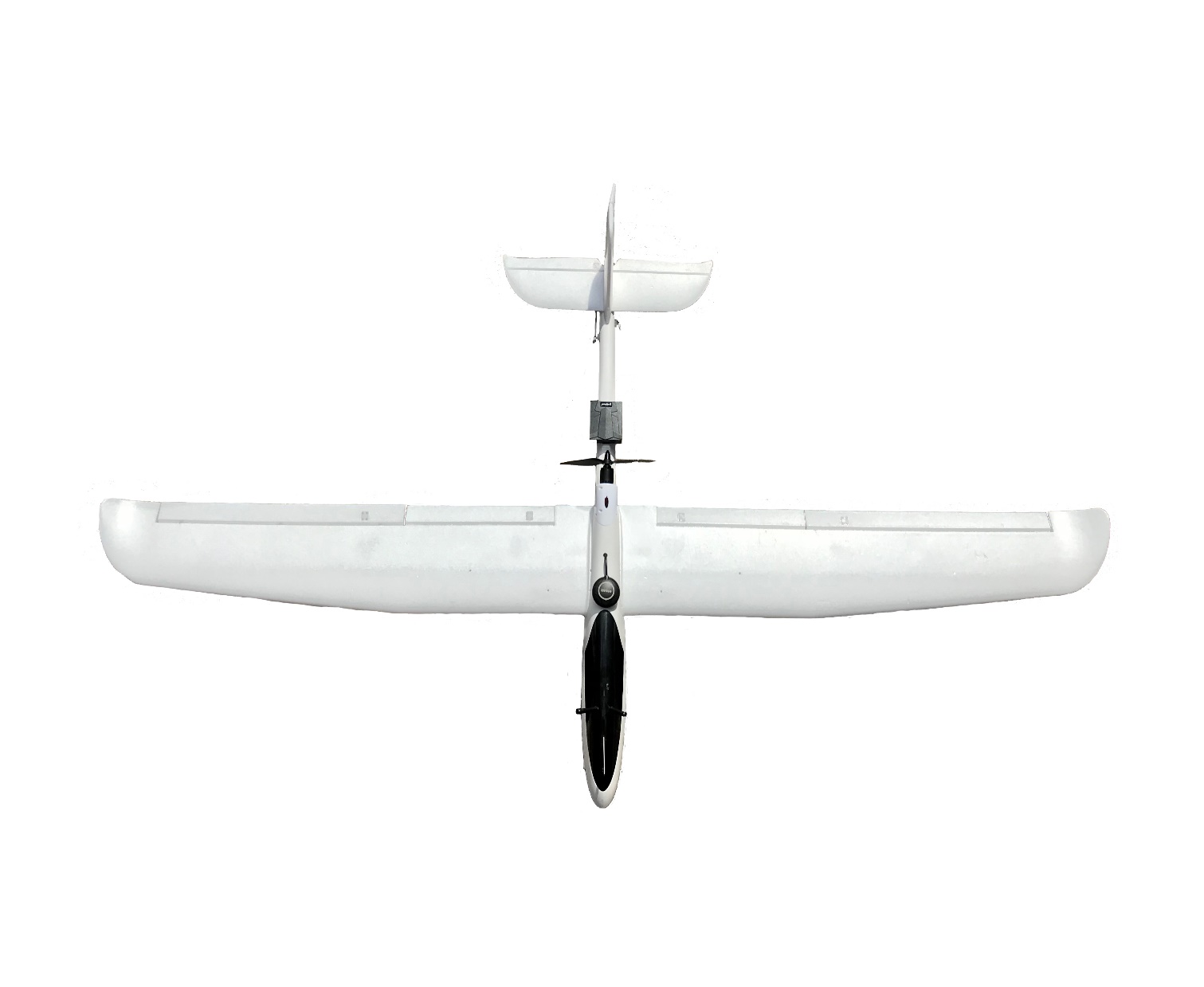


Throttle

Rudder

Elevator

Flaps

Ailerons


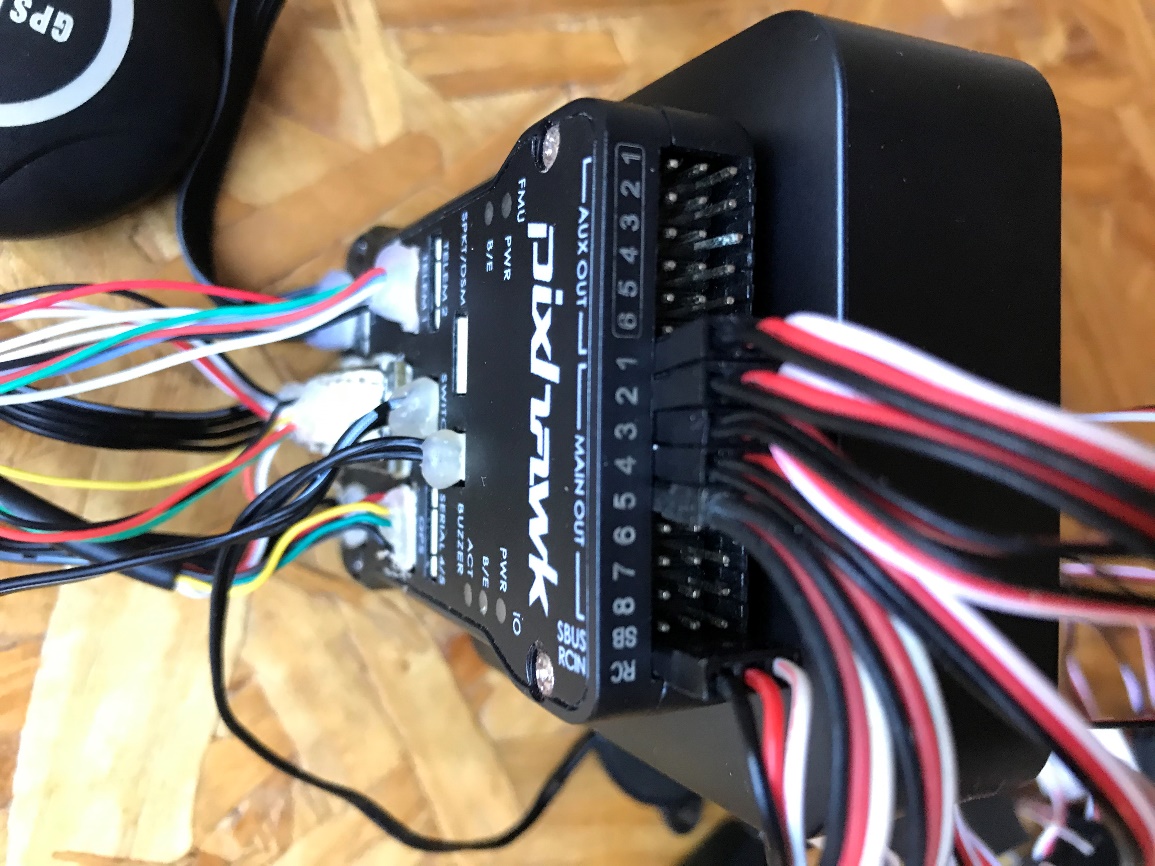


Check cable polarity (- black, + red, signal white). The black wires up, next to the numbers/letters.

1. Airframe modifications
   1. Battery and cables position


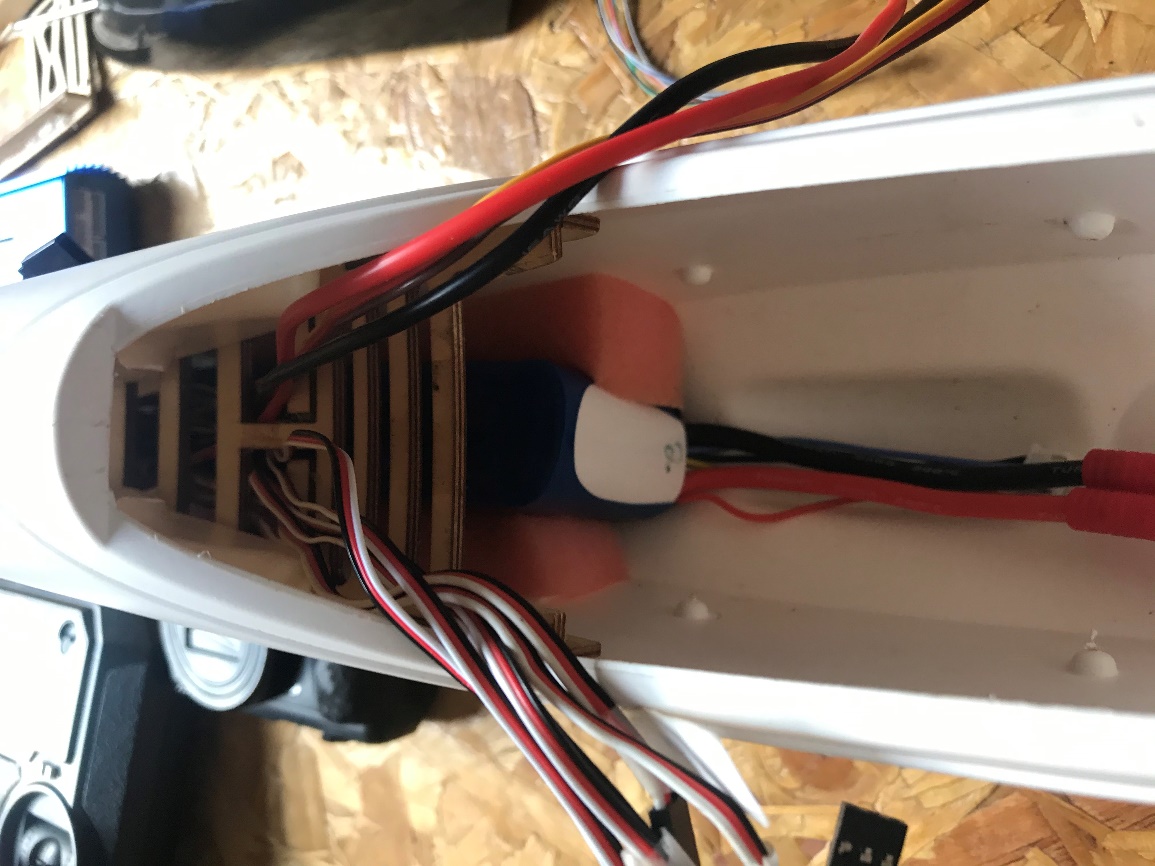


Because the 5000 MAh battery is the heaviest component of the Asa-Branca-I, it is very important that its position is balanced within the airframe. The battery was positioned in the middle of the airframe, as shown above and balanced by two foams attached to the internal sides of the airframe. In addition, we sticked a Velcro to the bottom of the battery and airframe to reinforce the fixation and prevent its internal movement that could compromise the balance of the drone. Part of the internal wooden structure was removed to improve the passage of cables and expand the internal space, as shown above.

- 1. Camera position

First, we removed the internal wooden structure that came with the airframe, then we drilled a hole in the bottom of the airframe front with enough diameter that it was possible to pass the lens of the Sony model DSC-HX50, as shown below. Between the camera and the inner bottom of the airframe, we attach foam to minimize vibration in the camera.


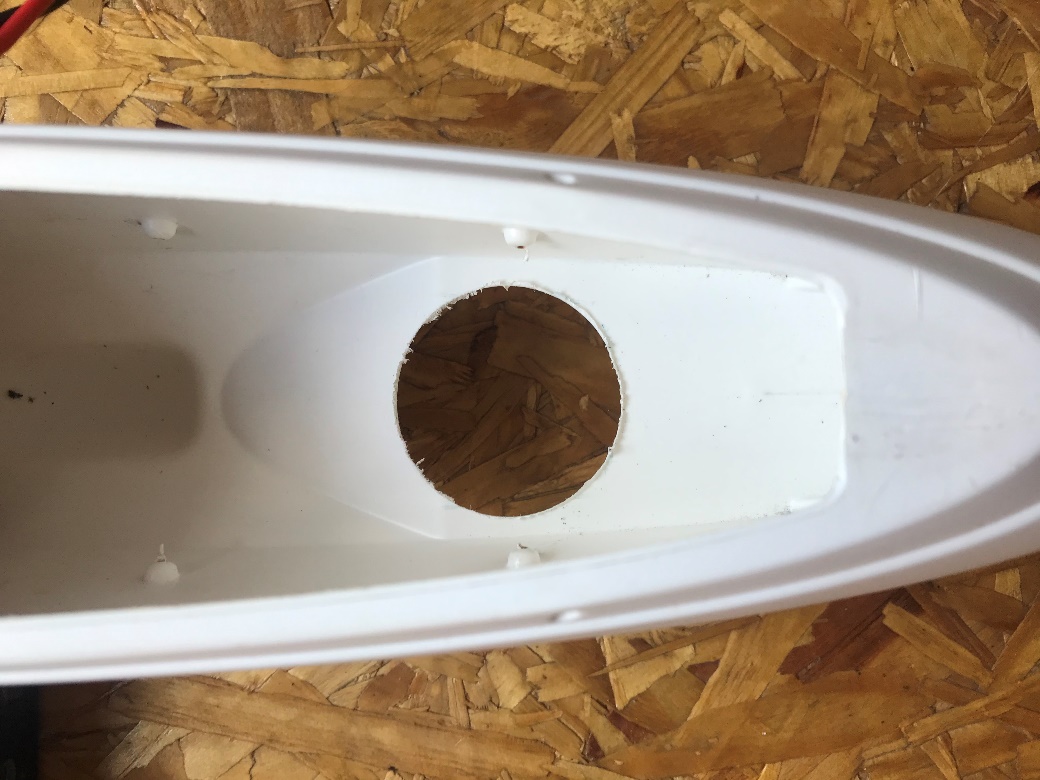


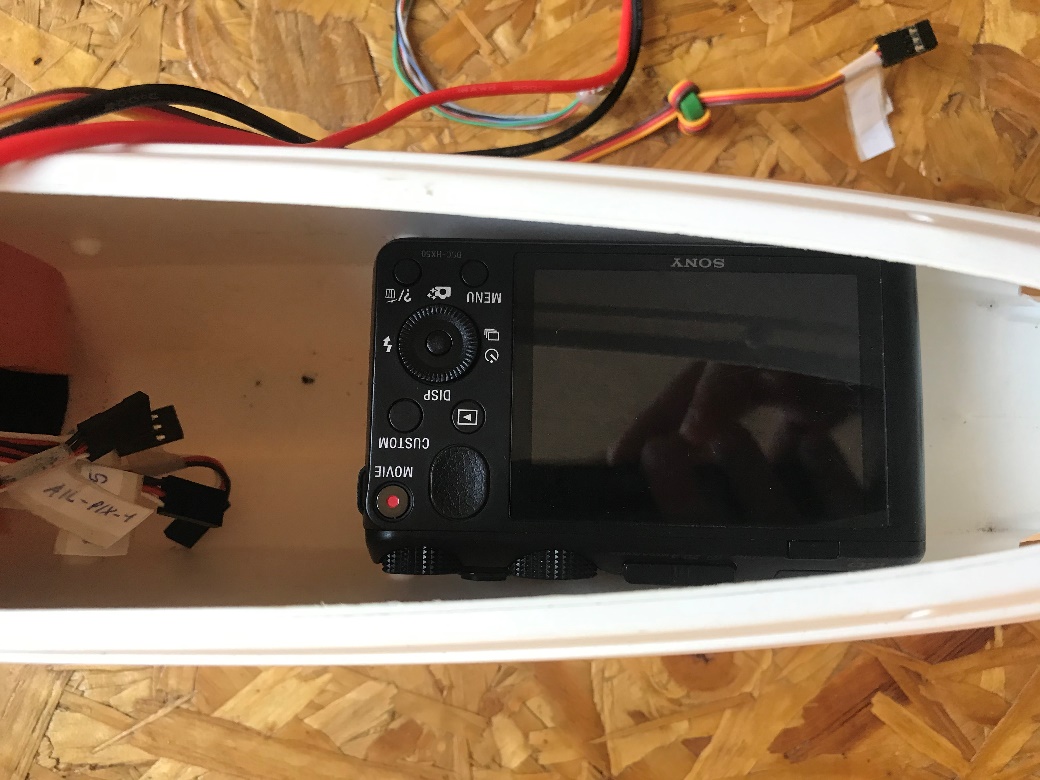


- 1. Pixhawk position

In order to facilitate maintenance tasks and/or allow for modifications, we positioned the Pixhawk at the bottom of the inner cover of the airframe head, as shown in the figures below. As in this position the Pixhawk is upside down, it will be necessary to perform an inversion configuration on the flight controller (see item 5 S3 Text).

For this adaptation it was necessary to cut part of the inner cover of the airframe head. To decrease the vibration on the Pixhawk, we fixed 4 rubber vibration dampers balls to the bottom of the Pixhawk, in addition to the foam between the inner cover of the airframe head and the Pixhawk.


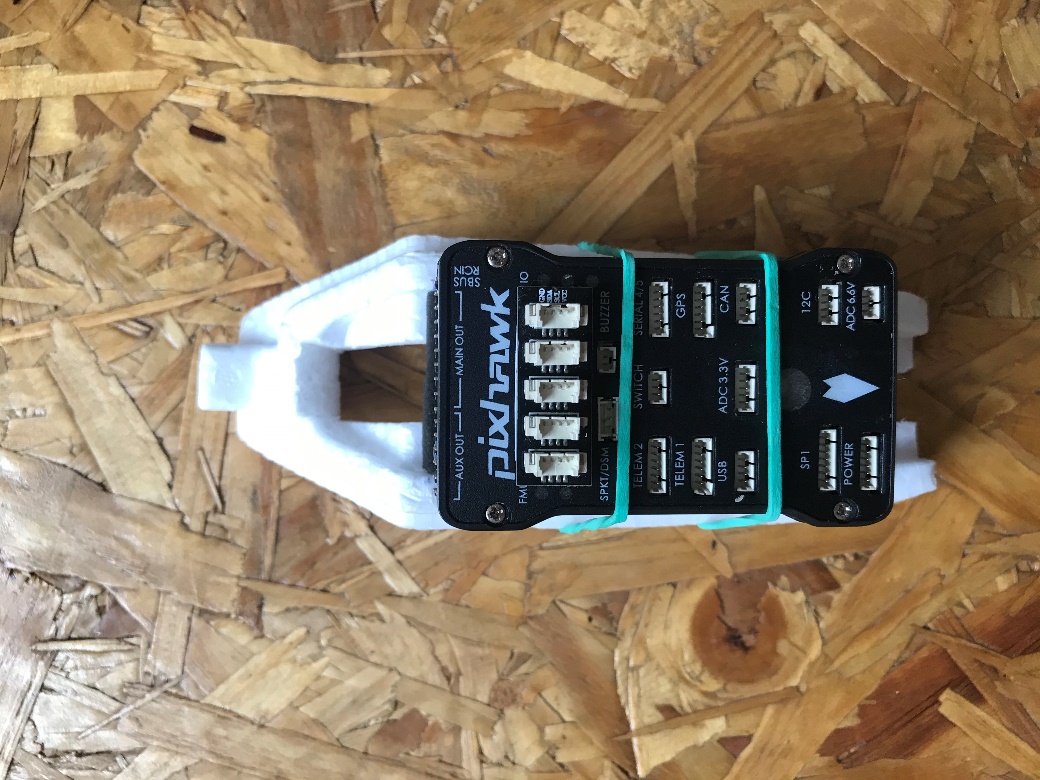


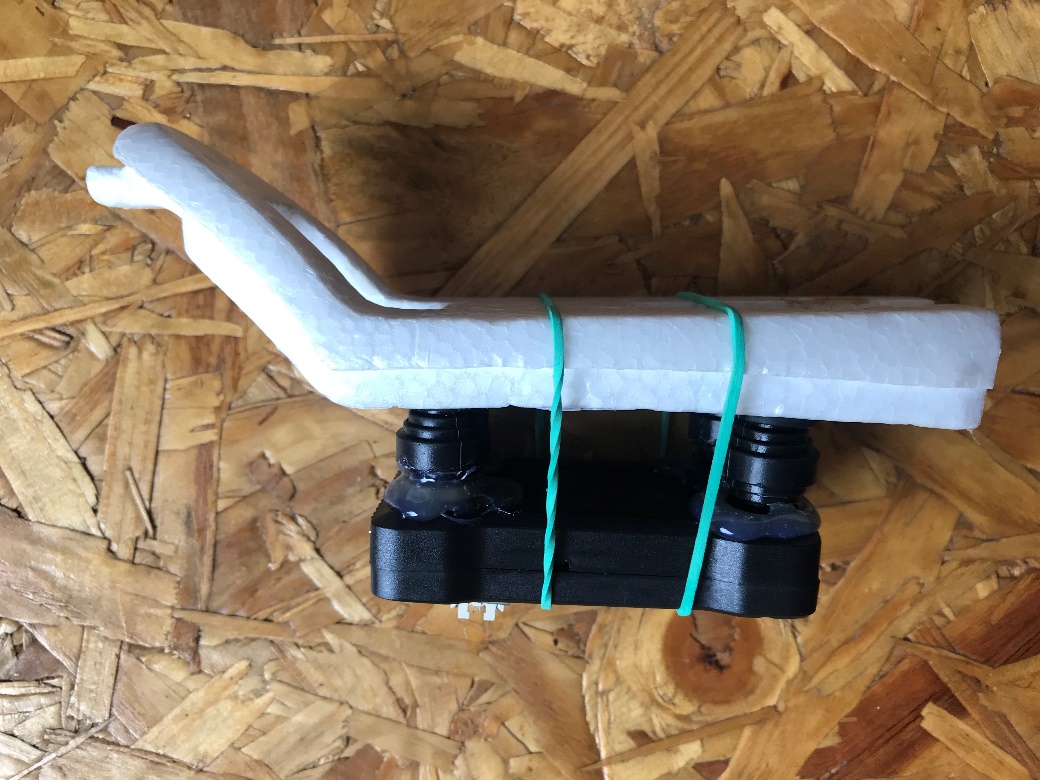


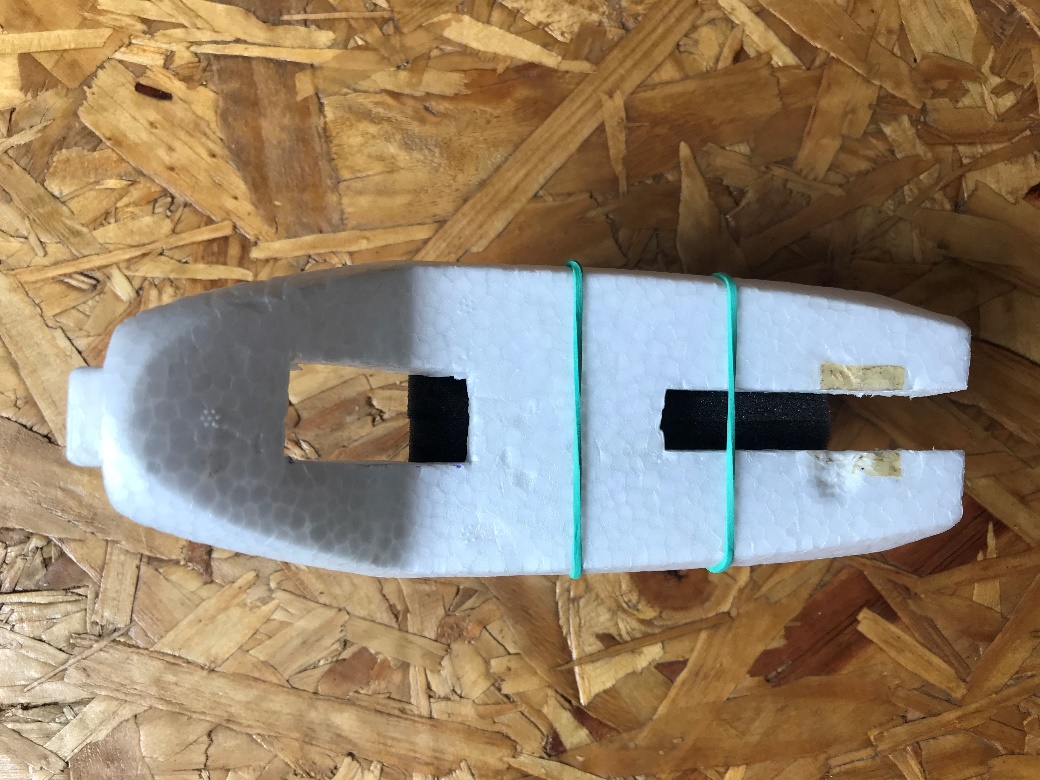


- 1. GPS position

The GPS module was positioned above the Airframe in order to avoid any signal blockage or interference if it was inside the airframe. For this, it was necessary to open a hole in the upper part of the airframe to pass the cables of the GPS module.


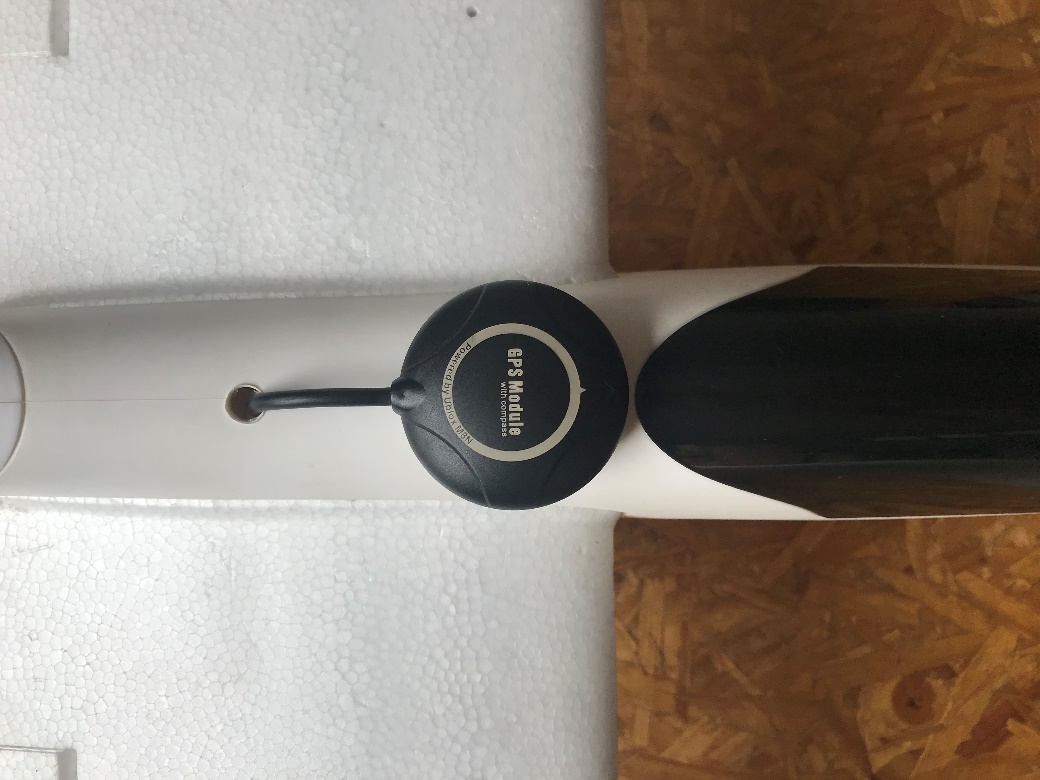


- 1. Telemetry and Airspeed sensor position

At the end of the outer cover of the drone's head, we drill a hole and insert the airspeed sensor tube, and fix it with hot glue on the inside. We also drilled two holes at the top of the outer cover for the passage of the two telemetry antennas as shown in the images below. The airspeed sensor board is between the cover and the telemetry board.


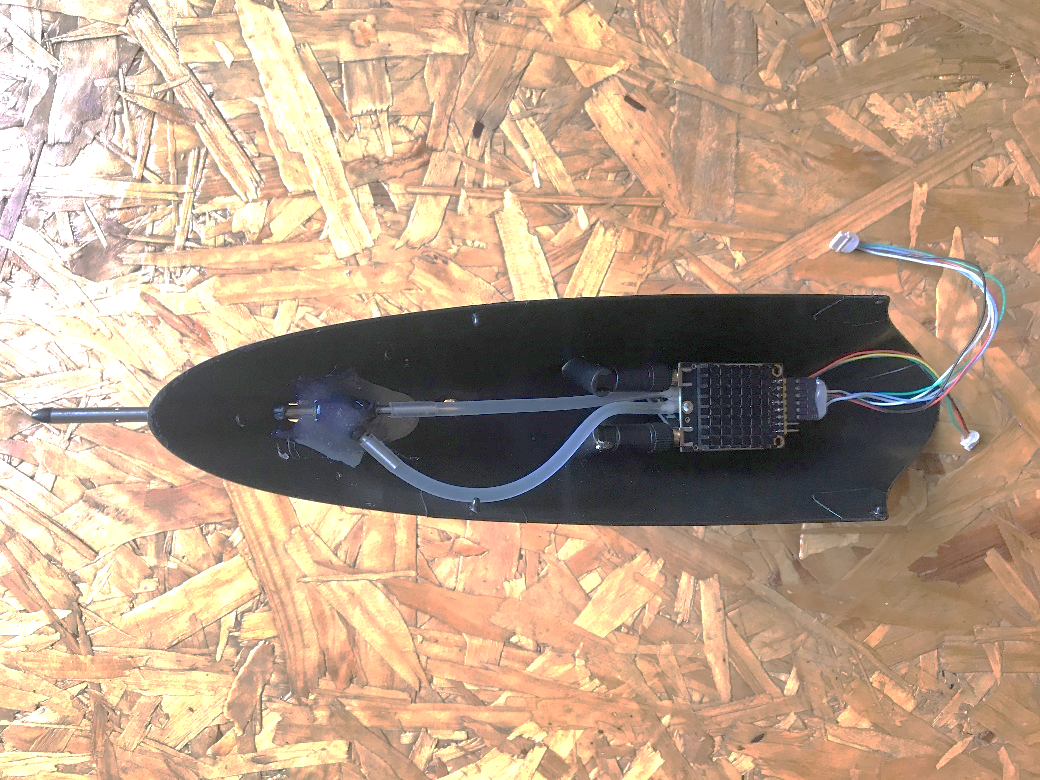


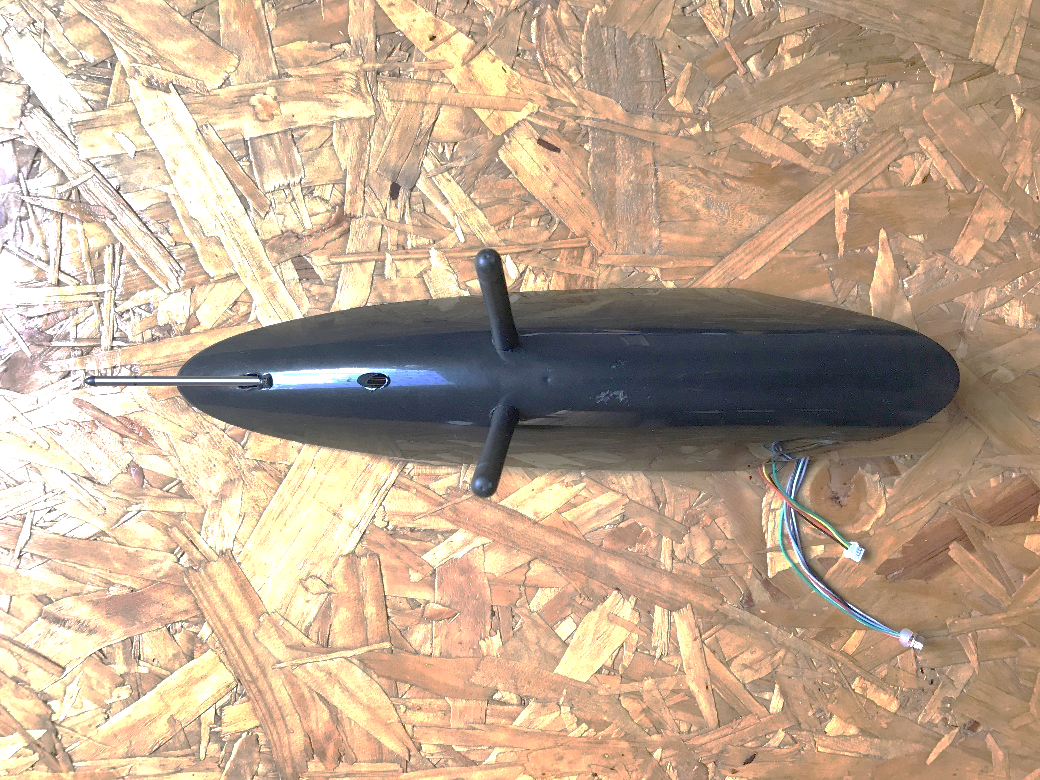

Supplement: S2 Text — (DOCX) [file pone.0255559.s004.docx]
