# Supplementary material for: Steps to build a DIY low-cost fixed-wing drone for biodiversity conservation
Source: PLoS One. 2021 Aug 13;16(8):e0255559. doi: 10.1371/journal.pone.0255559 (PMC8363011; doi:10.1371/journal.pone.0255559)

**# Setting up – guidelines on settings #**

1. Testing the main components

Before addressing the configuration inside the Ground Station Application, we have to check if the basic components (servos, motor, ESC) of Asa-Branca-I are working and are correctly calibrated. In order to check this, we will need to perform a test connecting the servos and motors directly to the RC receiver, following the cable connection in the same order as the Pixhawk (see item 1.2-k in S2 Text). In this test we should verify that the ailerons, elevator, rudder and flaps are centralized, and if necessary, carry out the adjustment manually in the respective servos. To calibrate the ESC when connecting the RC receiver to the battery, the throttle stick in the RC must be fully up. After two consecutive beeps, move the throttle stick all the way down.

1. Install Ground Control Station Application

The installation of a ground station application QGroundControl (QGC) is essential for Pixhawk's mandatory setup and calibrations for autonomous flights, in addition to serving as an interface for flight configurations. Follow the instructions below according to the specific operating system: <https://docs.qgroundcontrol.com/en/getting_started/download_and_install.html>

1. Firmware Update

Once the QGC is installed, access the software and then connect the Pixhawk to the computer via the USB port. Select the Gear icon (Vehicle Setup) and in the left side menu the “Firmware” option. It is necessary to disconnect and reconnect the Pixhawk to start the firmware upgrade. We recommend the installation of PX4 Flight Stack choosing the option that has “Stable Release”, as shown in the image below.

More information: <https://docs.qgroundcontrol.com/en/SetupView/Firmware.html>


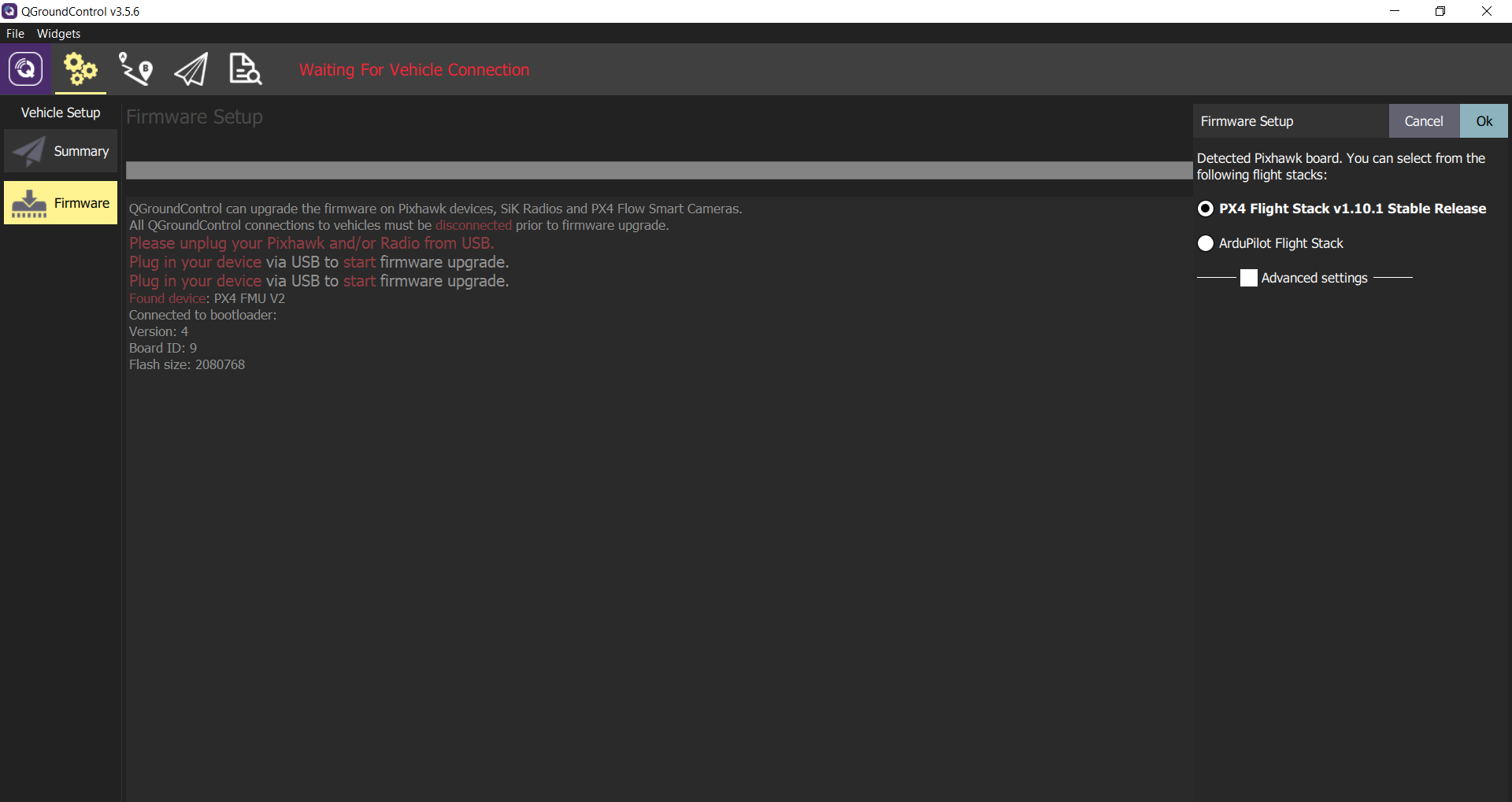


1. Airframe Setup

After installing the Firmware, it is necessary to choose the type of airframe compatible with our vehicle. This is done in the “Vehicle Setup” menu, choosing the “Airframe” option and then searching for the airframe compatible with your vehicle. Once selected, click on the Apply and Restart option in the upper right corner. After finishing the process, it is necessary to restart Pixhawk by disconnecting and connecting again.

More information: <https://docs.qgroundcontrol.com/en/SetupView/airframe_px4.html> and <https://docs.px4.io/master/en/airframes/airframe_reference.html>


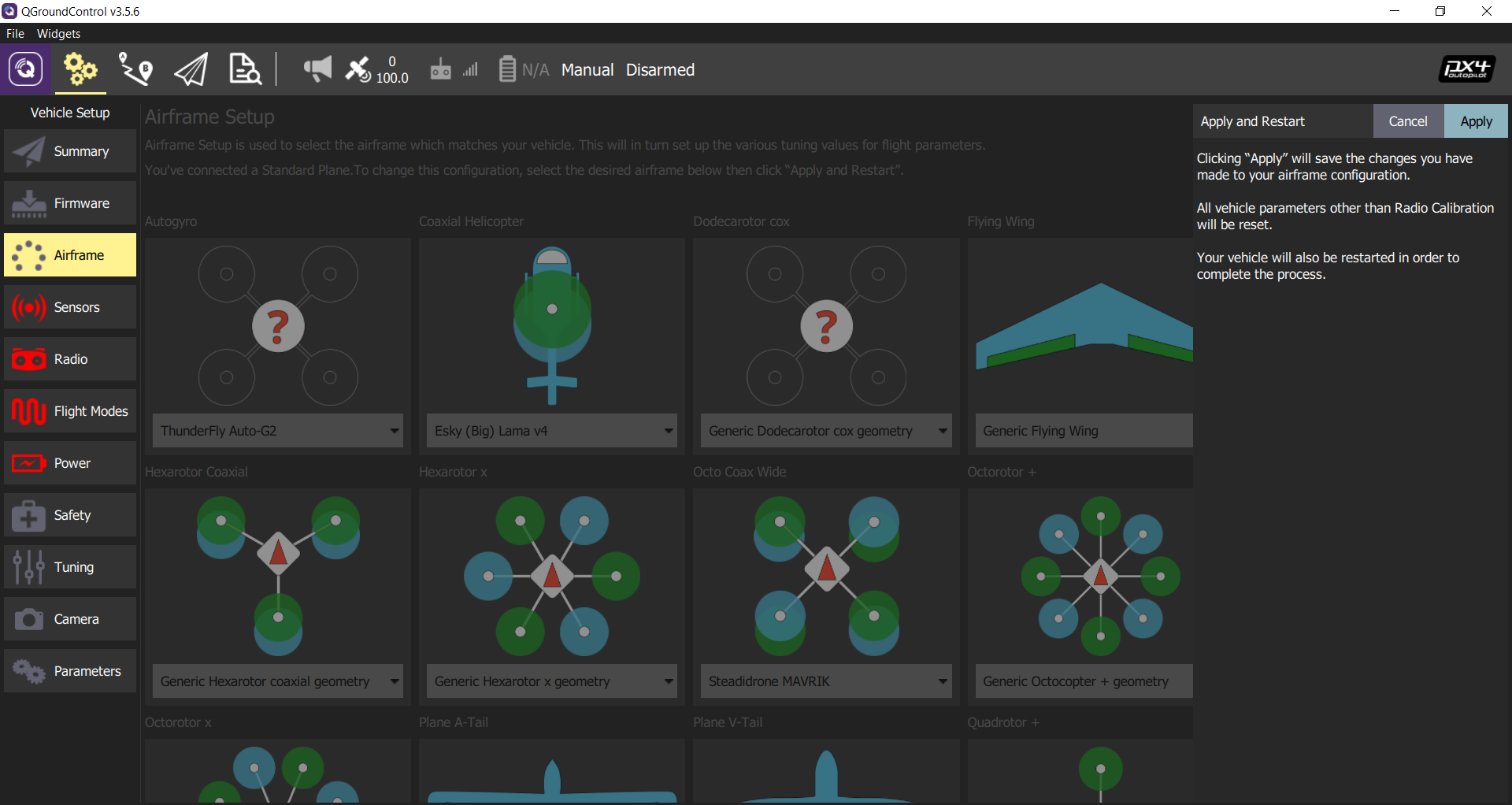


1. Sensor Setup

All configurations and calibrations within the “Sensors” menu, except “Level Horizon” are recommended to be performed with the Pixhawk outside the airframe to facilitate movement in the calibration process. As we also want to calibrate the magnetometer / compass of the GPS module, it will be necessary to connect the GPS to the Pixhawk before starting the calibration processes below. The orientation of the GPS must follow the same orientation as the Pixhawk in these processes, that is, the arrow points towards the front of the vehicle. We recommend that theses process be done location away from large metal objects or magnetic fields.

More information: <https://docs.qgroundcontrol.com/en/SetupView/Firmware.html>

- 1. Set Orientations

Considering that we have changed the Pixhawk's default position due to changes in the internal part of the model Asa-Branca-I, it will be necessary to carry out the change also in the Autopilot. Within the “Vehicle Setup” in the “Sensors” option, choose the last option “Set Orientations”. Then choose the orientation according to the Pixhawk's position on the airframe, in this case “ROTATION_ROLL_180”. It is necessary to restart the vehicle by clicking on the “Reboot Vehicle” option.

More information: <https://docs.px4.io/master/en/config/flight_controller_orientation.html>


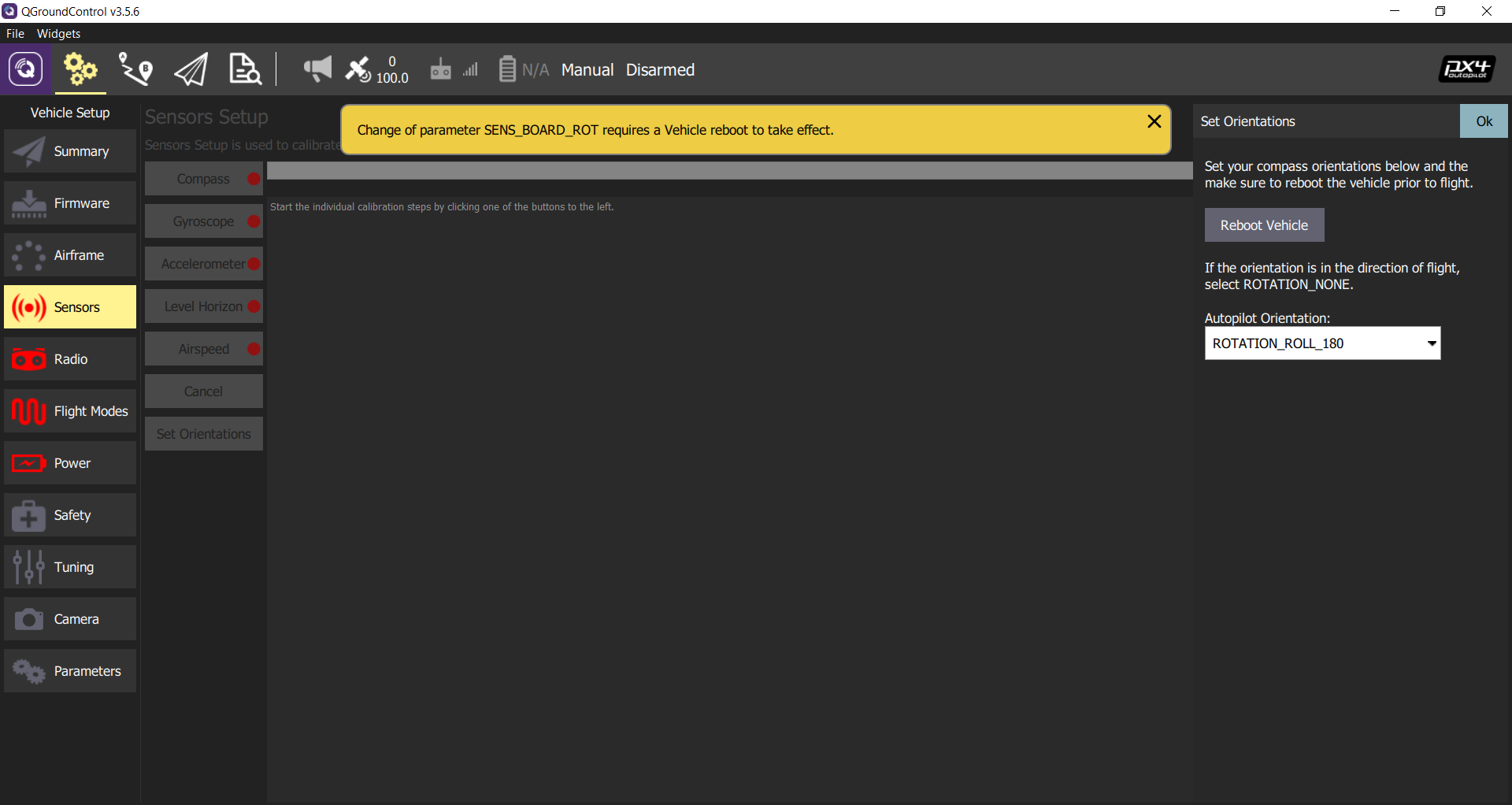


- 1. Compass Calibration

Once the Pixhawk orientation has been defined according to the position on the airframe, the Compass calibration process starts. Within the “Vehicle Setup” in the “Sensors” option, choose the last option “Compass”. Follow the instructions changing the Pixhawk's position as indicated.

More information: <https://docs.px4.io/master/en/config/compass.html>


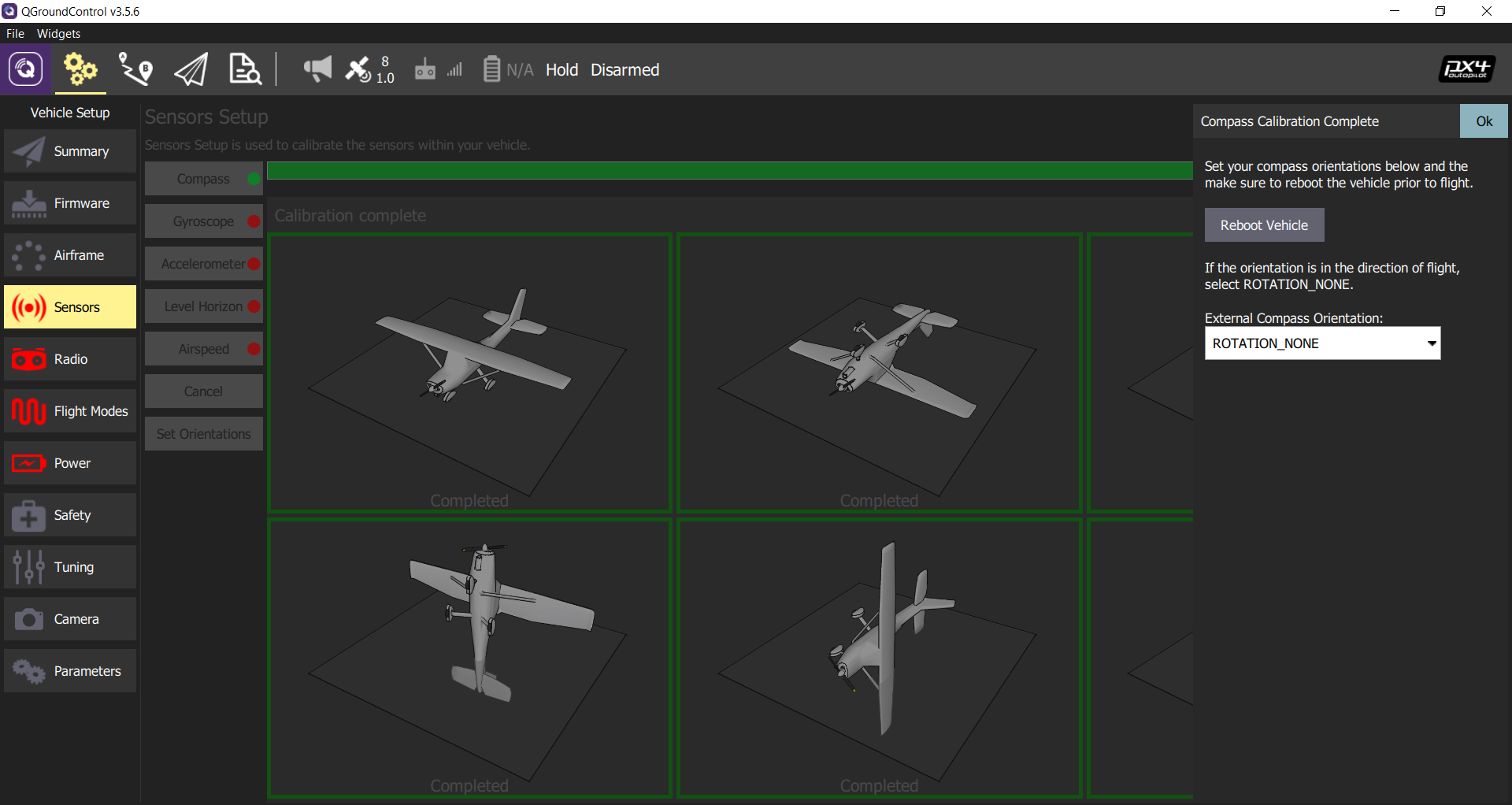


- 1. Gyroscope Calibration

The gyroscope calibration process is the simplest of all. Within the “Vehicle Setup” in the “Sensors” option, choose the last option “Gyroscope”. Follow the instructions and just leave the Pixhawk on a flat surface and keep it still till the end of the process.

More information: <https://docs.px4.io/master/en/config/gyroscope.html>


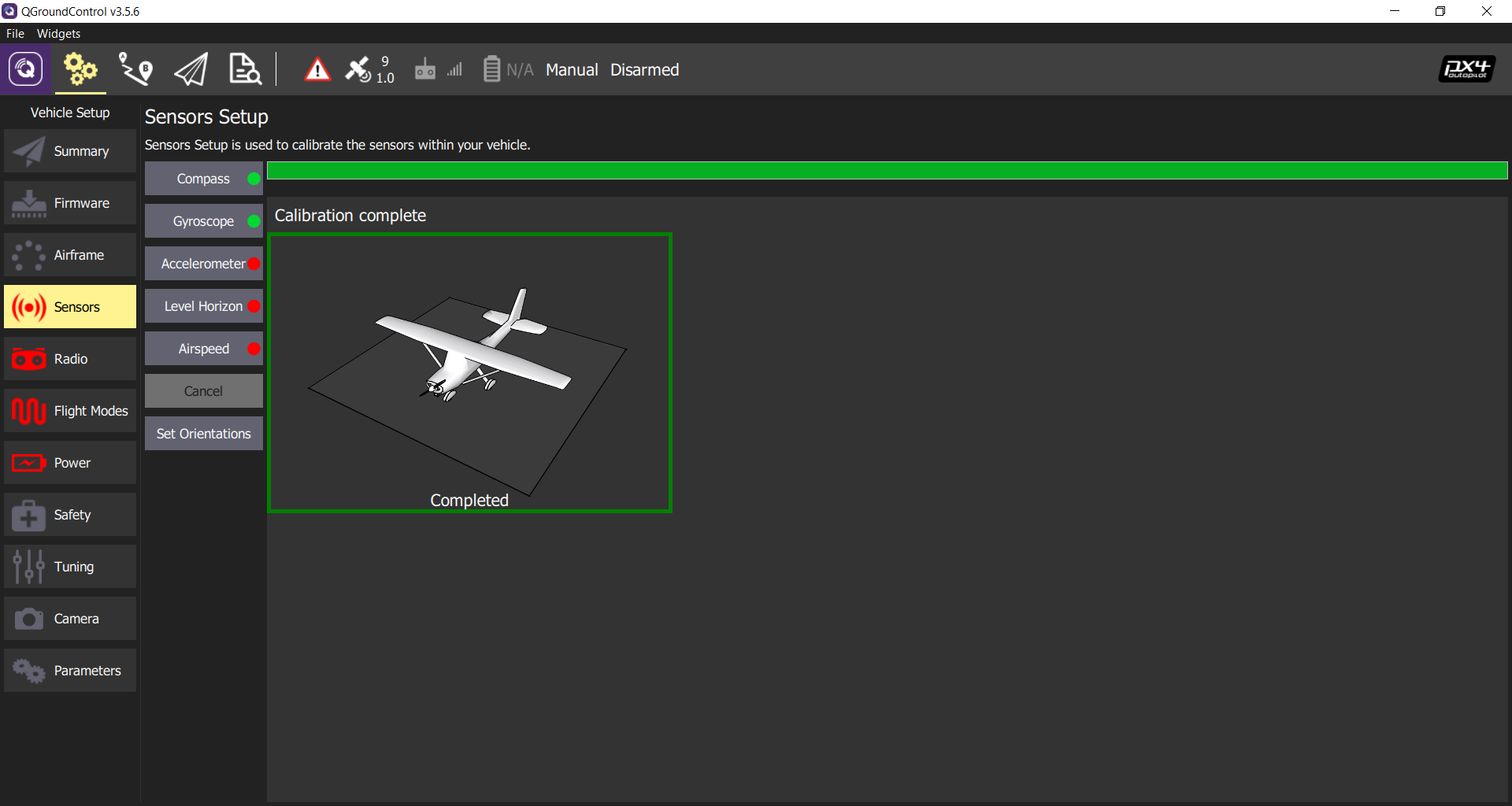


- 1. Accelerometer Calibration

Similar to Compass Calibration, the Accelerometer Calibration should be done every time there is a change in the Pixhawk's orientation on the airframe. Within the “Vehicle Setup” in the “Sensors” option, choose the last option “Accelerometer”. Follow the instructions changing the Pixhawk's position as indicated.

More information: <https://docs.px4.io/master/en/config/accelerometer.html>


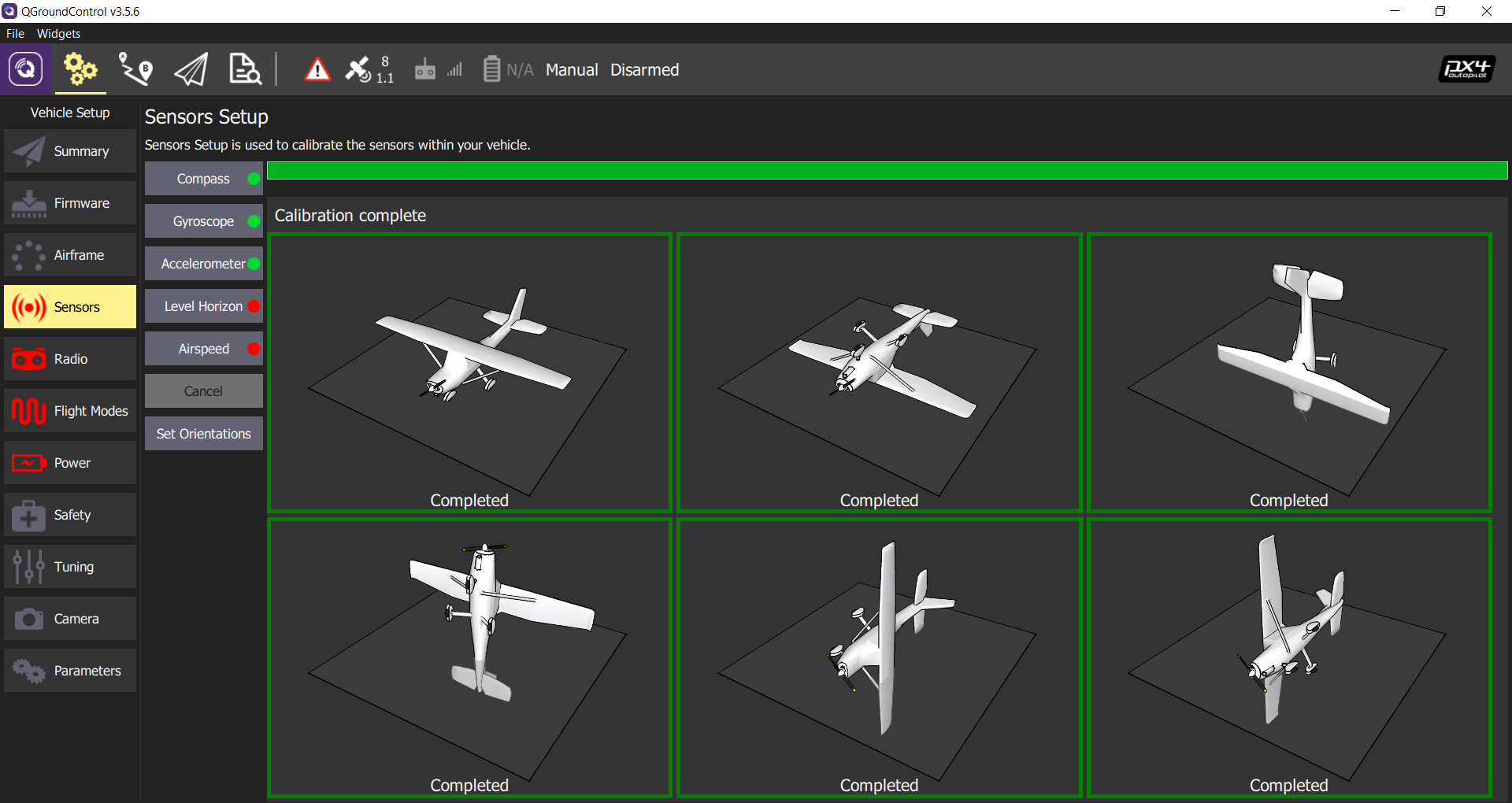


- 1. Level Horizon Calibration

This is an additional calibration, but important to compensate for minor errors in the orientation of the Pixhawk. Within the “Vehicle Setup” in the “Sensors” option, choose the last option “Level Horizon”. Unlike previous calibrations, we recommend that this is done with the Pixhawk already mounted on the airframe, as it will be necessary to adjust according to the stabilized flight position.

More information: <https://docs.px4.io/master/en/config/level_horizon_calibration.html>


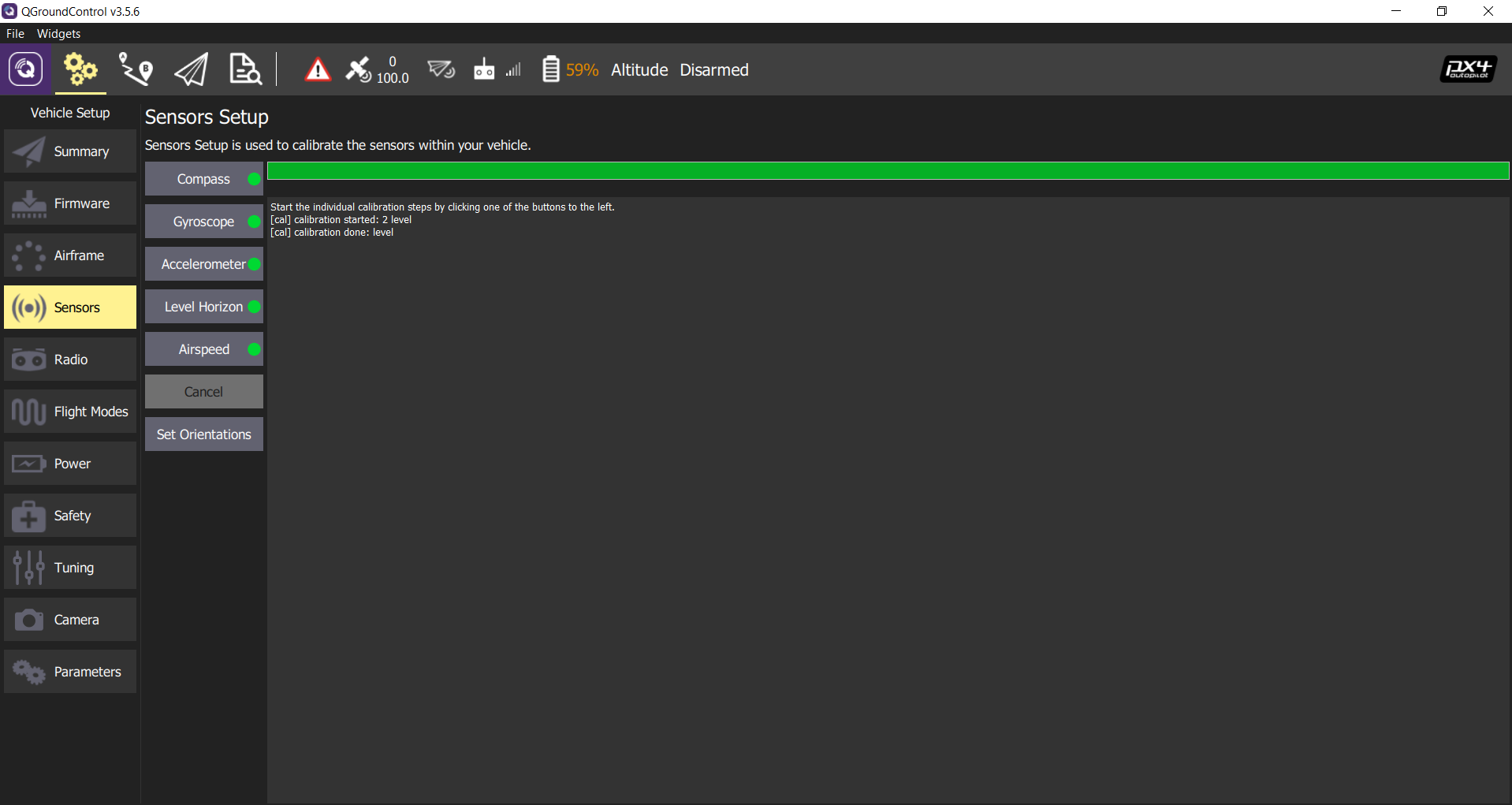


- 1. AirSpeed Calibration

The airspeed sensor calibration is essential for balance and efficiency of autonomous flight, mainly for fixed-wing airframes. Within the “Vehicle Setup” in the “Sensors” option, choose the last option “Airspeed”. Follow the instructions as indicated in the screen. It is recommended to perform the airspeed calibration in a closed environment without the influence of the wind.

More information: <https://docs.px4.io/master/en/config/airspeed.html>


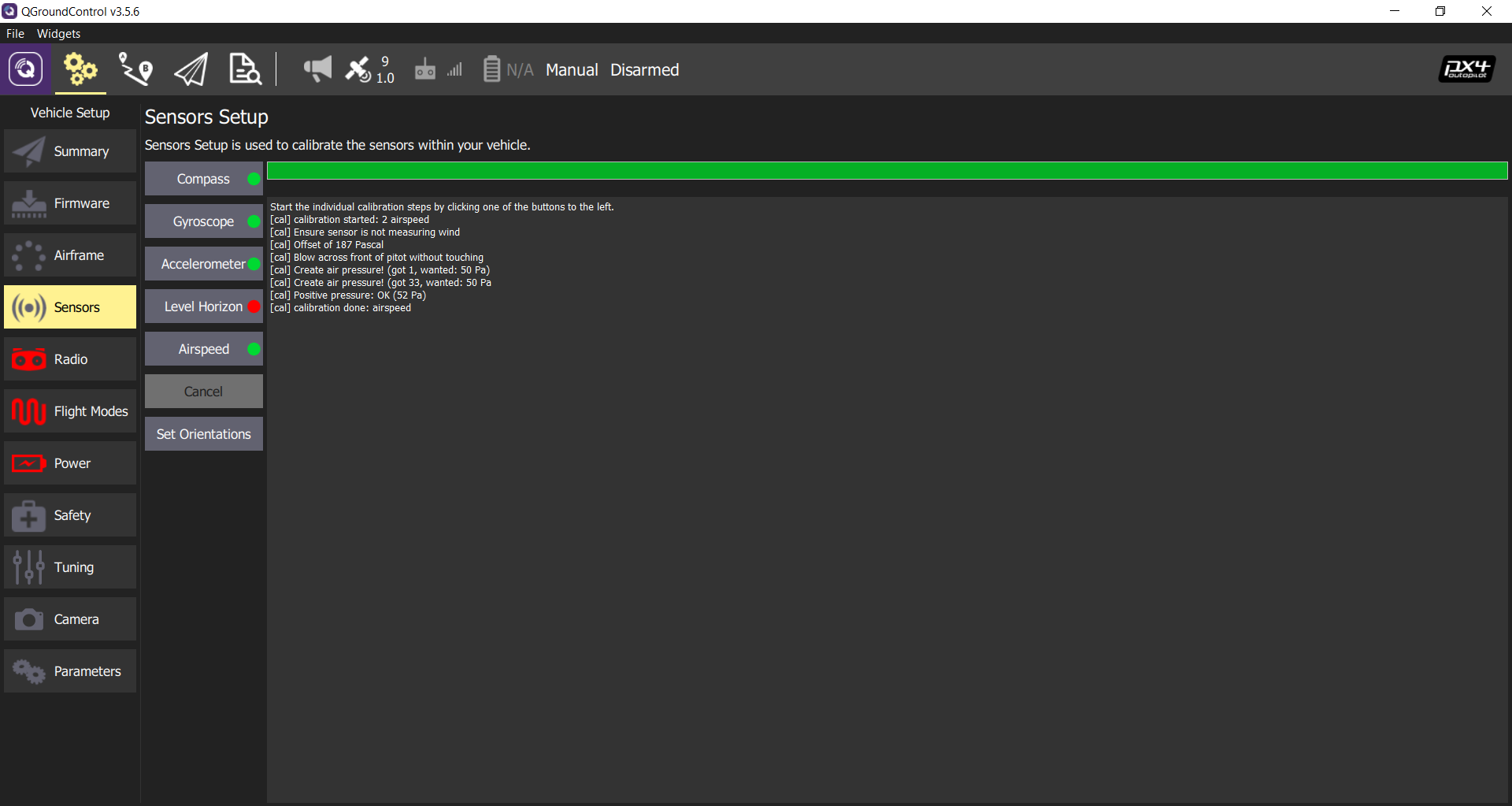


1. RC (Radio Control) Setup

Before starting the RC settings and calibrations, it will be necessary to make the connection between the receiver and transmitter of your radio (Binding). For this, we recommend reading the manual of each transmitter. We also recommend that channel configurations are made for possible manual flights. The RC setup in QGC serves to calibrate the maximum and minimum values of the control sticks (roll, pitch, yaw, throttle) to channels, trims and reverse settings. Within the “Vehicle Setup” in the “Radio” option, there are two main ways to control drones by RC. In this project we chose Mode 2, that is, the control sticks for controlling throttle / yaw are on the left side of the pilot. In this case, before starting the calibrations, we must choose Mode 2 in the upper right corner of the screen. With the RC connected, click on Calibrate and follow the instructions on the screen.

More information: <https://docs.px4.io/master/en/config/radio.html>


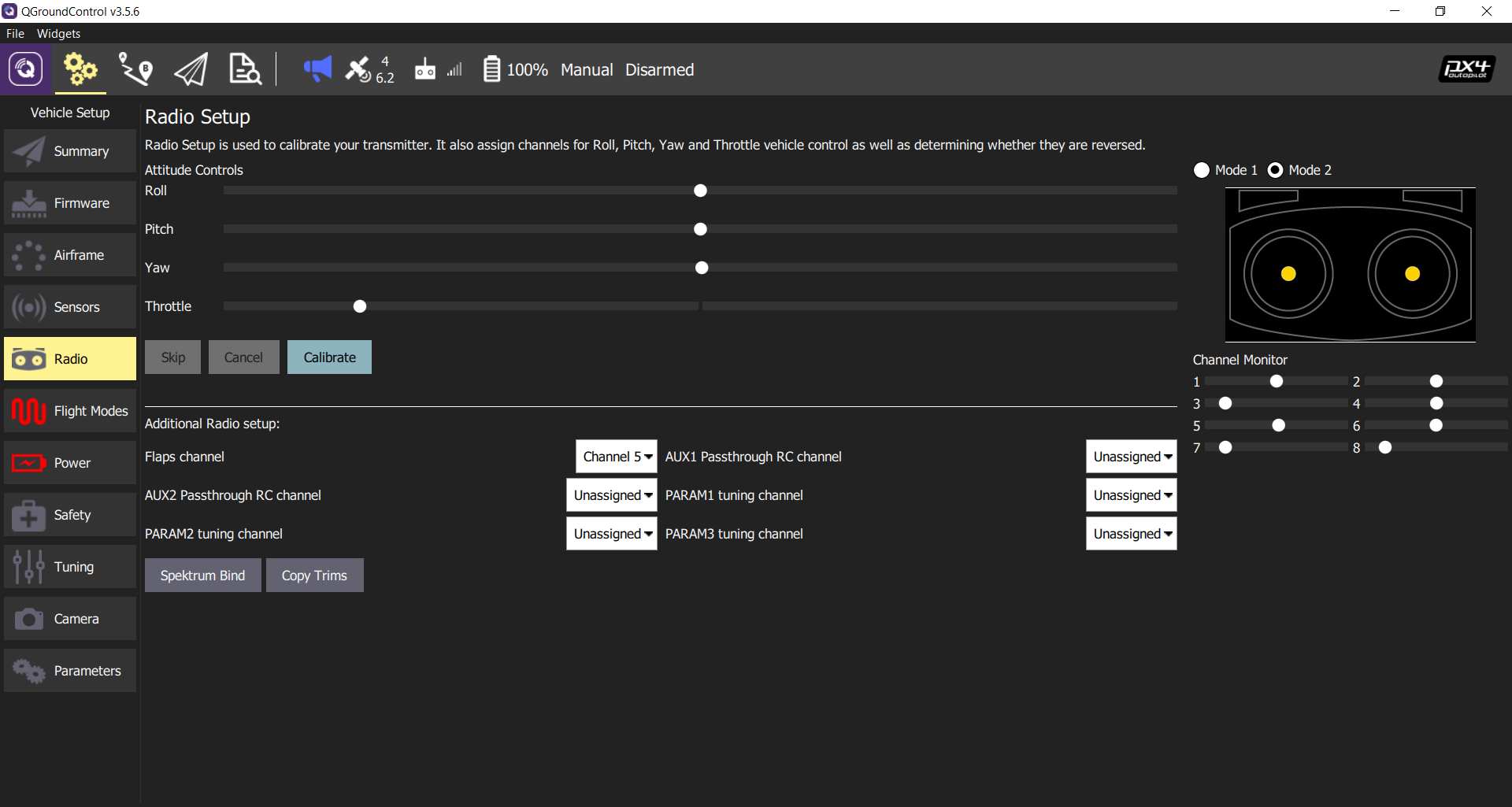


1. Flight Modes Configuration

The flight modes are different types of fights in the QGC that allow the pilot to perform semi-autonomous flights with different levels of control and autonomous flights, in addition to allowing to set up different tasks automatically as landing and take-off. These different flight types are fundamental for the pilots that do not have experience with fixed-wing drones. The flight modes configuration can be made through a single RC channel or through several RC channels. In this project we choose to configure the Single Channel Mode Selection because it is easier to configure. In this case, we choose the channel 6 that is assigned to the VRB button in the RC Flysky. There are several configuration options according to each RC (see item 14 in S5 Text). The VRB button from Flysky is rotational witch allows to assign different flight modes in each value. Considering the clockwise of the VRB button we define 7 types of flight mode (Position, Altitude, Stabilized, Mission, Hold, Return and Land).

More information about flight modes: <https://docs.px4.io/master/en/getting_started/flight_modes.html>


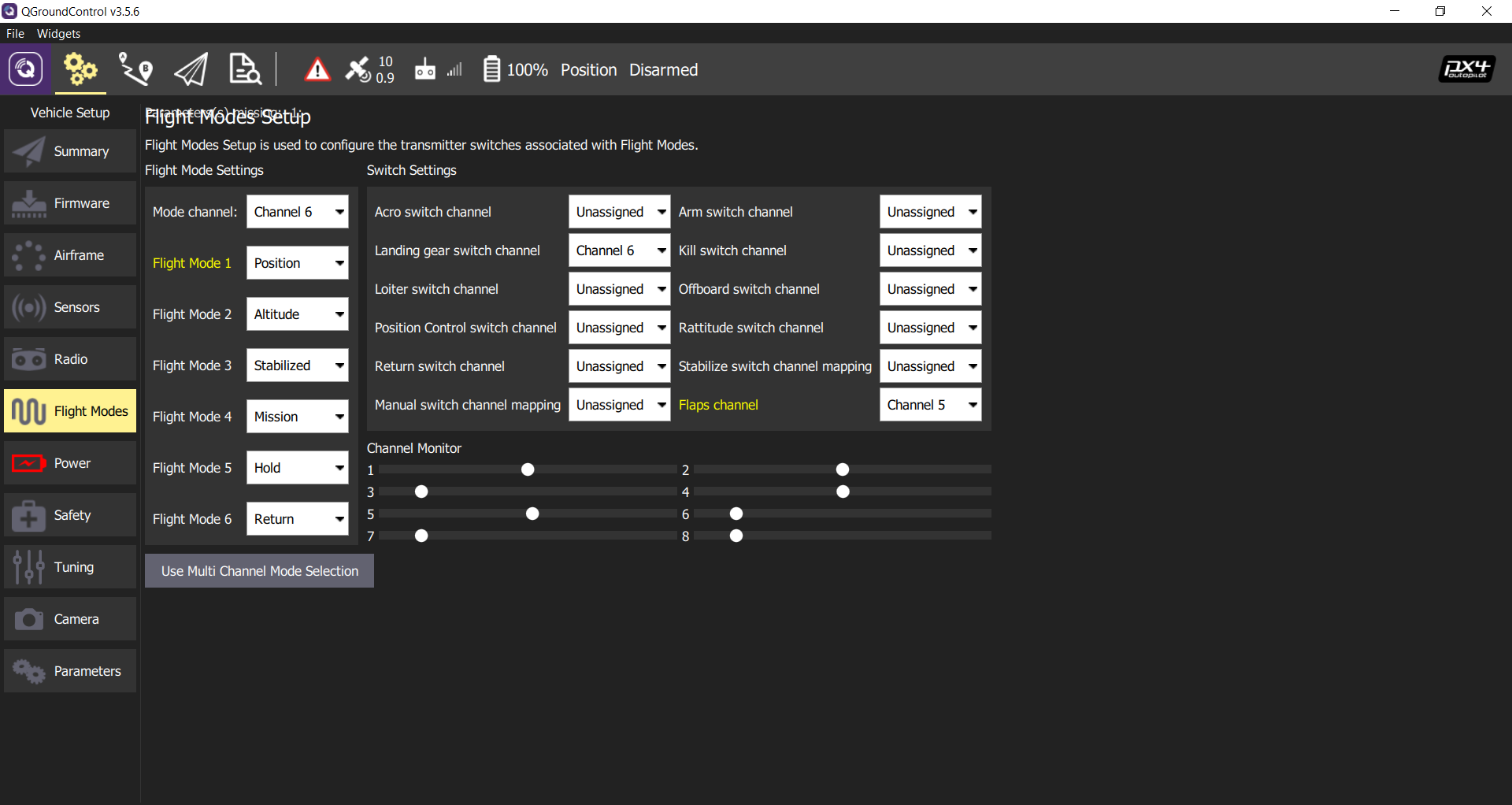


1. Power calibration

In the “Power” menu it is necessary to initially perform the ESC calibration and inform the battery specifications in the QCG. With the battery disconnected, click on the “Calibrate” option and follow the instructions to start the ESC calibration process. Then inform the number of cells of the battery used and restart to validate the settings. The number of cells can be seen in the battery specifications.

More information: <https://docs.px4.io/master/en/config/battery.html>


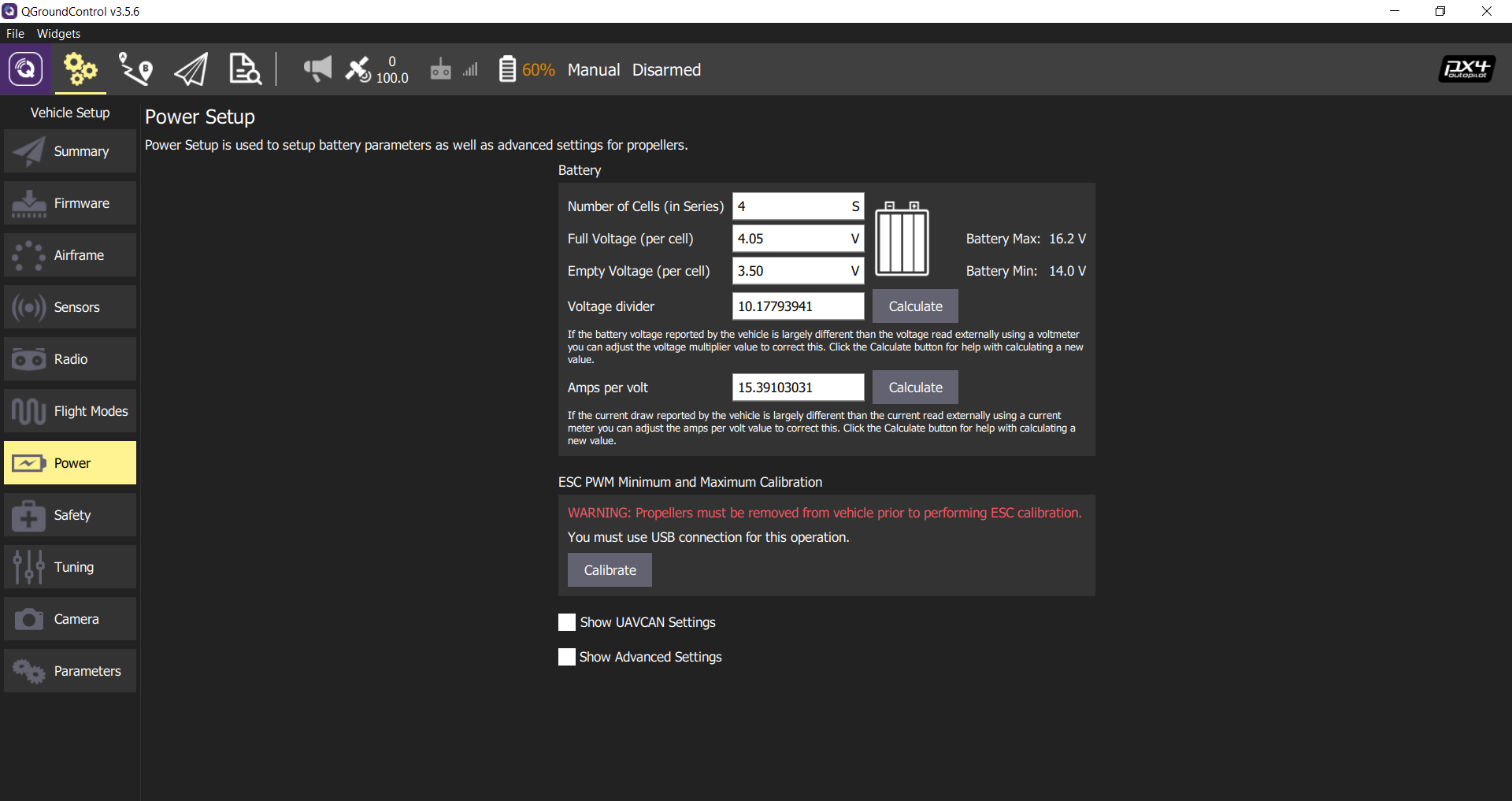


1. Safety configuration

Safety configurations are fundamental for the flight safety, especially in autonomous flights. On this screen, we will configure what the drone's behavior should be in the main critical situations such as low battery level, loss of RC signal, loss of telemetry signal or in cases of flights out of range. The security configurations proposed here are based on the experiences we obtained in the various flights performed with the model Asa-Branca-I, but we recommend that each one uses the security configurations according to the specific purposes and drone use.

In case of low battery level, we chose the option of “Return mode at critically low level, land mode at current position if reaching dangerously low levels”, keeping 15% for battery warn level, 10% for failsafe level and 5% for emergency level. In the event of loss of the RC signal, we disable this option since most autonomous flights over large areas will occur at distances greater than 1 km, that is the maximum reach between the RC and its receiver. For the loss of telemetry signal, we opted for the execution of “Return mode” in situations of signal loss of more than 10s. In this situation, the drone will return to a location defined in the mission, until the signal is recovered. We also opted for “Return mode” in the Geofence Failsafe Trigger when the drone reaches a radius maximum of 20 km or maximum altitude of 400 m, however, this Geofence Failsafe can be adapted for each flight mission. For the Return Home settings, we choose as 75 m the climb and loiter altitude and the option loiter and land after specified time with 60 s. We keep the default altitude of 2 m for disarm motor in the Land Mode option.

More information: <https://docs.px4.io/master/en/config/safety.html>


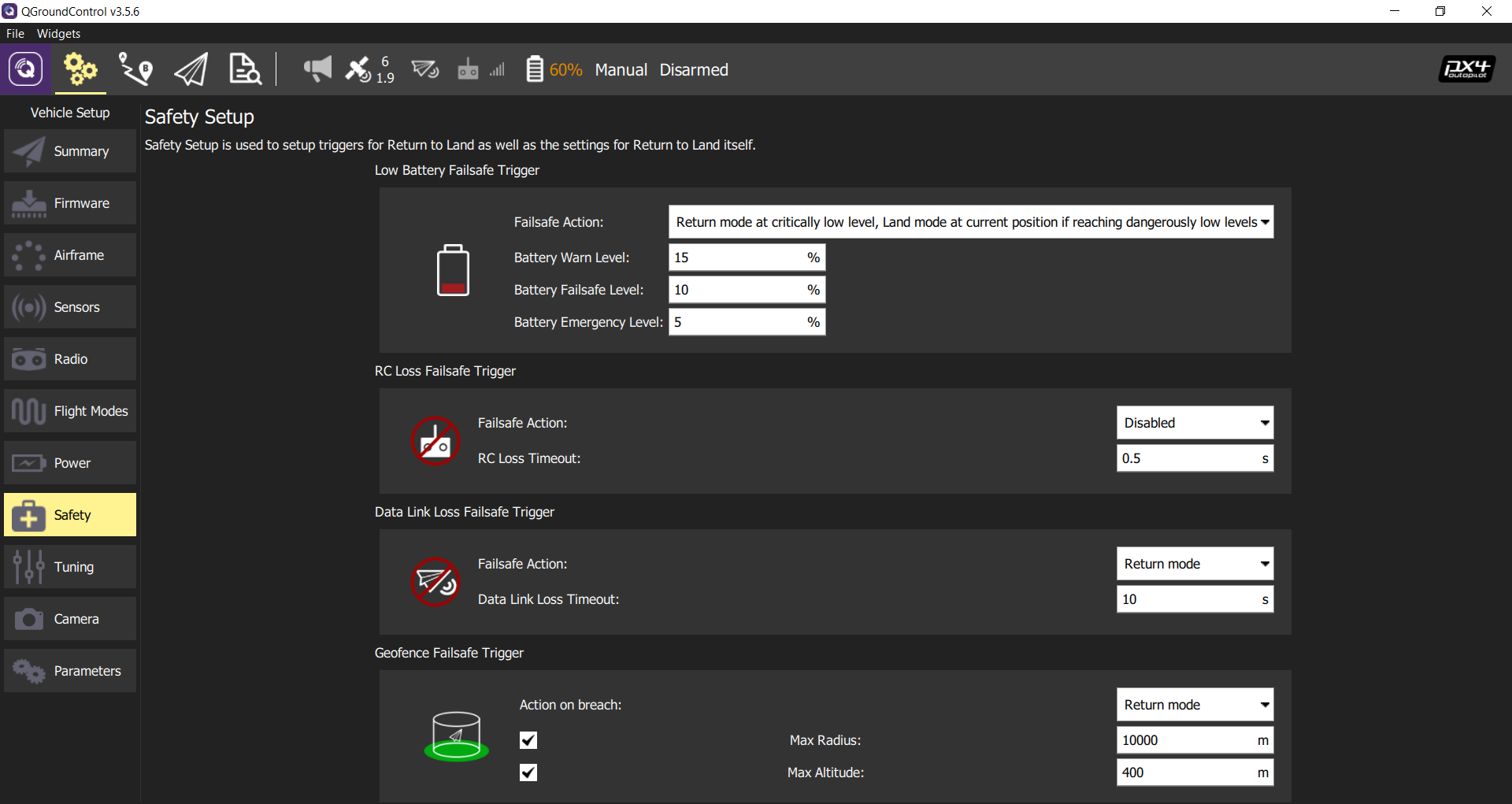


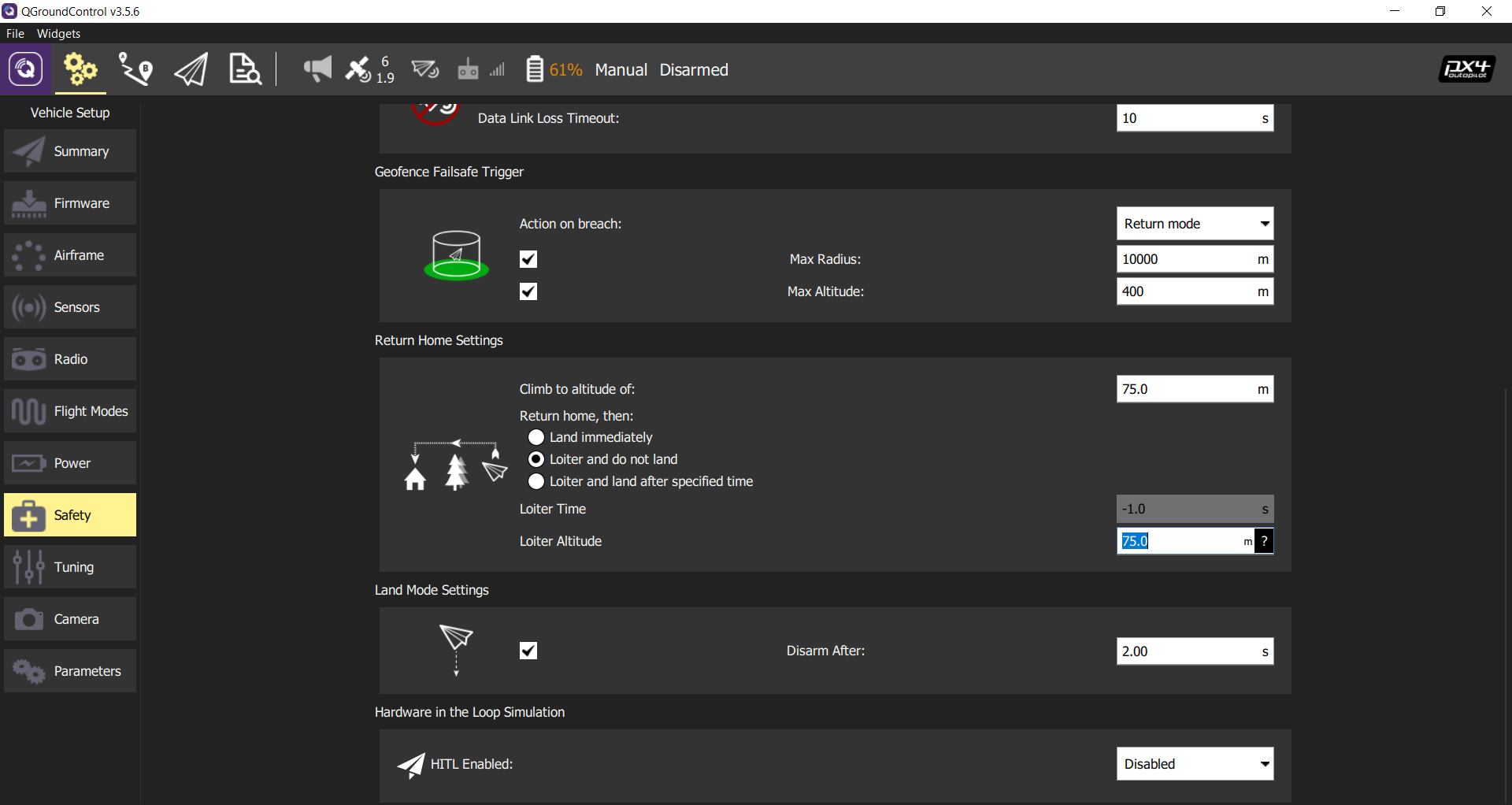


1. Camera Setup

With the camera installed on the Pixhawk, we can define different types of camera trigger, by sending pulses to the Pixhawk auxiliary ports, in this case, ports 5 and 6 (see item 1.2-I in S2 Text). Considering the different types of drone use, we can choose different trigger modes. For this project we choose the option “Distance based, on command (Survey mode)” since the intention is to use it in flight missions in biodiversity projects. It is possible to select different types of trigger interface backends that vary according to the type of sensor (camera) in use. In this project we use the Seagull MAP2 interface, which is already preconfigured as the option “Seagull MAP2 (over PWM) in QGC.

More information: <https://docs.px4.io/v1.10/en/peripherals/camera.html>


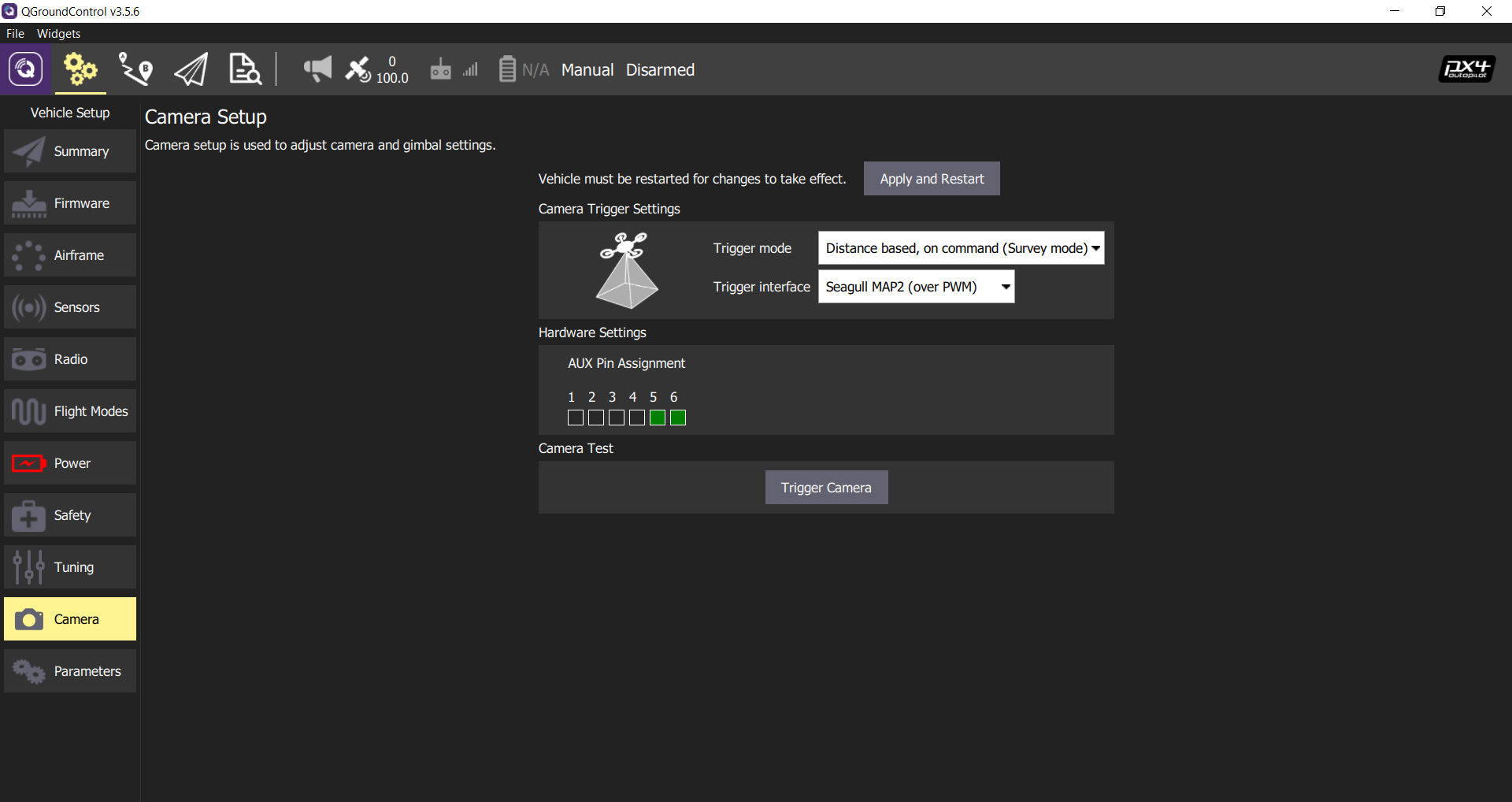


If all settings have been made correctly, the QGS summary should appear with all green circles and with all side menus not red, as shown in the image below. It is worth recalling that until now, all settings and calibrations have been performed via a USB connection between Pixhwak and QGC. Once the above configurations are made, we can connect the telemetry to the QGC, which should be automatically recognized by the Pixhawk.

From here, it is already possible to start flight tests manually or automatically and the settings for flight missions within the “Plan” menu (patch A-B icon) in the QGC.


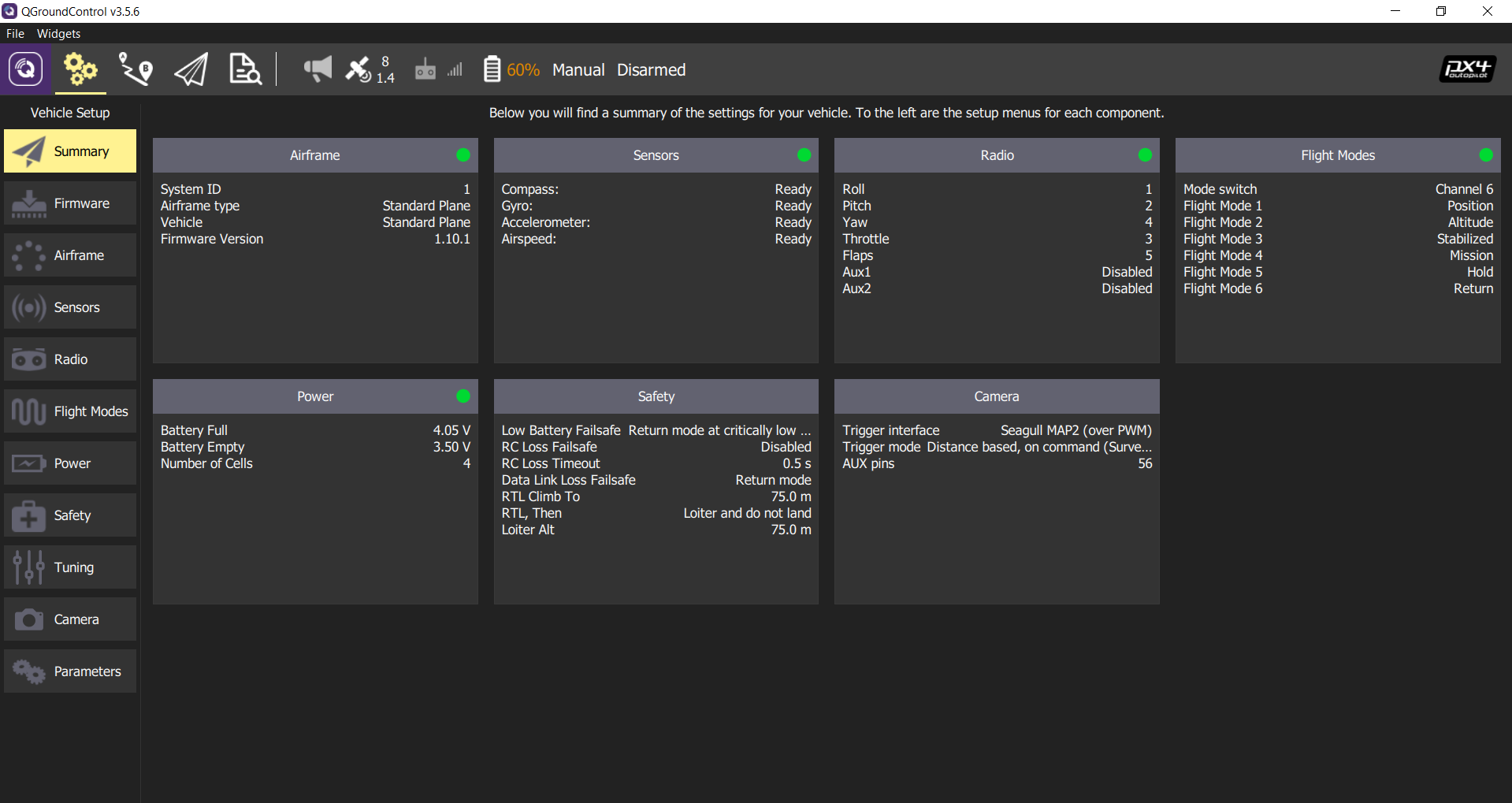

Supplement: S3 Text — (DOCX) [file pone.0255559.s005.docx]
