# Supplementary material for: Steps to build a DIY low-cost fixed-wing drone for biodiversity conservation
Source: PLoS One. 2021 Aug 13;16(8):e0255559. doi: 10.1371/journal.pone.0255559 (PMC8363011; doi:10.1371/journal.pone.0255559)
Supplement: S4 Text — (DOCX) [file pone.0255559.s006.docx]

**# Tips and Troubleshooting #**

The development of a fixed-wing drone using DIY concepts without previous experience can become slightly complicated, but in our experience, mainly due to lack of attention or mistakes related to electronics. We suggest trying to avoid such problems by checking, performing the tests and documenting each step to record and eventually review what was done. Here we present the main identified problems and offer suggestions to solve them.

- Weight, Power and Wing loading

In fixed-wing drones, a fundamental factor to considered is the Wing loading, which is the index resulting from the weight of the aircraft divided by the wing area, usually measured by the unit (g/dm^2^). It is very important to have a balance between the total weight of the drone (including payload), the engine power and the size of the airframe wing, since this balance will directly influence flight performance and flight autonomy. There is no standard formula that defines a balance between battery capacity, engine, weight and flight time, but it is necessary to consider several factors (flight type, airframe model, wing load, engine rpm/v, propeller size, and others) to find an optimal compromise. Therefore, before assembling the fixed-wing drone, we recommend reading about Wing loading, considering the flight type and the mission goals.

- Internal position of components and Gravity Center

Once the airframe, battery and all other drone payloads are defined, we need to check the position of the internal components considering the airframe's center of gravity. Generally, fixed-wing airframes have marks indicating of the center of gravity on the underside of the wings. Before starting any change in the internal structure of the airframe, we recommend simulating the positions of the internal components, especially those with greater weight, such as battery and camera, considering the balance based on the measurement of the center of gravity of the two wings.

- Basic components testing

The basic components mentioned here are those that allow manual flight without the need for a flight controller. The engine, servos, ESC and RC receiver are the necessary components into the drone that allow its manual control. The proper functioning of the basic components will avoid possible problems in automatic flights. We recommend testing the basic components and performing manual flights without the flight controller installed to check the drone's maneuverability.

- AirSpeed sensor position

The position of the Airspeed sensor tube can affect the operation of the airspeed sensor during the flight and consequently affect the performance of autonomous flights. We recommend that the pitot tube is installed in a location where there are no direct physical barriers between the wind and the tube and that it is pointing directly into the air stream, preferably passing at least 1 cm beyond the nose of the drone.

- GPS/Compass position

In the first autonomous flight tests we verified some interference with the GPS / Compass. After changing the position of the external GPS / Compass, we found that the proximity to the engine affected the performance of the GPS / Compass. We recommend that the GPS / Compass is positioned away from the engine to avoid magnetic interference. In this project, we positioned the GPS at a distance of 17 cm from the engine, which eliminated the interferences identified in the first autonomous test flights.

- Vibration and movement of components

During the test flights we almost had a drone crash due to the Pixhawk's internal displacement which affected the performance of the autonomous flight. To solve this problem, we installed 4 rubber vibration dampers balls to the bottom of the Pixhawk (see item 2.3 in S2 Text). We also noticed the same vibration problem with the RGB sensor (Sony model DSC-HX50) that we solved by installing foam between the camera and the airframe.

- Pixhawk upgrade firmware

During the testing of the firmware update process, we had a card initialization problem. At the end of the firmware update process (see item 3 in S3 Text) the Pixhwak board was unable to initialize. In this situation we had to perform the Bootloader Update which consists of reinstalling the PX4 firmware in the master version, clicking on the advanced settings option. After the master version is installed and reboot by QGC, we search for the SYS_BL_UPDATE parameter in the “Vehicle Setup” menu and “Parameters” submenu and change to “Enable”. Then, it will be necessary to restart Pixhawk (disconnect / reconnect the board) and access QGC again for the Bootloader Update to update. After this process, just perform the firmware update again (see item 3 in S3 Text).

- Pixhawk and Memory Card

After all the Pixhawk setting up process informed in the document “Setting up – guidelines on settings” we were unable to arm the drone. We detected that the reason for this problem was due to a problem with the SD card. To solve this problem, it was necessary to format the SD card as well as reset the Pixhawk and perform the procedures in “Setting up” again. If you are using an SD previously used, we recommend formatting it in FAT32 format, before starting the Pixhawk settings. In addition, we recommend using a high-quality SD card as any problem in the SD can affect the entire operation of the flight plans and system logs.

- Pixhawk Setting Up

We found that in the Pixhawk setting up process informed in “Setting up - guidelines on settings” when performed without the external GPS / Compass connected, there were some interference between the Pixhawk's GPS / Compass and the NEO-M8N GPS Module. We recommend that all the procedures informed in item 5 in “Setting up - guidelines on settings” be carried out with the connected GPS module to avoid this possible problem.

- RFD 900 telemetry connection failed

During the development and testing process of the drone we had to change our laptop (Ground Control Station - GCS). On the new laptop we had problems connecting Pixhawk with the GCS via telemetry (Sik radio) via QGroundControl software. We found that in some versions of Windows there may be problems with the FTDI Serial Port drives responsible for telemetry recognition. If this problem occurs, it will be necessary to downgrade the drives in the Windows. We recommend installing 6.7.5.1893 version instead of version 10.1.1 according to the following link: <https://www.silabs.com/products/development-tools/software/usb-to-uart-bridge-vcp-drivers>
