# Supplementary material for: Steps to build a DIY low-cost fixed-wing drone for biodiversity conservation
Source: PLoS One. 2021 Aug 13;16(8):e0255559. doi: 10.1371/journal.pone.0255559 (PMC8363011; doi:10.1371/journal.pone.0255559)
Supplement: S5 Text — (DOCX) [file pone.0255559.s007.docx]

**# Help and Learning Links #**

1. Basic electronic components and functions

<https://www.youtube.com/watch?v=j61Q3e8AFR4&feature=em-share_video_user>

1. Pixhawk Overview

<https://ardupilot.org/plane/docs/common-pixhawk-overview.html>

1. Led and Sounds Pixhawk

<https://ardupilot.org/plane/docs/common-leds-pixhawk.html>

1. Ublox GPS + Compass Module

<https://ardupilot.org/plane/docs/common-installing-3dr-ublox-gps-compass-module.html?highlight=ublox>

1. Manual Seagull MAP2

<https://www.seagulluav.com/manuals/Seagull_MAP2-Manual.pdf>

1. IMAX B6 Charger: Overview and basic usage

<https://www.youtube.com/watch?v=w8ijfcjU-rc>

1. Design of flying wing UAV

<https://www.researchgate.net/figure/Fig-4-Power-Loading-vs-Wing-Loading_fig1_325070398>

1. Possible programming for manual flights on RC Flysky FS i6
   <https://www.youtube.com/watch?v=xt5IdeLdiug>
2. ESC Manual Calibration

<https://ardupilot.org/copter/docs/esc-calibration.html>

1. Plan view for Autonomous missions

<https://docs.qgroundcontrol.com/en/PlanView/PlanView.html>

1. Autopilot Hardware Options

<https://ardupilot.org/plane/docs/common-autopilots.html>

1. Choosing a Ground Station

<https://ardupilot.org/plane/docs/common-choosing-a-ground-station.html>

1. Balance and center of gravity

<https://www.youtube.com/watch?v=0LhYonZg6RY>

1. Fight Mode Configuration

<https://docs.px4.io/master/en/config/flight_mode.html>
