# Supplementary material for: Steps to build a DIY low-cost fixed-wing drone for biodiversity conservation
Source: PLoS One. 2021 Aug 13;16(8):e0255559. doi: 10.1371/journal.pone.0255559 (PMC8363011; doi:10.1371/journal.pone.0255559)
Supplement: S1 Checklist — (DOCX) [file pone.0255559.s008.docx]

| **Pre-flight Checklist** | | |
| --- | --- | --- |
| Location: | FAA Reg. No.: | Date: |
| Drone Model: |  |  |
| Pilot in Command: |  |  |

| **Mechanical Assembly** | | |
| --- | --- | --- |
| **Item** | **Conditions** | **Check** |
| sUAS Airframe/Propellers | No structural defects visible |  |
| Electrical connectors | No structural defects visible |  |
| Control surfaces | Responds correctly |  |
| Pitot tube | Extended straight, clear of debris |  |
| Drone Battery | Sufficient for intended flight, >75% |  |
| Drone connections | Connected |  |
| Laptop Battery | Sufficiently charged |  |
| Radio Control Battery | Sufficiently charged |  |
|  | | |
| **Field Set-up** | | |
| **Item** | **Conditions** | **Check** |
| Airspace | Unrestricted airspace or flight authorized |  |
| Weather | No precipitation and wind <= 25km/h |  |
| Take-off and landing area | Glide path and clear of obstacles |  |
| Ground radio | Powered ON |  |
| Antenna | Mounted and clear view |  |
|  | | |
| **Flight Plan** | | |
| **Item** | **Conditions** | **Check** |
| Take-off waypoint | Into the wind, clear of obstacles |  |
| Landing waypoint | Glide path with 100m, clear of obstacles |  |
| Area waypoint(s) | Set correct altitude and overlap |  |
| Plane Failsafe Function | Set correct to return home |  |
|  | | |
| **Sensors** | | |
| **Item** | **Conditions** | **Check** |
| GPS lock | Minimum 7 satellites, GPS quality values Green |  |
| Point Drone North, East, South, West | verify "Magnetometer" reading and airplane icon |  |
| Pitot (finger test) | airspeed >30 km/h for 3 seconds |  |
| Shield pitot | airspeed 0 +/- 10 km/h |  |
| Camera Check | Test camera trigger |  |
| Hover Check | Flight and Camera Gimbal control responses normal |  |
| Memory card camera | Installed, sufficient memory space available for flight |  |
|  | | |
| **Launch** | | |
| **Item** | **Conditions** | **Check** |
| Compass Calibration | Compass calibrated for current location |  |
| Home point | Home point set |  |
| Drone motor Start | Drone motors start and run at idle, no abnormal noise |  |
| Flight Telemetry | Telemetry normal (Bat, Alt, Dist., etc.) |  |
| Drone Status Lights | Flashing GREEN |  |
